# Cutaneous squamous cell carcinoma characterized by MALDI mass spectrometry imaging in combination with machine learning

**DOI:** 10.1038/s41598-024-62023-0

**Published:** 2024-05-15

**Authors:** Lauritz F. Brorsen, James S. McKenzie, Mette F. Tullin, Katja M. S. Bendtsen, Fernanda E. Pinto, Henrik E. Jensen, Merete Haedersdal, Zoltan Takats, Christian Janfelt, Catharina M. Lerche

**Affiliations:** 1 Department of Dermatology, Copenhagen University Hospital - Bispebjerg and Frederiksberg, Nielsine Nielsens Vej 9, 2400 Copenhagen, Denmark; 2 Department of Pharmacy, University of Copenhagen, Copenhagen, Denmark; 3 Department of Digestion, Metabolism and Reproduction, Imperial College London, London, UK; 4 Department of Veterinary and Animal Sciences, University of Copenhagen, Copenhagen, Denmark; 5 Department of Clinical Medicine, University of Copenhagen, Copenhagen, Denmark

**Keywords:** Squamous cell carcinoma, Machine learning, Cancer imaging, Cancer metabolism, Skin cancer, Tumour biomarkers, Surgical oncology, Cancer imaging, Skin cancer, Imaging studies, Mass spectrometry, Medical and clinical diagnostics

## Abstract

Cutaneous squamous cell carcinoma (SCC) is an increasingly prevalent global health concern. Current diagnostic and surgical methods are reliable, but they require considerable resources and do not provide metabolomic insight. Matrix-assisted laser desorption/ionization mass spectrometry imaging (MALDI-MSI) enables detailed, spatially resolved metabolomic analysis of tissue samples. Integrated with machine learning, MALDI-MSI could yield detailed information pertaining to the metabolic alterations characteristic for SCC. These insights have the potential to enhance SCC diagnosis and therapy, improving patient outcomes while tackling the growing disease burden. This study employs MALDI-MSI data, labelled according to histology, to train a supervised machine learning model (logistic regression) for the recognition and delineation of SCC. The model, based on data acquired from discrete tumor sections (n = 25) from a mouse model of SCC, achieved a predictive accuracy of 92.3% during cross-validation on the labelled data. A pathologist unacquainted with the dataset and tasked with evaluating the predictive power of the model in the unlabelled regions, agreed with the model prediction for over 99% of the tissue areas. These findings highlight the potential value of integrating MALDI-MSI with machine learning to characterize and delineate SCC, suggesting a promising direction for the advancement of mass spectrometry techniques in the clinical diagnosis of SCC and related keratinocyte carcinomas.

## Introduction

Keratinocyte carcinoma (KC), also known as non-melanoma skin cancer, is the most common form of cancer affecting around 3.5 million people globally per year^1^. KC include basal cell carcinomas, Merkel cell carcinoma and cutaneous squamous cell carcinomas (SCC). While basal cell carcinomas are more common, SCC pose a greater threat to patients due to their rapid development, higher recurrence, and metastasis rates. Thus, despite its substantially lower incidence compared to basal cell carcinoma, SCC is associated with three-quarters of KC-related deaths^2,3^. In Caucasian populations KC is the most frequently diagnosed malignancy and the incidence rate is increasing^4^. Some models even suggest a doubling in the incidence of KC within the next 10 years^1^. Most cases of KC occur on skin areas that are exposed to ultraviolet radiation from sunlight^5^. Factors involved in the observed increase in incidence rate, include climate change, globalization, tanning beds, ageing populations as well as improved screening and reporting of the condition^2,4,6,7^. SCC has a considerable impact on the quality of life of patients^8,9^. Chronic inflammation and immunosuppression are often contributors to the development of SCC. Dysregulation of the lipid metabolism and signaling is fundamental in KC tumorigenesis^10,11^. Most SCCs originate from the precancerous lesion known as actinic keratosis; however, some cases arise de novo^6,12^.

In the majority of cases, surgical excision is the recommended treatment for SCC^9^. Among the various surgical approaches available, Mohs micrographic surgery is widely acknowledged as the most accurate^9,13^. This technique, first introduced in the 1940s, entails iterative steps of tumor excision, followed by histopathological evaluation, until complete removal of the tumor has been achieved^13^. Given the iterative nature of Mohs micrographic surgery, the procedure can be both time-consuming and costly. Furthermore, as Mohs micrographic surgery is relying on histology, the technique is susceptible to interobserver variation^14–16^.

Detailed molecular profiling of tumor tissue can potentially enhance both diagnostic and therapeutic strategies. However, metabolomic insights are not obtained with conventional techniques for skin cancer treatment. In particular, the field of lipidomics has gained attention during the past decade due to technological advances facilitating unprecedented detail in the analysis of lipids. Lipidomics has solidified its role within oncology, by demonstrating that lipids, in addition to their structural role in cellular membranes, also perform critical functions in cellular signaling^10,17^. The lipidomic profile of a cell is consequently highly characteristic of the cell type, enabling the use of lipidomics for fingerprinting of tumor cells. This capability offers potential for the development of highly accurate diagnostic techniques. Mass spectrometry (MS) is one technique widely adopted for lipidomics owing to its capacity for highly sensitive and accurate analysis. The abundance of an ionized compound is detected according to its mass-to-charge ratio (m/z). Techniques for spatially resolved MS, known as MS imaging (MSI), enable the generation of hundreds of compound-specific images from a single experimental run, providing comprehensive metabolomic mapping. A mass spectrum with a spatial component is conventionally referred to as a *pixel*. The evolution and refinement of MSI over recent decades has unveiled new possibilities for clinical applications. Its capacity to render detailed visualizations of metabolites and proteins has not only increased its value in the realm of molecular histology but also highlighted a potential for ambient MS techniques as real-time analytical tools during critical procedures such as cancer diagnosis and surgery^18^. Matrix-assisted laser desorption/ionization mass spectrometry imaging (MALDI-MSI) is a widely implemented technique enabling high spatial resolution. A key benefit of MALDI is its soft ionization process, which preserves the molecular profile of the sample. This facilitates detailed molecular histology with predominantly singly charged, unfragmented molecules represented. However, MALDI-MSI datasets are large and complex, making data-analysis resource intensive.

The use of MS for recognition and delineation of solid tumors presents a classification problem that can be addressed with machine learning (ML). In particular, supervised learning, a subfield of ML, has potential in this context, as it leverages pre-labeled data to predict classifications for new, unseen data. One notable algorithm employed for supervised ML is logistic regression (LR). LR essentially models the probability (P) that a given observation (Y) belongs to a particular class, which in the domain of tumor detection, is typically binary: either *tumor* (1) or *not tumor* (0). The estimated probability of Y = 1 is denoted *P*(*Y* = 1) *and* is determined by a linear combination of the predictor variables, such as *m/z* values in MS data. The coefficients for the model can essentially be interpreted as a specific *m/z* values importance for the classification outcome. A threshold, typically set at 0.5, determines the classification: if the predicted probability is greater than this threshold, the data point is classified as Y = 1 (e.g. *Tumor*). Conversely, if the probability is below the threshold, the data point is classified as Y = 0 (e.g. *Not tumor*). LR is particularly useful when the relationship between the predictor variables and the probability of class membership is non-linear. This is likely the case in the complex task of recognizing tumor tissue from MS data.

We aim at demonstrating the utility of MALDI-MSI in combination with LR for SCC tumor delineation, while providing metabolomic characterization that could enhance our understanding of SCC pathophysiology. Our research has the potential to establish a role for MS techniques in the therapeutic management of SCC, offering a significant advancement in addressing the current and prospective challenges in the treatment of this condition.

## Results

**Figure 1:**
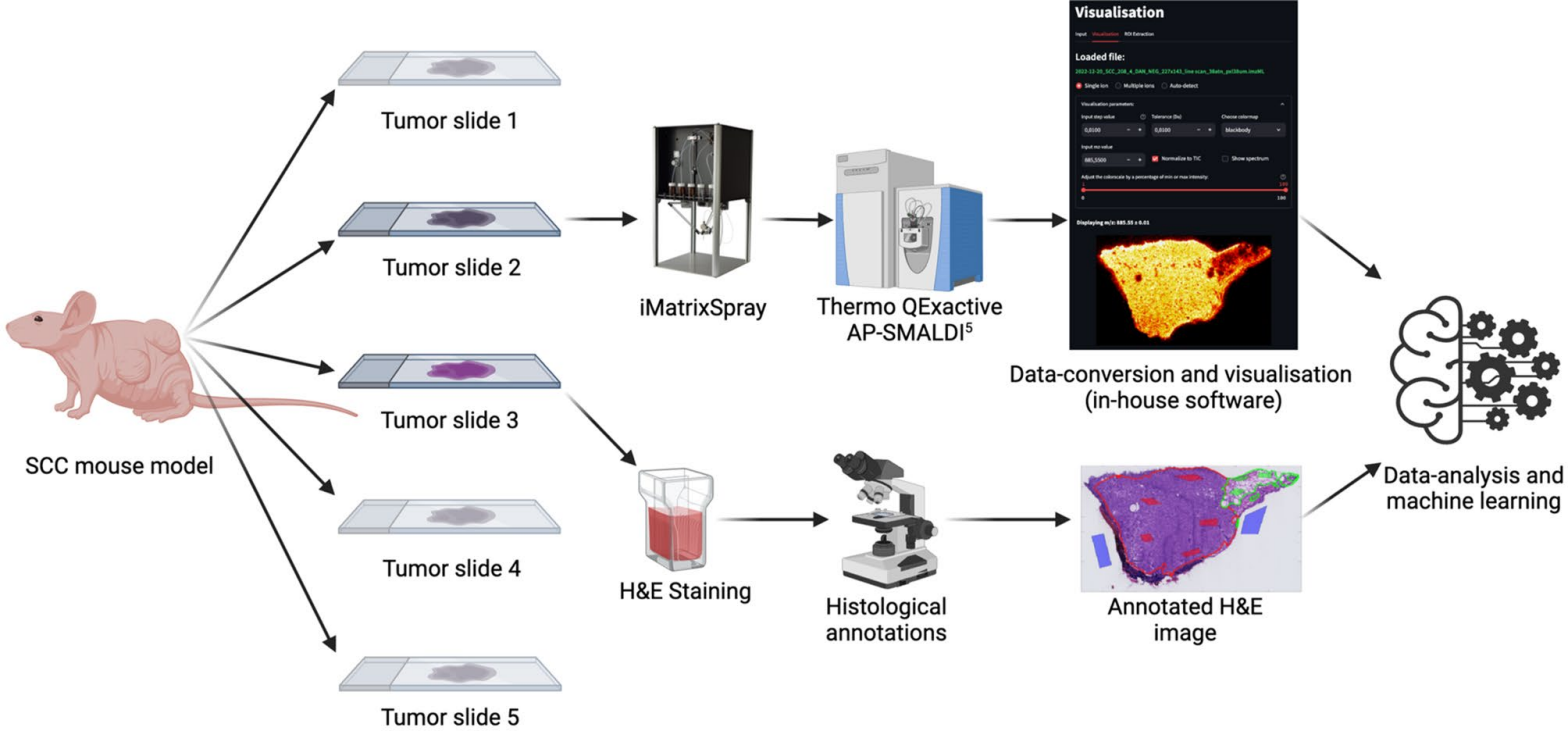
Workflow for sample preparation and data collection. Illustrates the processes for both modalities: MALDI-MSI and histopathology. Source of vector graphics: www.BioRender.com.

**Figure 2:**
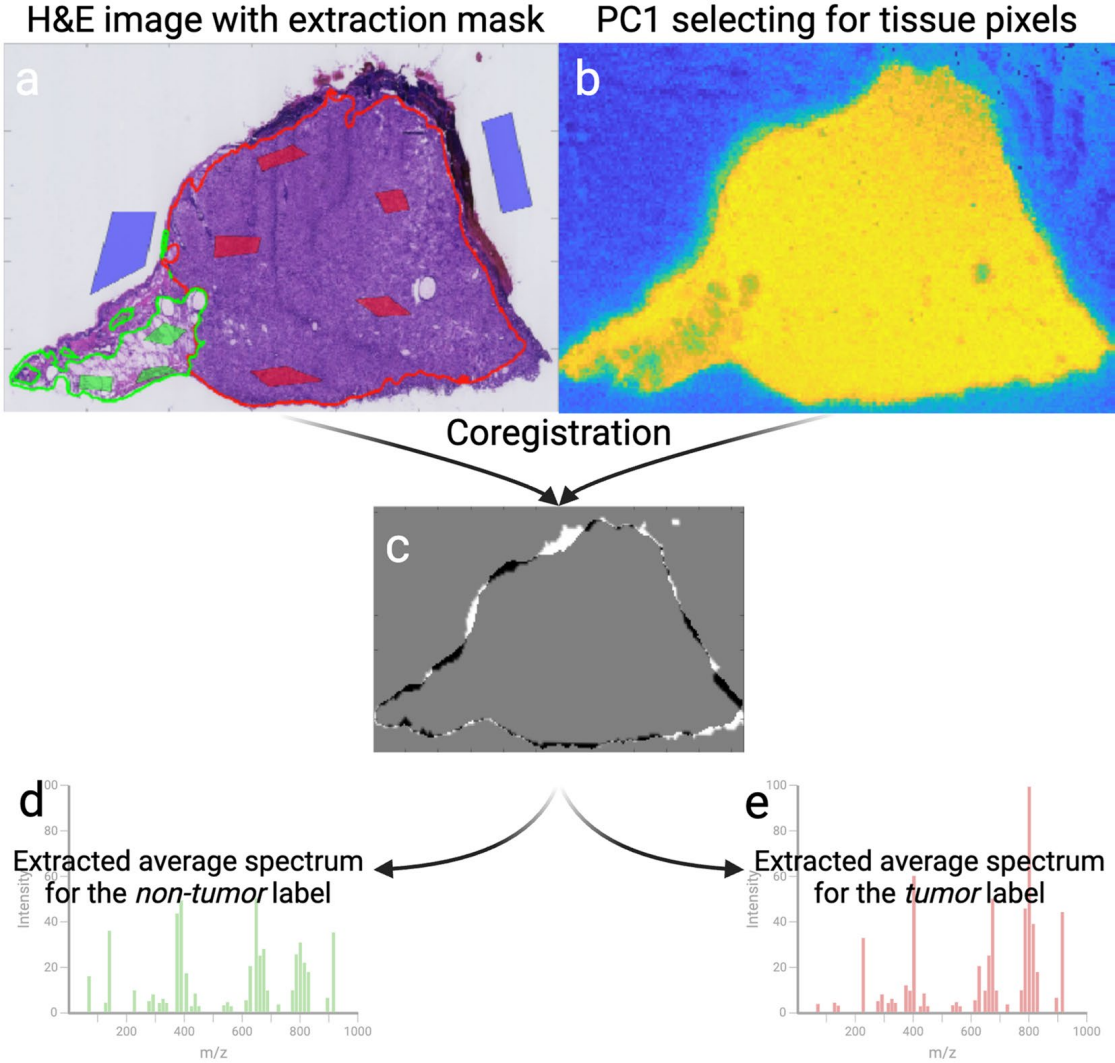
Coregistration and data-extraction. (**a**) Example of an H&E image with annotations drawn in freehand and the extraction mask, drawn as colored shapes. Data extracted from the green areas of the extraction mask is labelled as *Non-tumor*, data from the red areas as *Tumor* and data from the blue areas as *Background*. (**b**) Projection of the first principal component of the MSI data (PC1) for outlining the tissue. (**c**) Coregistration of the tissue area. Grey signifying pixels that are present in both images, white signifying pixels exclusively present in the H&E-image mask and black indicating pixels exclusively present in the MALDI image. (**d,e**) Illustration of the principle of extracting and labelling the spectra according to the extraction mask. A spectrum for pixels labelled as background is not shown for simplicity.

A total 25 datasets, each comprising histological and MSI data from adjacent mouse SCC tissue sections, were collected. Figure 1 shows the workflow from tumor excision to initial data-processing. All 25 samples contained viable tumor in various stages as well as at least one other tissue class (described in “Materials and methods” section). Final labelling of the MSI pixels were grouped to either *Non-tumor* or *Tumor* and all 25 tumors were confirmed as SCC by histological evaluation from the annotating pathologist. The annotating pathologist left a substantial number of pixels unannotated in the image to ensure an adequate amount of data for model validation purposes. Figure 2a shows an example of an annotated H&E image with the extraction mask used for labelling of the MSI pixels.

### Data quality and processing

**Figure 3:**
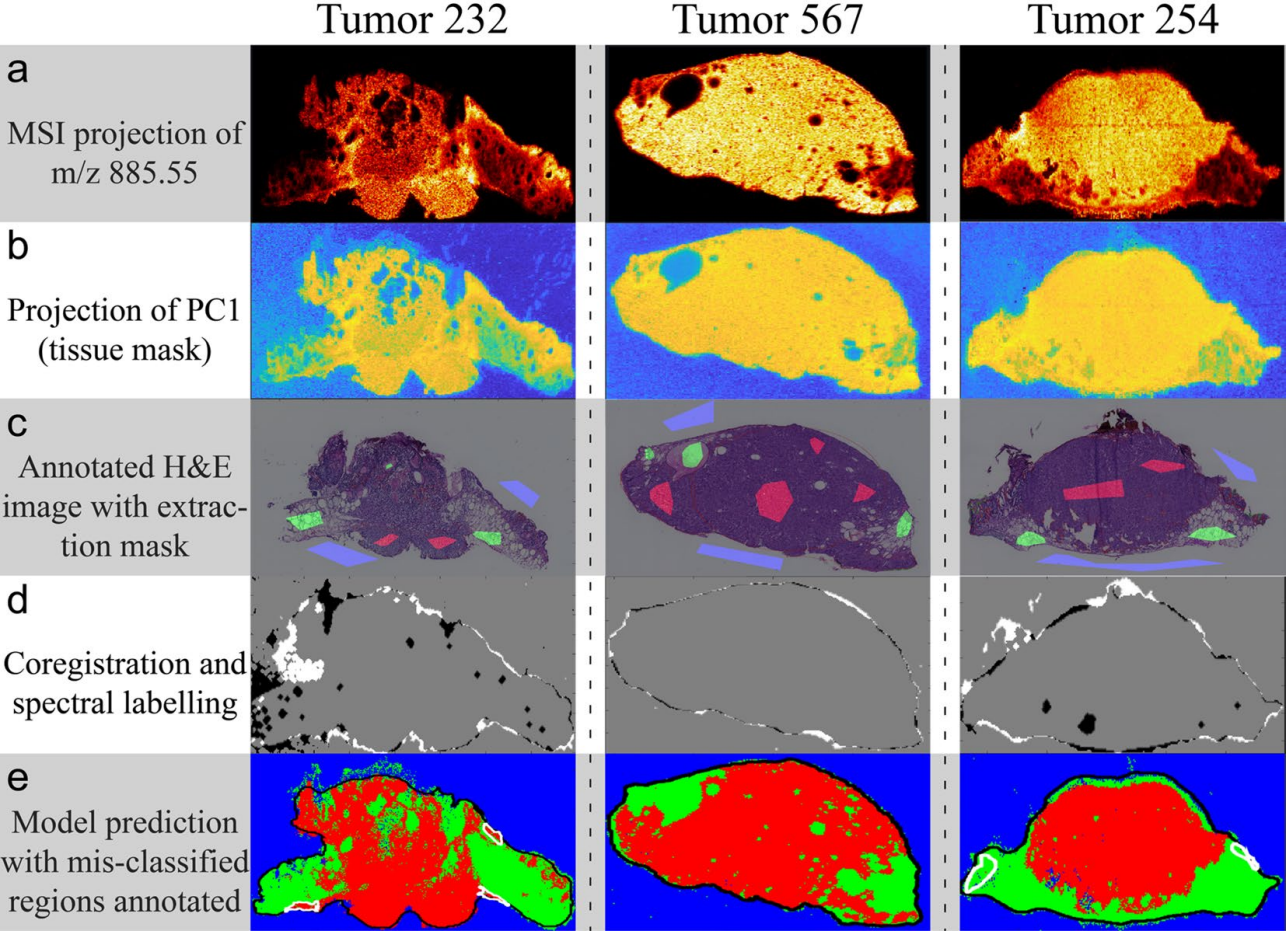
Data-analysis workflow with intermediary images. Demonstrates the data analysis workflow through representative datasets. (**a**) MSI of the ion present at m/z 885.55. (**b**) PC1 projection used for coregistration. (**c**) H&E staining with annotations and extraction mask. Red: Tumor, Green: Non-tumor, Blue: Background. (**d**) Coregistration of H&E image and PC1. Grey: pixels found in both images, Black: pixels exclusively found in MSI, White: pixels exclusively found in H&E image. (**e**) Projection of model classification. Red: Tumor; Green: Non-tumor; Black: Tissue boundary; White: Misclassifications according to the validating pathologist with Tumor 232 showing an example of false positive prediction and Tumor 254 showing an example of false negative prediction.

The quality of each unprocessed MSI dataset was assessed by visualizing the molecular distribution of a metabolite known to be present across the tissue (Fig. 3a). All datasets were of satisfactory quality and spectral recalibration and peak picking were performed. A total of 966 peaks were used for further analysis. A plot of the picked peaks is available in the supplementary material (Fig. SM2). After processing, the first principal component was visualized and used as the foundation for the coregistration of the tissue area to the annotated H&E image (Fig. 3b–d). The labelled data from the 25 datasets was used to train the LR model to classify MSI pixels according to the labels (i.e. *Background*, *Tumor* or *Non-tumor*). The classification of background was included for the purpose of visualizing the predictions.

### Predictive accuracy

**Figure 4:**
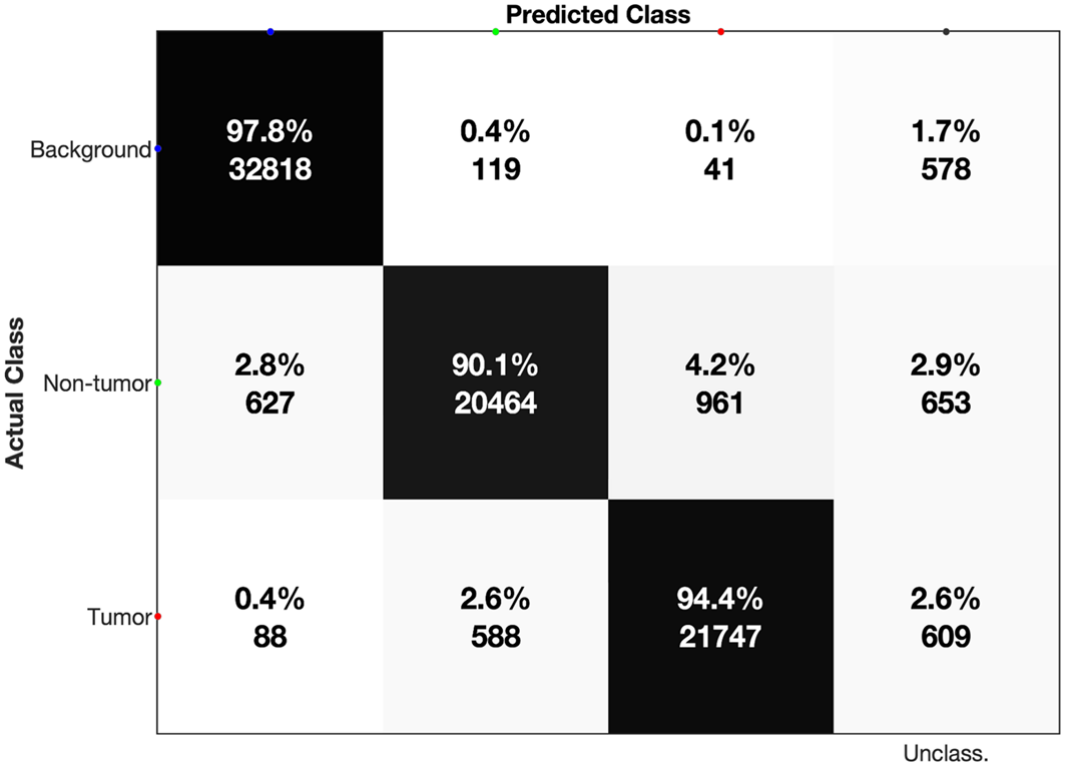
Leave-one-group-out cross-validation. Cross-validation conducted on a leave-one-group-out basis with 25 iterations (each dataset left out in one iteration of training). Percentages show the proportion of pixels that fall into each category summed across each iteration. Numbers show the absolute number of pixels within each category. Unclassified pixels are those which failed to achieve a scaled probability > 0.5. Details about the validation method are provided in “Materials and methods” section.

A total of 79.293 pixels across the 25 datasets received a label based on the extraction masks. The total number of pixels labelled as tissue (either *Tumor* or *Non-tumor*) was 45.737. The leave-one-group-out cross validation (described in the “Materials and methods” section) indicates a model sensitivity of 94.4% (i.e., the proportion of tissue pixels correctly classified as *Tumor*), and a specificity of 90.1% (i.e., the proportion of tissue pixels correctly classified as *Non-tumor*) for the labelled data (Fig. 4). The predictive accuracy of the model on labelled data within the tissue was 92.3% (calculated as the ratio between correctly classified tissue pixels to the total number of tissue pixels). For the calculation of sensitivity, specificity and accuracy the reader is referred to the “Materials and methods” section.

### Predictive power

**Figure 5:**
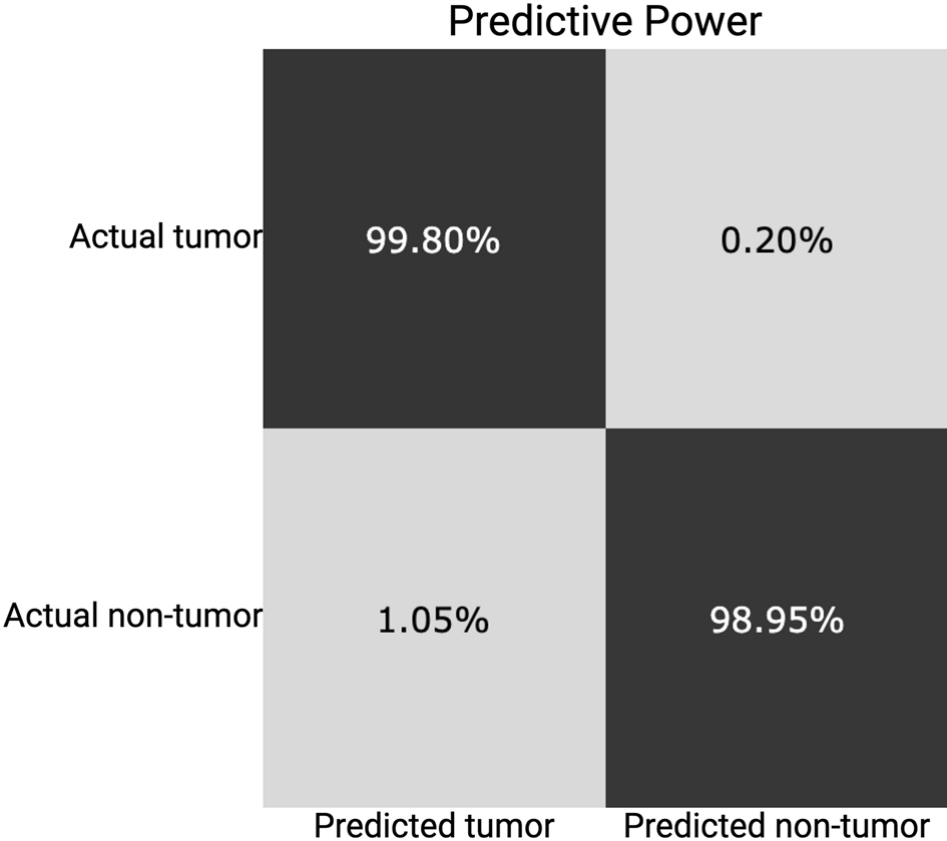
Confusion matrix for model evaluation. Confusion matrix based on the areas of misclassification for evaluation of the model prediction. Percentages represent the total area of a classification type relative to the total tissue area.

In Fig. 3e the prediction for three distinct tissue sections is shown along with areas of misclassification as annotated by the validating pathologist. The summed areas of false positive predictions and false negative predictions were compared to the total tissue area. Figure 5 shows the confusion matrix with the relative areas in percentage. The validation showed a combined model accuracy of > 99% with a sensitivity of 99.8%. False positives consisted primarily of panniculus muscle tissue, misclassified as tumor tissue. Common for both false negative and false positive classifications were that the areas were located on the edge of the tissue, indicating that improvements of the coregistration could yield better classification results. One solution could be to have more stringent criteria for accepting the coregistration by introducing a threshold for the number of mis-matched pixels. Predictions for all datasets with mis-classified areas annotated and corresponding H&E images with annotations are available in the supplementary material (Attachment SM1). We did not observe any areas of discrepancy between the annotations of the annotating pathologist and the validating pathologist.

### Spectral characteristics

**Figure 6:**
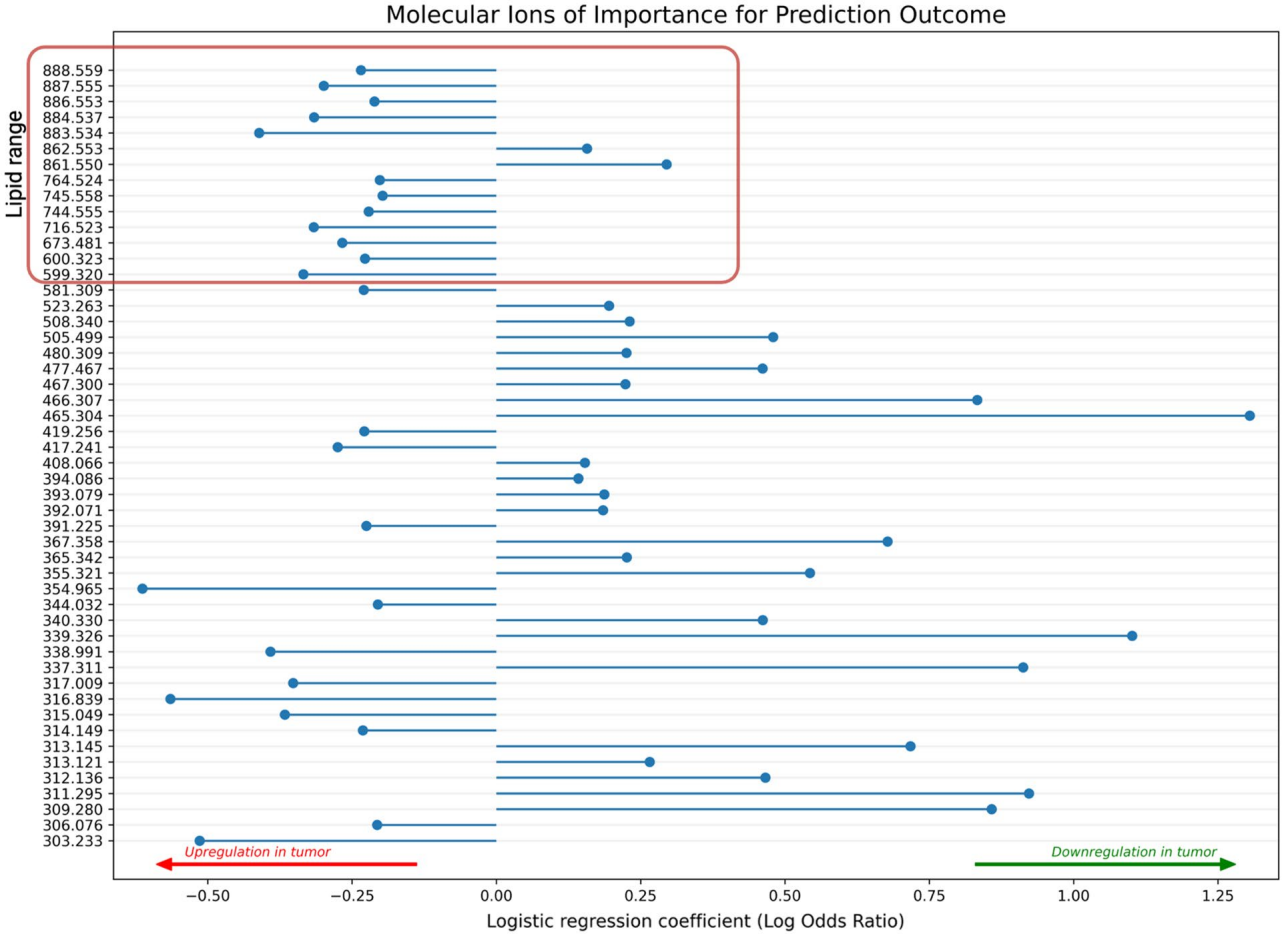
Molecular ions of importance for prediction outcome. Plot displaying the 50 ions of largest importance for the classification outcome as specified by the coefficients of the logistic regression model. Negative coefficients suggest upregulation in the tumor regions and positive coefficients suggest higher intensity in the non-tumor regions (i.e. downregulation in the tumor regions). The coefficients are sorted according to m/z value and the lipid-range is highlighted by the red box.

The coefficients for the trained model, signifying the importance of each of the 966 m*/z* values in the classification were extracted and plotted. LR coefficients for the 50 m*/z* values most important for classification outcome are shown in Fig. 6. The plot showing the coefficients of all *m/z* values is supplied in the supplementary material (Fig. SM4). Negative coefficients suggest upregulation in the tumor regions and vice versa. Particularly, ions in the lipid range (defined here as *m/z* 600–900) exhibited upregulation in tumor areas, as depicted in Fig. 6. The ion at *m/z* 465.304, tentatively annotated as cholesterol sulphate, showed the largest downregulation in tumor regions when compared to the *non-tumor* regions (Fig. 6). The best predictor in the lipid range was the ion observed at 883.534, tentatively annotated as phosphatidylinositol (38:5). Examples of the distribution of these ions, visualized by the unprocessed MSI data are available in the supplementary material (Fig. SM5). For further inspection of specific ion distributions the reader is referred to the Metaspace annotation platform according to the Data availability statement below.

## Discussion

In the present study, we focused on the applicability of a combination of MALDI-MSI and ML for SCC characterization and margin detection. The LR model demonstrated a high level of predictive accuracy and predictive power, affirming the utility of MALDI-MSI in combination with LR in SCC characterization. The method outlined facilitates a deeper, metabolomic understanding of the pathophysiological mechanisms behind SCC and can potentially be a more comprehensive and accurate diagnostic method than traditional histology. However, the method is primarily a tool for characterization of tumor tissue, and not intended to replace traditional histological approaches in its current form. Traditional histology has, despite shortcomings such as interobserver variation, a well-established role in the clinical diagnosis of SCC. Nonetheless, our method showcase how MS data can be effectively employed in the accurate diagnosis and delineation of SCC. A study by Janßen et al. reported a level of accuracy similar to our findings on human samples of non-small cell lung cancers (including adeno-carcinoma and SCC). They report accuracies of 99.0% and 98.3% for a neural network and a linear discriminant analysis, respectively, trained for classifications based on MALDI-MSI spectra obtained in the mass range *m/z* 700–2700^19^.

While the potential of MALDI-MSI in SCC characterization is recognized, our study, focusing specifically on mouse SCC tissue and integrating LR, substantiates and extends this potential by investigating a previously unexplored combination of methods and models^20^. The *m/z*-range chosen for data acquisition (*m/z* 300–1000) encompasses lipids and small metabolites, both of which are hypothesized to be altered during tumorigenesis^10,17^. A potential use for this information is the application of ambient MS techniques coupled with surgical tools, for real-time metabolomics during surgery. This would likely require the use of a mass range similar to the one used in this study, since energy-based surgical techniques would not conserve the protein structure under ambient conditions. This could effectively combine diagnosis, delineation, and tumor removal into one^21^.

To discern the differences in specific ions between tumor and non-tumor tissues, we inspected the coefficients of the trained LR model. Our findings confirm a general upregulation of lipids in the tumor area (Fig. 6, Fig. SM4). A smaller study on oral SCC found that two phosphatidylcholines, namely phosphatidylcholine (16:0/16:1) and phosphatidylcholine(18:1/20:4) present at *m/z* 770.5 and *m/z* 846.6, respectively in positive mode, provided good tumor delineation^22^. In Fig. SM5, we show the distribution of two of the most important metabolites for the prediction. We find that these metabolites do to some extend differentiate between tumor and non-tumor regions, however neither metabolite provide a sufficient binary distinction for accurate tumor delineation. In our study we conclude that focusing on single ions may provide valuable insights into tumor pathology, however, for accurate delineation of the tumor, a multivariate approach is needed.

We used negative mode with 1,5-diaminonaphthalene as the MALDI matrix to efficiently ionize a broad range of lipids, fatty acids and other small metabolites that tend to ionize better in negative mode^23,24^. Studies on the use of MALDI-MSI for characterization of other cancers such as breast, colon, liver, and ovarian cancer have shown promising results by investigating higher mass-ranges for proteomics^11,25–27^. The field of proteomics does, however, lend itself less useful in the aim of implementing MS in the clinical, ambient settings due to the sample preparation required to conserve the primary protein structure.

The final evaluation by the validating pathologist suggests a very high concordance between classic histology and our model prediction. Notably, the validation shows a sensitivity of 99.8% (Fig. 5). This is important as model sensitivity is crucial in cancer diagnostics; tumor cells that are undetected and associated with a false sense of security could constitute a significant risk for the patient.

Traditional histopathological evaluations face challenges such as inter-observer variation, underscoring the importance of more consistent and objective diagnostic tools^15,28^. Molecular histology techniques like immunohistochemistry (IHC) and fluorescence in situ hybridization (FISH) provide a more decisive tumor identification than classical histology but are specific to certain antigens or genomic sequences. MS based techniques provide rich metabolomic information that, in the realm of SCC diagnosis, could provide new prognostic markers and facilitate the identification of novel tumor subgroups, aiding in the stratification of tumors. This could direct targeted treatment strategies based on the molecular profile of the tumor^20,29^. For instance, it may reveal metabolic differences in SCC between patients with normal immune function and those who are immunosuppressed, offering deeper insights into tumor behavior.

### Challenges and limitations

This study utilized murine samples rather than human samples. While larger biological variation can be expected in human samples, increasing complexity in the data, this study does provide important foundational insights and a framework for general tumor characterization using MS data. The specificity of the model presents certain challenges and limitations. It was not designed to differentiate between different tumor grades, which means it might not encapsulate the full spectrum of SCC variations. Furthermore, should a tissue type, which was not represented in the training data emerge in subsequent samples, our model was not trained for classification of this tissue type. The primary focus of our model was on viable tumor cells, leading to the categorization of necrotic tissues as *non-tumor*, however the model can be retrained and tailored to the specific needs for future classification challenges encompassing several different tissue types. Another limitation of this approach is that evaluating on a pixel-by-pixel basis does not incorporate the spatial, relational information that is important in classical histology, where aspects such as depth of invasion and perineural invasion are important prognostic markers^30,31^. A more sophisticated machine learning model might address this by attributing a weight to each pixel based on the classification of surrounding pixels, thus modifying the classification threshold. We did not test the LR model against other ML classification models, as we achieved both high accuracy and interpretability with this model. While other ML algorithms, including random forests, support vector machines, and neural networks, have been effectively utilized for tumor classification using MALDI-MSI data^32–34^, the significance of interpretability remains crucial, especially in the context of biomarker discovery^35^.

Tuning of model parameters and the inclusion of more data from a diverse set of samples may lead to even higher accuracy and better coverage. For good model training, it is crucial that the extracted spectra are labelled correctly. The use of adjacent sections for MSI and histological annotation introduces a risk of misalignment of the sections, which could in turn result in erroneous labelling in the coregistration process. Some misalignments in the edges of the tissues can be observed for the examples in Fig. 3d. Using the same tissue section for both the histological evaluation and the MALDI-MSI experiments would eliminate this risk and has proven feasible^20^. However, practical considerations in this study led to the decision to use adjacent sections.

The choice of leave-one-group-out cross-validation in our study was made to mitigate the risk of overfitting at both the pixel and tissue levels, ensuring the model’s reliability and effectiveness when encountering new tissue samples. This consideration is crucial for maintaining a clinical perspective, as it aligns with real-world scenarios where the model must accurately interpret tissues it has not previously analyzed. Although k-fold cross-validation is a more commonly utilized method in ML for its general applicability, it presents a significant risk of overfitting to specific tissues in this context. This is because k-fold cross-validation typically involves leaving out only single annotations or pixels in each iteration, rather than excluding entire datasets.

Since our leave-one-group-out cross-validation involves testing the model on complete, separate datasets rather than a mixed set of data points, it limits the ability to generate a meaningful standard deviation. While this approach introduces a challenge in calculating the standard deviation for the model performance across different folds, this limitation does not significantly detract from the model’s overall reliability and applicability.

The validation phase of this study incorporated a side-by-side comparison of the projection of the LR predictions to the corresponding H&E image (Supplementary Material, Attachment SM1). This approach could potentially introduce a bias to the validation process, as the validating pathologist will become familiar with the performance of the model. An ideal approach would involve complete annotation of the H&E-image and only subsequently comparing the annotations with the model projection. Furthermore, comparison of our results to a third technique such as IHC or FISH applied to the same tissue sections could help identify shortcomings in the model and combined with histology, provide a conclusive measure of model accuracy. While the results presented in this study suggest an accurate method for tumor delineation, it does not provide the high spatial (optical) resolution achievable with classical histology.

### Future perspectives

Common for all the techniques mentioned (MALDI, H&E, IHC and FISH), is the need for tissue excision and sectioning followed by specialized sample preparation. As we look towards broader clinical applications, techniques that do not require these preliminary steps would be highly valuable. Ambient MS techniques, such as rapid evaporative ionization mass spectrometry (REIMS), have the potential to be used for imaging as well as surgical and real-time diagnostic purposes^21^. Spectral differences between MALDI data and REIMS data are yet to be assessed, however, the recent interest in the field of MSI to characterize and compare various modalities is noteworthy. This could potentially lead to a scenario where data from one modality can provide useful information for optimization of another modality^36,37^.

While our study targeted SCC in mice, there is inherent potential to extend this approach to other cancer types and to human samples. This adaptability could foster the development of a comprehensive diagnostic tool with the capability of discerning various malignancies accurately at early stages, promoting early, tumor-specific interventions and improved patient outcomes^38^.

### Conclusion

The global increase in KC incidence rate necessitates innovative and more effective methodologies for diagnosis and tumor characterization. Our method, combining MALDI-MSI and ML, offers both objectivity and tissue specificity, showing sensitive recognition and accurate delineation of SCC tumor tissue. We achieved a predictive of power of > 99%, indicates a potential complimentary role for MS-based techniques to conventional therapeutic approaches.

In conclusion, the results presented in this study confirm that a LR model trained on MALDI-MSI data can accurately classify and delineate SCC in a mouse model. While our research has unveiled certain challenges and limitations, it lays a robust foundation for future research aiming at the implementation of metabolomics in routine cancer diagnostics.

## Materials and methods

### Mouse model

Hairless mice (C3.Cg-Hr^hr^/TifBomTac, Taconic, Ry, Denmark) were irradiated by ultraviolet radiation three times per week as described by Lerche et al. Tumors were excised from mice that developed SCC (n = 25) and embedded in a mixture of hydroxypropyl methylcellulose and polyvinylpyrrolidone (7.5%:2.5%)^39^. Embedded tumors were marked and stored at – 80 °C until further sample preparation.

Approval of the study was granted from the Danish Animal Inspectorate (# 2019‐15‐0201‐01666) and all experiments complied with Federation of European Laboratory Animal Science Associations guidelines for animal experimentation. Additional details regarding the mouse model can be accessed through previously published work by Lerche et al.^40^. One section from each tumor was hematoxylin and eosin (H&E) stained and one section was analyzed with MALDI-MSI, resulting in 25 datasets each comprising of one H&E image and one MALDI-MSI image. Reporting of the study results are in accordance with the ARRIVE guidelines (Animal Research: Reporting of In Vivo Experiments)^41^.

### Histopathology

Excised tumors were cryosectioned to five 10 µm sections on microscope glass slides (Fig. 1; Dako Denmark, Glostrup, Denmark). Tissue images of selected tumors are available in the supplementary material (Fig. SM1). The central slide from each tumor was stained with a procedure for H&E staining, described in detail in the supplementary material (Attachment SM2). This slide was digitally scanned at a magnification of × 40. A histopathological report was generated by a pathologist with detailed annotations of various tissue-classes using the open-source software, QuPath (Version 0.3.2, available from: https://qupath.github.io/). This pathologist is referred to as the *annotating pathologist* throughout this study. The remaining slides were stored at − 80 °C until MALDI-MSI analysis. Annotated classes included “SCC” (covering all grades), “Keratosis”, “Keratin pearls”, “Inflammatory cells”, “Inflammatory/necrotic area”, “Panniculus muscle”, “Glands” and “Peri-tumorous area with dilation/hyperplasia of glands (vacuoles/cysts)”. Histological annotations were used to guide the labelling and extraction of MS data from the MSI datasets.

### MALDI-MSI

MALDI-MSI was performed on tissue sections from a total of 25 excised tumors. One tissue-slide adjacent to the H&E-stained section was selected and inspected for artifacts. Upon satisfactory quality, the section was placed in a vacuum desiccator for 10 min. Immediately after desiccation, the slide was sprayed with a solution of 1,5-diaminonaphthalene (3.3 mg/mL in 90% MeOH), using an iMatrixSpray^42^. The sprayer was set at a height of 80 mm, line distance of 1 mm, speed at 90 mm/s, density of 3 µL/cm^2^, 12 cycles and an area of 40 × 40 mm. After assessment of the crystal formation, MALDI-MSI data was acquired in negative ion mode on an AP-SMALDI^5^ ion source (TransMIT GmbH, Giessen, Germany) attached to a Thermo QExactive Orbitrap mass spectrometer (Thermo Scientific, Bremen, Germany). The spectral resolution was set to 140.000 with an *m/z*-range of 300–1000 and a pixel size of 40–50 µm.

In-house software written in Python 3.9 and MATLAB was used for data-conversion and quality control. The data was converted from the Thermo raw data to the open MSI data format imzML. The imzML format is widely used and allows for easy visualization of MSI data across various instrument vendors and software platforms. Images in which the tissue area was not clearly differentiated from the background were discarded and reacquired using a new tissue section.

### Data-analysis and model training

The main data-analysis workflow was built using MATLAB (R2021b, available from https://www.mathworks.com/). The workflow relied on the MATLAB toolboxes, Bioinformatics, Curve Fitting, Signal Processing, and Statistics and Machine Learning. Preprocessing of the MALDI-MSI data included *m/z*-recalibration based on two reference ions (*m/z* 311.2950 and 885.5493) with a tolerance of 30 ppm. A linear surface fit was applied to account for calibration peaks missing in the spectra for some pixels. Spectrum averaging from tissue-pixels was done based on presence of signal at *m/z* = 885.5493, which is expected to be present in all tissue-pixels. Peak picking was performed on the mean spectrum, smoothed using 2nd order Savitzky-Golay filtering with a 21-point Gaussian-weighted window. These procedures were implemented to guarantee spectral precision and minimize noise. PCA components 1–3 were projected to assess the data-quality after processing and to generate a tissue mask to be used for accurate labelling of pixels during coregistration.

An image extraction mask was created based on the histopathological annotations. This mask was used for subsequent extraction and labelling of the corresponding MALDI-MSI data (Fig. 2). This approach allows for accurate labelling of pixels while preserving a large number of unlabeled pixels for model testing. Accurate labelling was attained by resizing, warping, and scaling the annotated H&E-image and performing a coregistration, optimizing for the best overlap of tissue-pixels in the two images. The extraction-mask was transformed identically. The interface of this step is shown in supplementary material (Fig. SM3). The coregistration step was concluded by extraction and labelling of the MSI pixels.

LR models were trained based on the extracted, labelled data. The various models were trained for three distinct classification problems: *Tumor* vs. *Non-tumor* + *Background*, *Tumor* + *Non-tumor* vs. *Background*, and *Non-tumor* vs. *Tumor* + *Background*. The training data was normalized sequentially by log10 transformation and scaled to the Euclidean sum of each spectrum. Predictions from the model were rendered into an image that matched the dimensions of the original MALDI-MSI data. We employed a *leave-one-group-out* cross-validation approach on the sample level to evaluate the model: in each iteration, one dataset, consisting of all annotated regions within a tumor-tissue sample, was withheld from model-training and then used for validation. The model classified an MSI pixel based on a scaled probability threshold. Specifically, a MSI pixel was assigned to a particular label if its scaled probability for the class exceeded 0.5. If the datapoint did not have a scaled probability of > 0.5 for any class, the pixel remained unclassified (Fig. 4). We used the term *predictive accuracy* to describe performance of a model on labelled datapoints. A series of LR models were trained to classify spectra according to the provided labels. Each pixel (representing one mass spectrum) in a given MALDI-MSI underwent classification by its probability of belonging to one of the three classes: *Tumor*, *Non-tumor* or *Background*.

The coefficients of the trained LR model, specifying a given *m/z* value’s relative importance for the classification outcome, were plotted to show which ions were up- or downregulated in the tumor regions (Fig. SM4). Specific molecular ions were tentatively annotated using the Metaspace MSI data annotation platform (https://metaspace2020.eu/), utilizing the LIPID MAPS® database (https://www.lipidmaps.org/)^43,44^. Metabolite annotations were matched within a 3 ppm tolerance on the exact mass of each molecule. Hence, the annotations were tentative and not confirmed by tandem MS. The full list of metabolite annotations is available through the Metaspace platform as described in the Data availability statement.

### Evaluation of model predictions

A second pathologist with extensive experience in murine skin cancers, unacquainted with the dataset and methods of analysis, was consulted for evaluation of the model predictions. We refer to this pathologist as the *validating pathologist* and use the term *predictive power* to describe the performance of a model on unlabeled data. The predictive power was assessed by annotation of mis-classified pixels within the tissue area. The validating pathologist was instructed to compare the original H&E-stained images with the images produced by the trained ML model. In a side-by-side comparison, the validating pathologist marked any incorrect representations in the model projection (Attachment SM1). Upon detailed annotation of incorrectly classified pixels the proportion of correctly classified pixels was calculated within each dataset. This metric was used to assess the overall predictive power of the model.

We use the term* sensitivity* to describe the ability of the model to correctly identify tumor pixels. We calculate sensitivity as follows: $\frac{True \,positives}{True\, positives+False\, negatives}$. We use the term *specificity* to describe the ability of the model to correctly identify non-tumor pixels. The specificity was calculated by $\frac{True\, negatives}{True \,negatives+False \,false\,positives}$. Accuracy, used to assess the general performance of the model, was calculated using the formula: $\frac{True\, positives+True\, negatives}{True\, positives+False \,negatives+True\, negatives+False \,false\, positives}$. The *tumor* class was regarded as a *positive* case and the *non-tumor* class was regarded as a *negative* case.

### Supplementary Information

Supplementary Information 1.

Supplementary Information 2.

## Data Availability

Datasets related to this article can be found at https://metaspace2020.eu/group/UCopenhagen. Metaspace is an online database for annotation and storage of MSI data^44^. The relevant datasets are prefixed with *M*.

## Abbreviations

FISH: Fluorescence in situ hybridization
H&E: Hematoxylin and eosin
IHC: Immunohistochemistry
KC: Keratinocyte carcinoma
LR: Logistic regression
MALDI-MSI: Matrix-assisted laser desorption/ionization mass spectrometry imaging
ML: Machine learning
MS: Mass spectrometry
MSI: Mass spectrometry imaging
m/z: Mass to charge ratio
PC: Principal component
PCA: Principal component analysis
REIMS: Rapid evaporative ionization mass spectrometry
SCC: Squamous cell carcinoma
