# Supplementary material for: Cutaneous squamous cell carcinoma characterized by MALDI mass spectrometry imaging in combination with machine learning
Source: Sci Rep. 2024 May 15;14:11091. doi: 10.1038/s41598-024-62023-0 (PMC11096391; doi:10.1038/s41598-024-62023-0)
Supplement: Supplementary file 1 — Supplementary Information 1. [file 41598_2024_62023_MOESM1_ESM.docx]

Supplementary Material

Cutaneous Squamous Cell Carcinoma Characterized by MALDI Mass Spectrometry Imaging in Combination with Machine Learning

Lauritz F. Brorsen^1, 2*^, James S. McKenzie^3^, Mette F. Tullin^2^, Katja M. S. Bendtsen^4^, Fernanda E. Pinto^1^, Henrik E. Jensen^4^, Merete Haedersdal^1^, Zoltan Takats^3^, Christian Janfelt^2^, Catharina M. Lerche^1,2^

^1^Department of Dermatology and Wound Healing Centre, Copenhagen University Hospital - Bispebjerg and Frederiksberg, 2400 Copenhagen, Denmark.

^2^Department of Pharmacy, University of Copenhagen, 2100 Copenhagen, Denmark

^3^Department of Digestion, Metabolism and Reproduction, Imperial College London, London, U.K.

^4^Department of Veterinary and Animal Sciences, University of Copenhagen, 2100 Copenhagen, Denmark

Corresponding Author Details

Lauritz F. Brorsen

Department of Dermatology, Copenhagen University Hospital, Bispebjerg

Nielsine Nielsens Vej 9, 2400 Copenhagen

+45 2281 1961

[lauritz.brorsen@sund.ku.dk](mailto:lauritz.brorsen@sund.ku.dk)

Figure sm1: Tissue Images


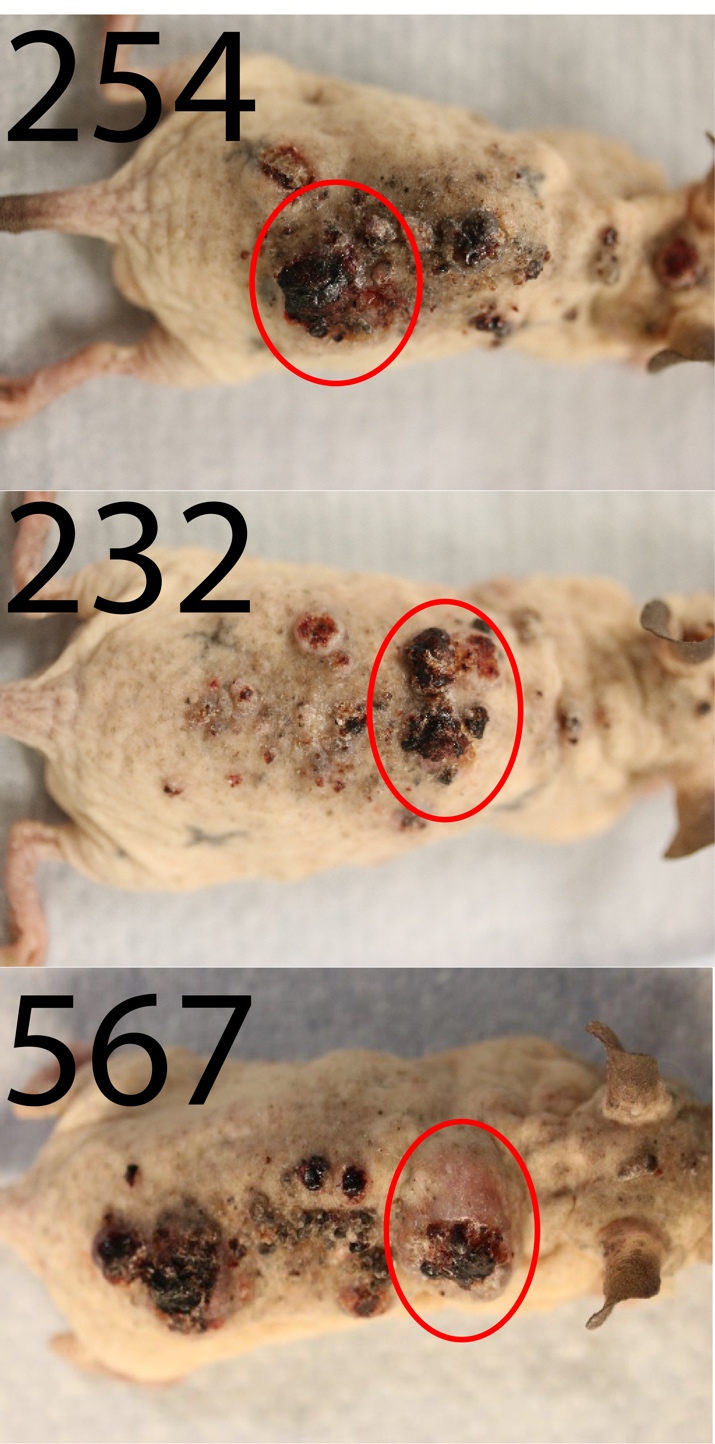


Images of the tumors for three selected mice (Identifiers: 254, 232 and 567) with the excised and analysed tumor outlined by the red circle.

Figure sm2: Peak Picking


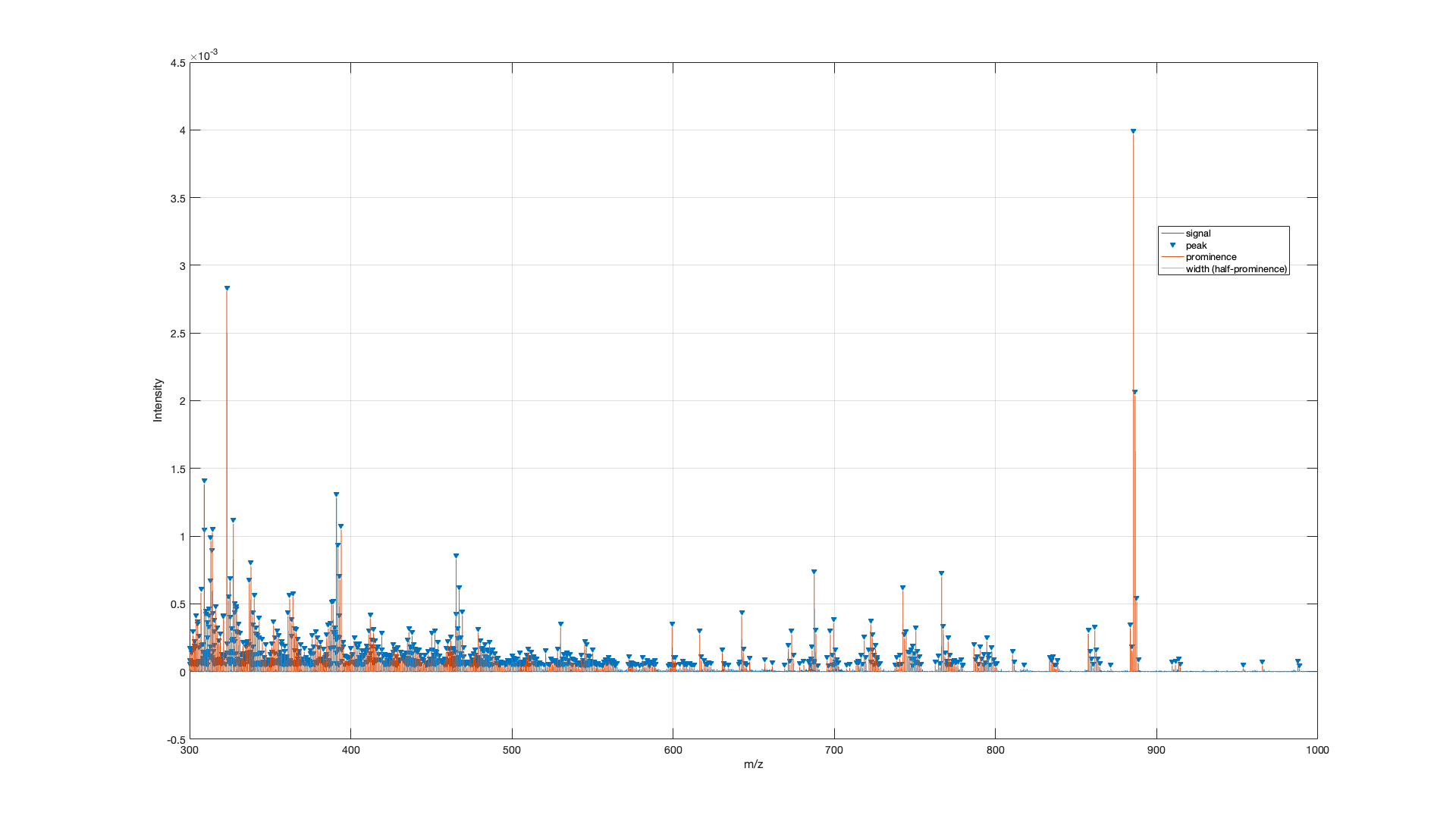


Graph showing the 966 selected recalibrated peaks used for model training and classification across all datasets (n=25), marked by blue triangles.

Figure sm3: Coregistration Interface


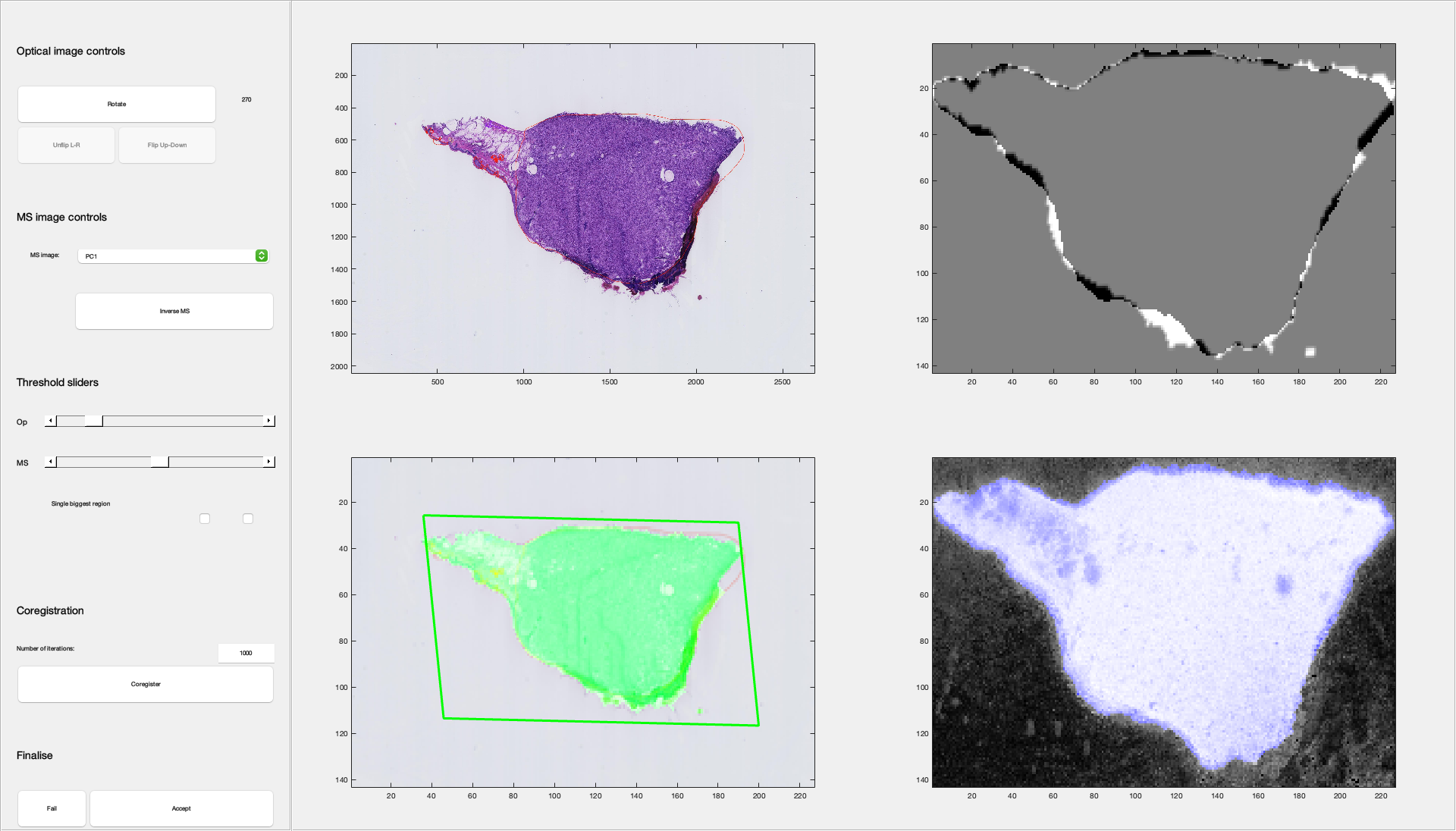


The graphical user interface used for the coregistration process. The control panel to the left is used to define the tissue areas in the H&E image and the MSI respectively and start the automatic coregistration process. Top left image is showing the raw H&E image. Bottom left is showing the downscaled H&E image (to match resolution of the MSI) with the tissue mask in green and the square marking the transformation/warping needed for the tissue to match the tissue area of the MSI. Bottom right is showing the tissue area found in the MSI marked in blue. Top right is showing the coregistration of the tissue areas from the two modalities after image transformation. White pixels are exclusively present in the H&E tissue area and black pixels are exclusively present in the MSI tissue area.

Figure sm4: LR coefficients


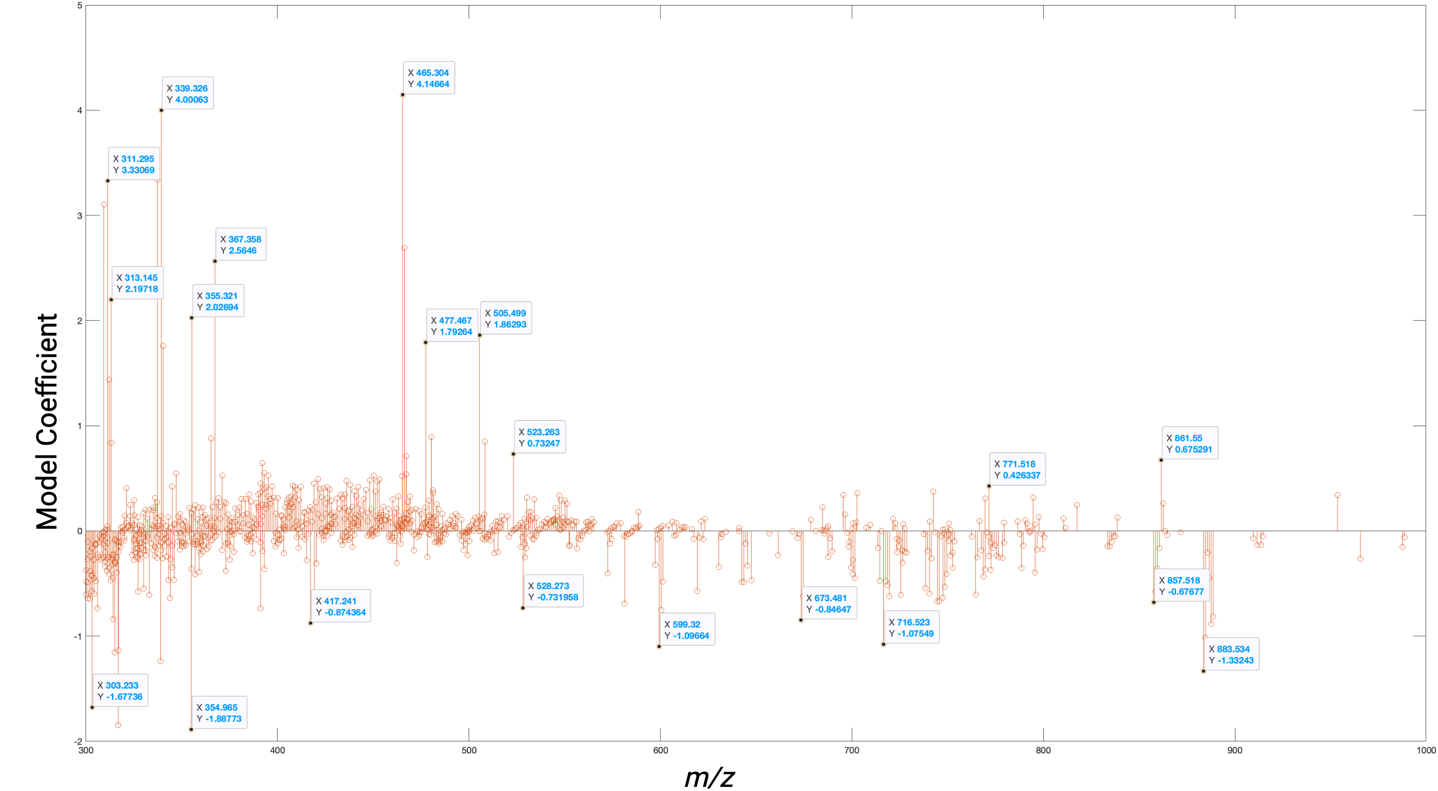


Plot of the coefficients for the trained LR model with some of the most important features labelled.. A positive coefficient indicates that higher intensities of this ion are associated with classification as Non-tumor. Conversely, a m/z with a negative coefficient indicates that higher intensities of the ion are associated with classification as Tumor.

Figure sm5: Metabolite distribution


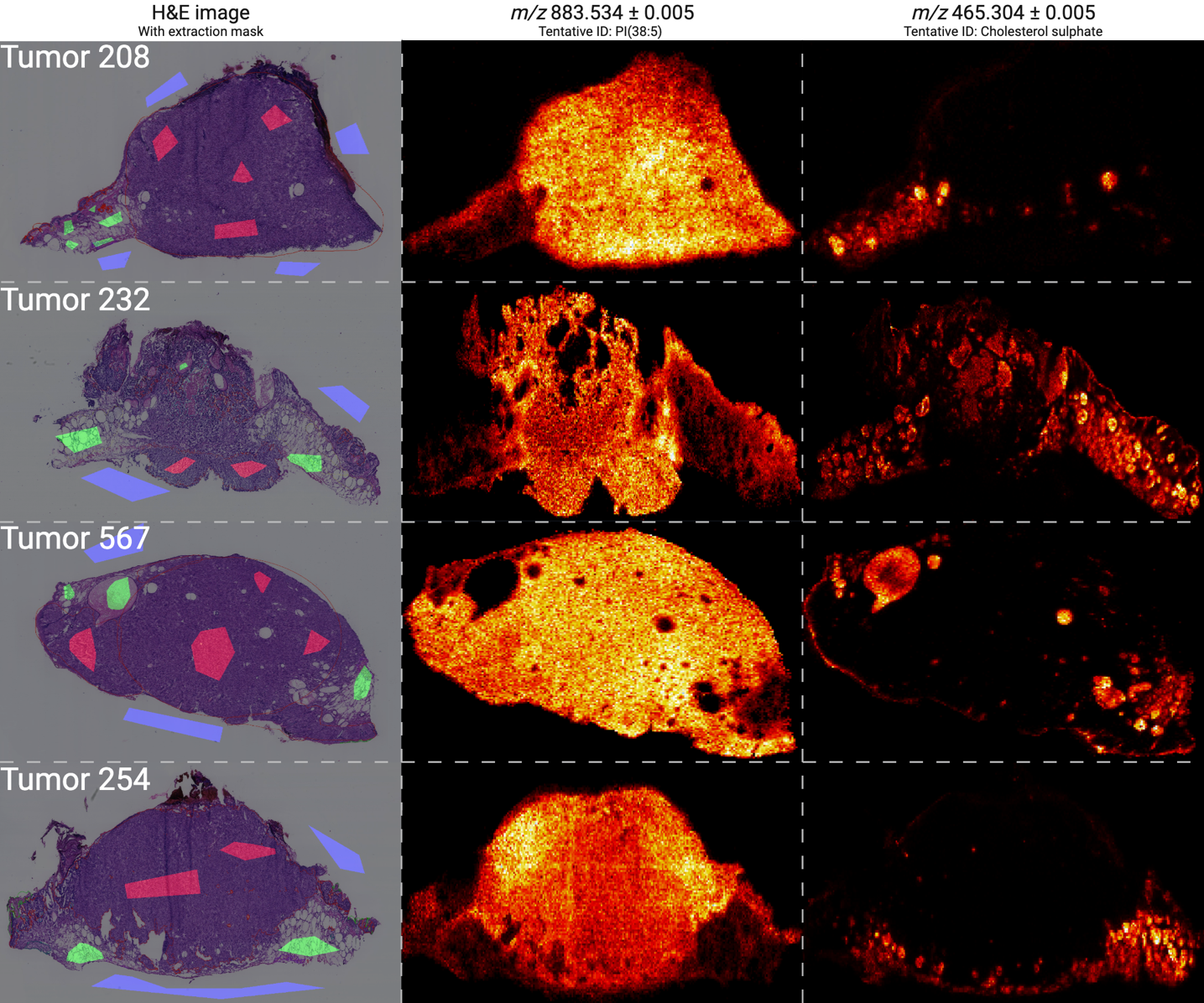
Attachment sm1: Evaluation of Predictive Power

Annotations of error on model projection versus H&E images.

See attached PDF: [SupMat_PredPower.pdf](https://drive.google.com/file/d/1NBzLoOgKaUn858StWIWQUpsYN4YSzR6Q/view?usp=sharing)

Attachment sm2: H&E Staining Protocol

The tissue sections used for histopathology were stained with H&E in a Leica ST4020 Small Linear Stainer, according to the following protocol:

1. 14 s submerged in water
2. 84 s submerged in filtrated hematoxyline
3. 14 s under running water
4. 14 s submerged in an aqueous solution of 0.35% (w/v) sodium hydrogen carbonate, 2.0% (w/v) magnesium sulphate and a few crystals of thymol.
5. 14 s submerged in water
6. 14 s submerged in 20% eosine
7. 14 s under running water
8. 28 s in absolute ethanol

During each step of the staining process the slide was gently agitated in the solution. Hematoxylin was purchased from MERCK (product # 1.15938) and eosin from Acros Organics (product # 152881000). After staining the slides were washed in xylene and then covered with Pertex^®^ Mounting Medium.
