# Supplementary material for: Cutaneous squamous cell carcinoma characterized by MALDI mass spectrometry imaging in combination with machine learning
Source: Sci Rep. 2024 May 15;14:11091. doi: 10.1038/s41598-024-62023-0 (PMC11096391; doi:10.1038/s41598-024-62023-0)

Evaluation of predictive power

White marking: prediction error suggested by pathologist

Black marking: tissue area

567t2

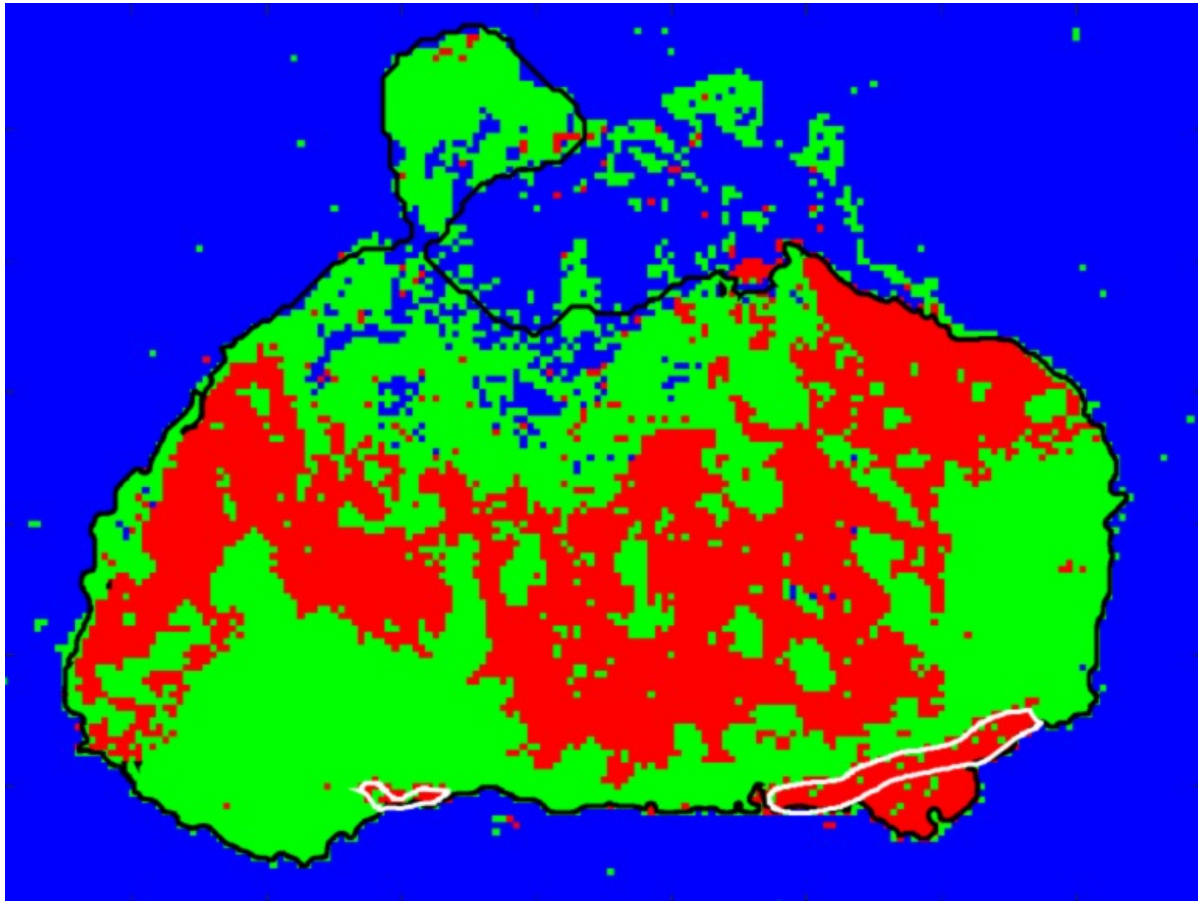

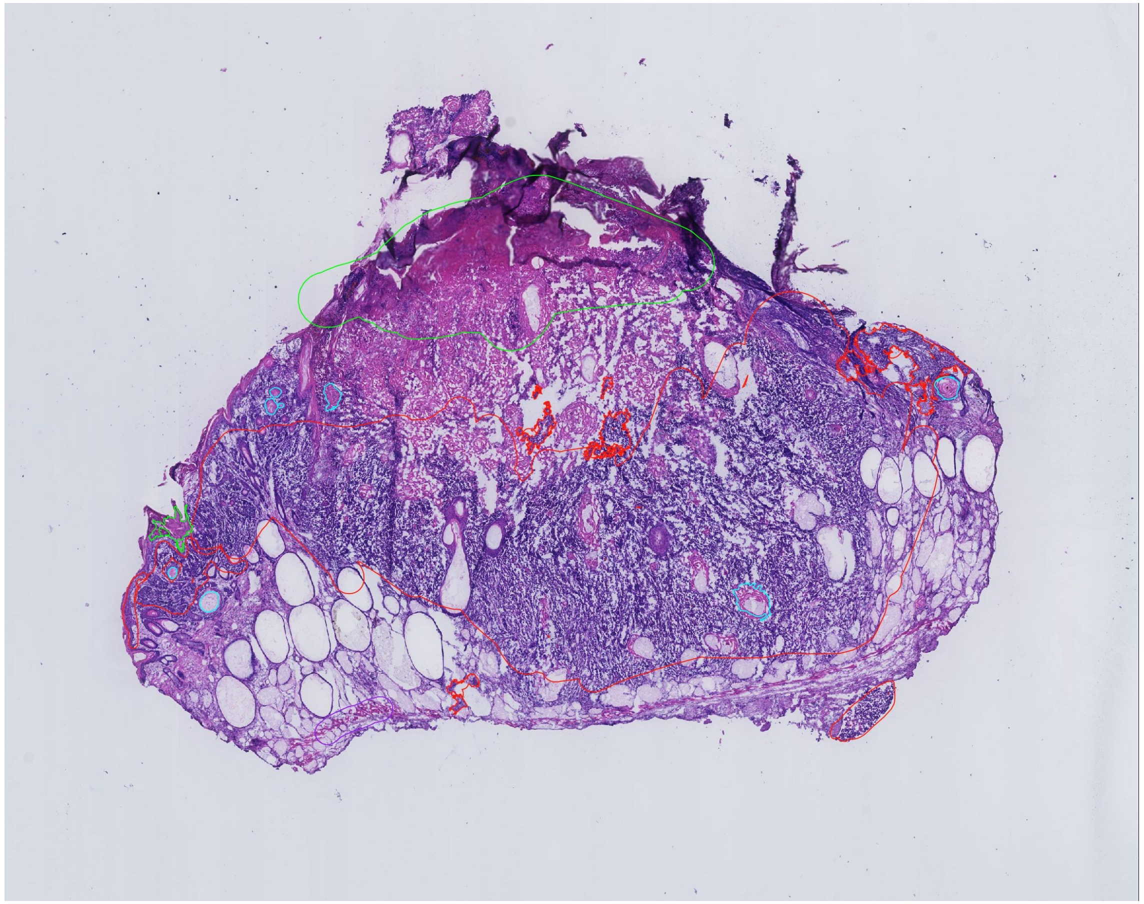

557

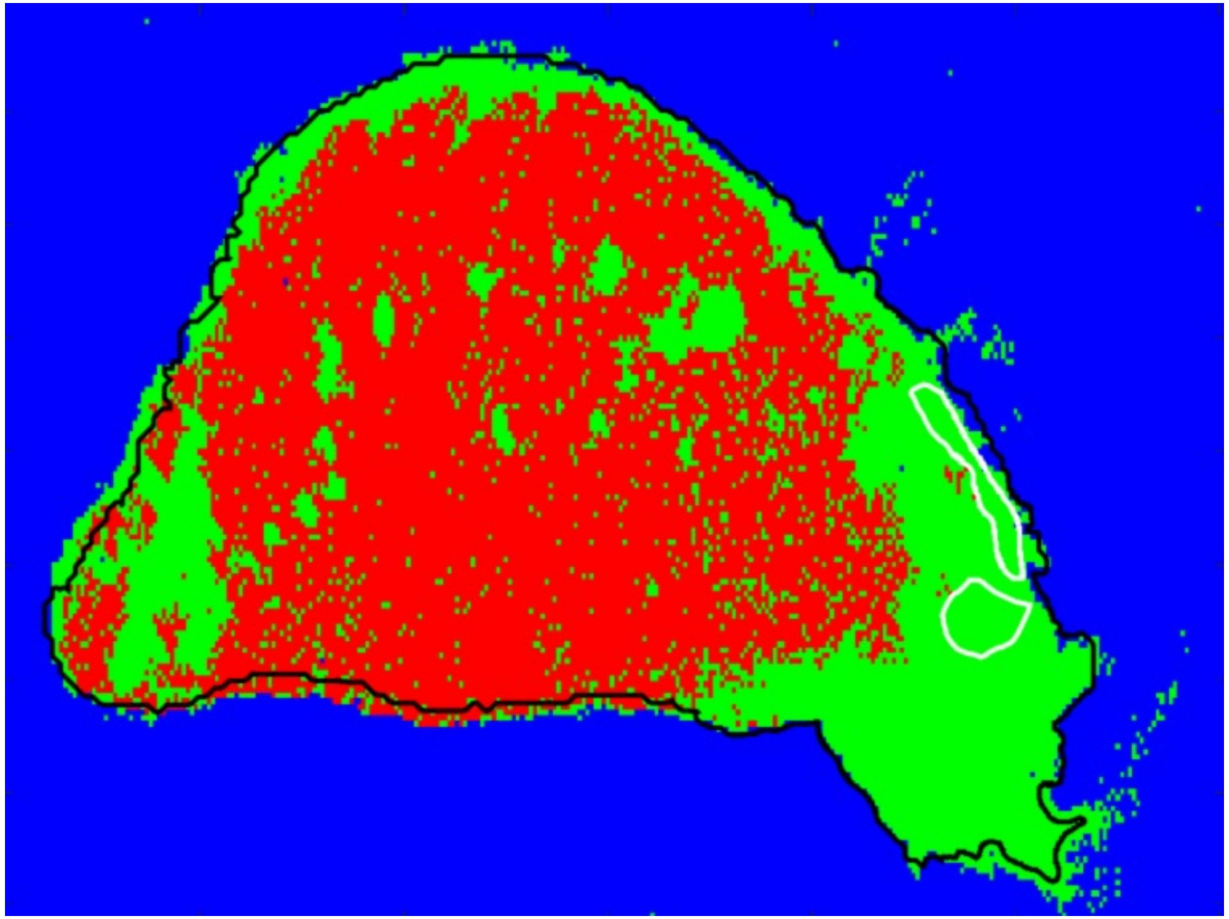

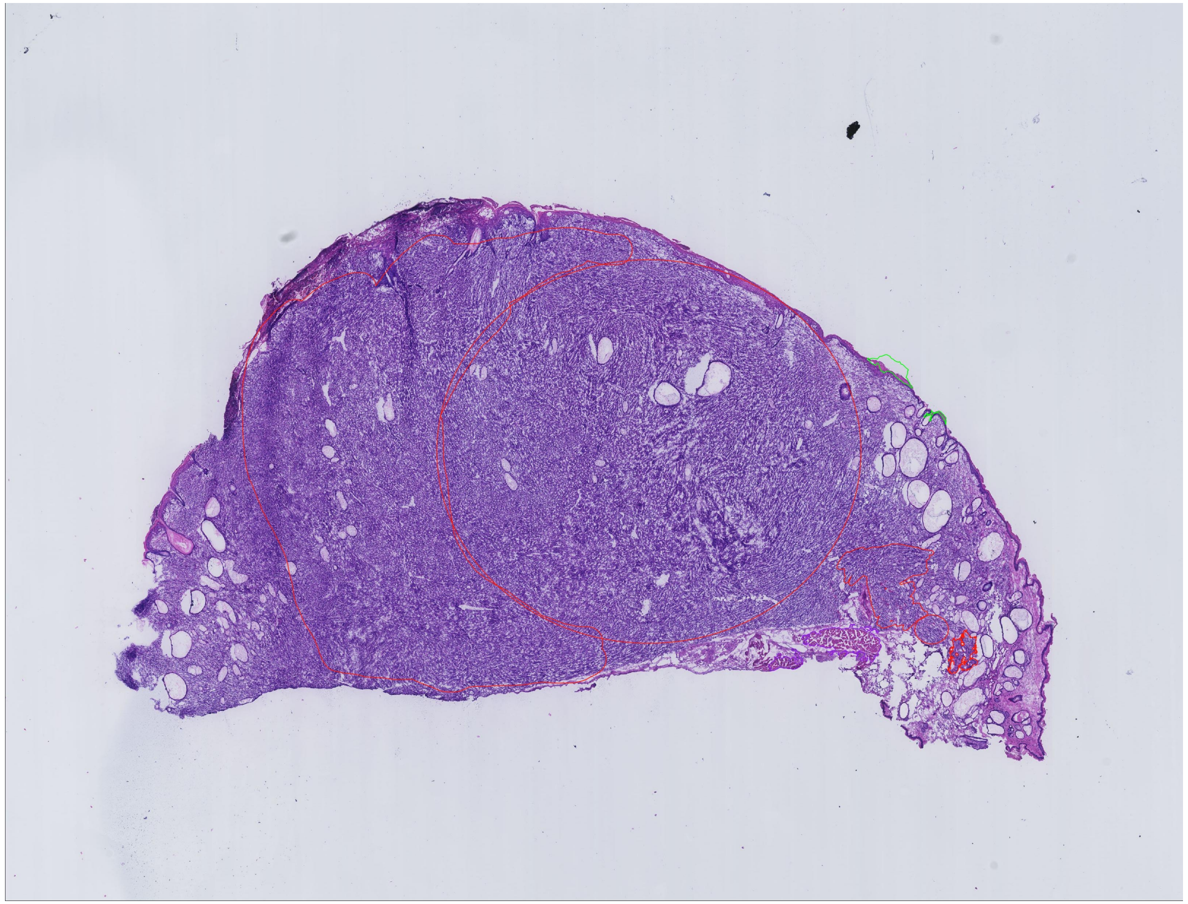

551

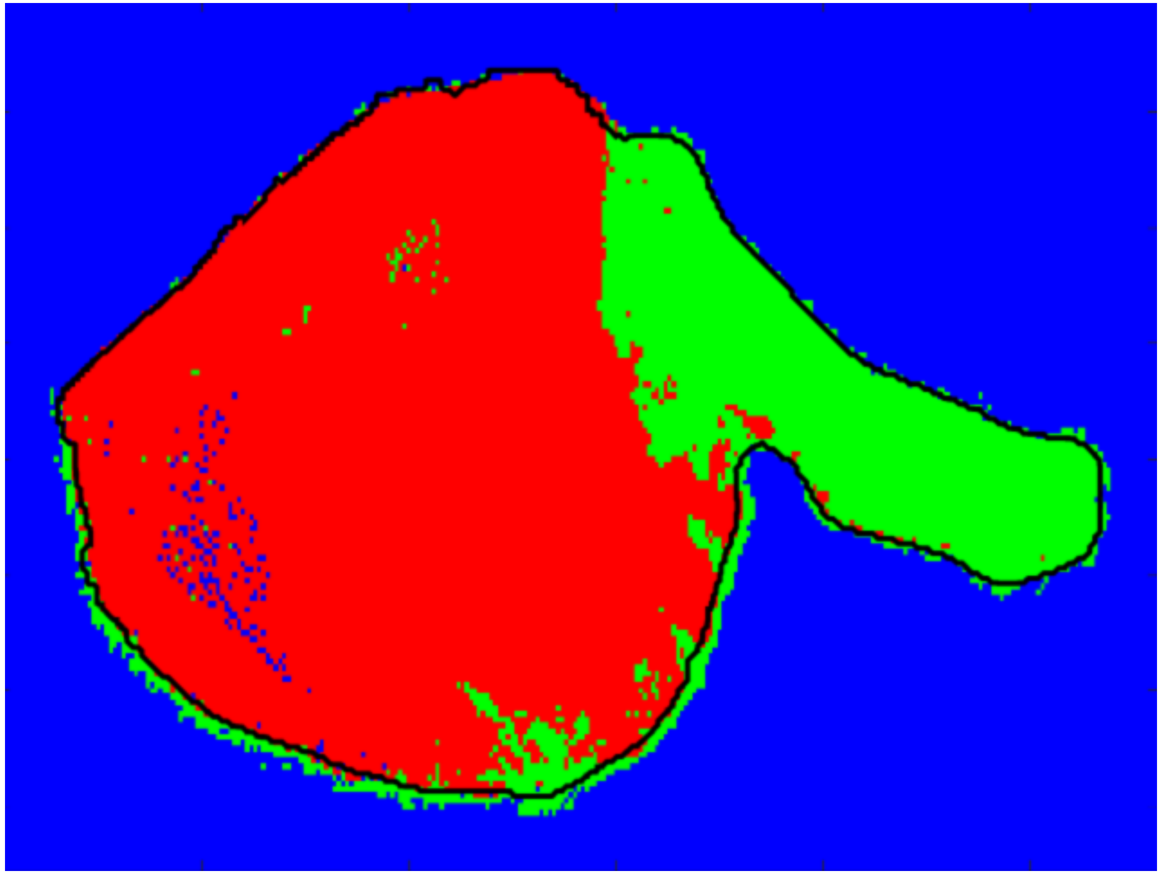

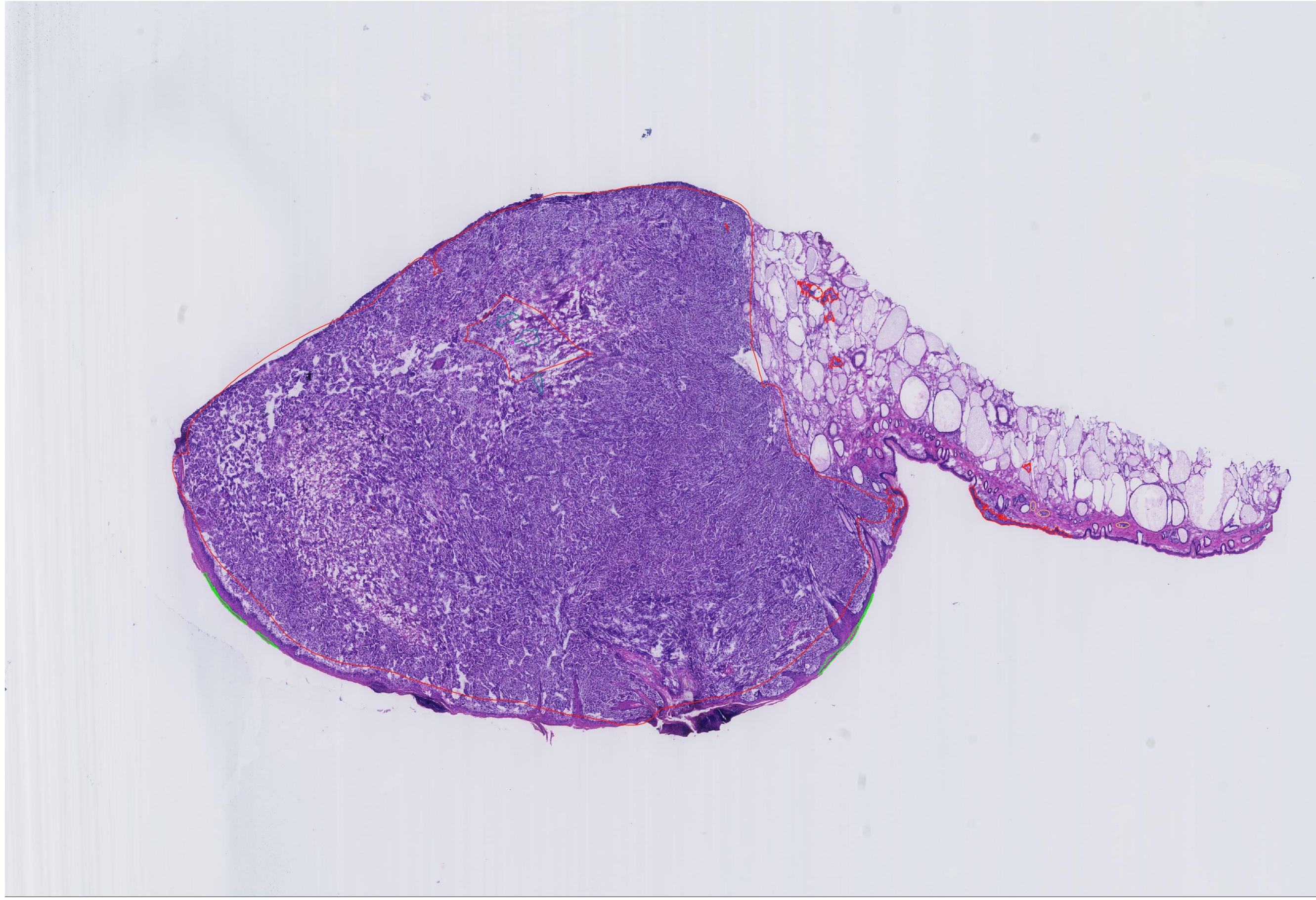

583

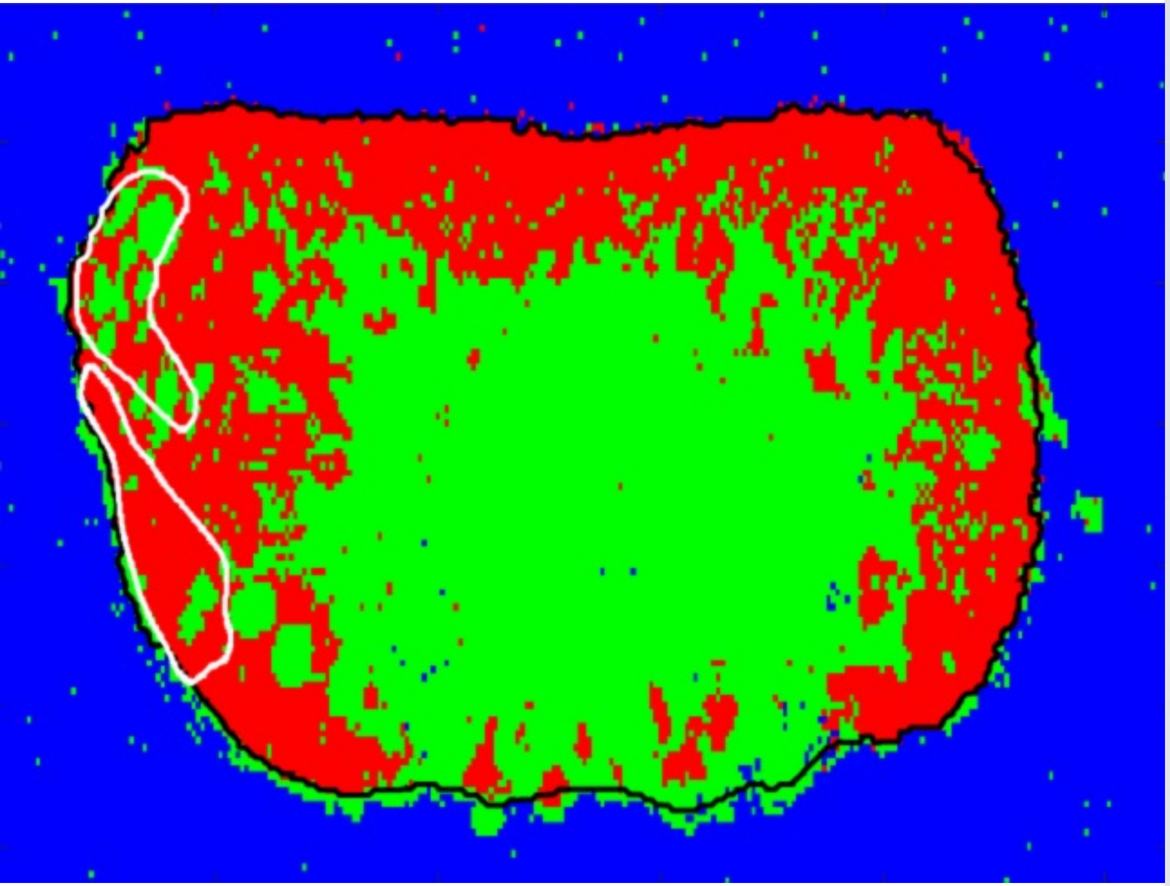

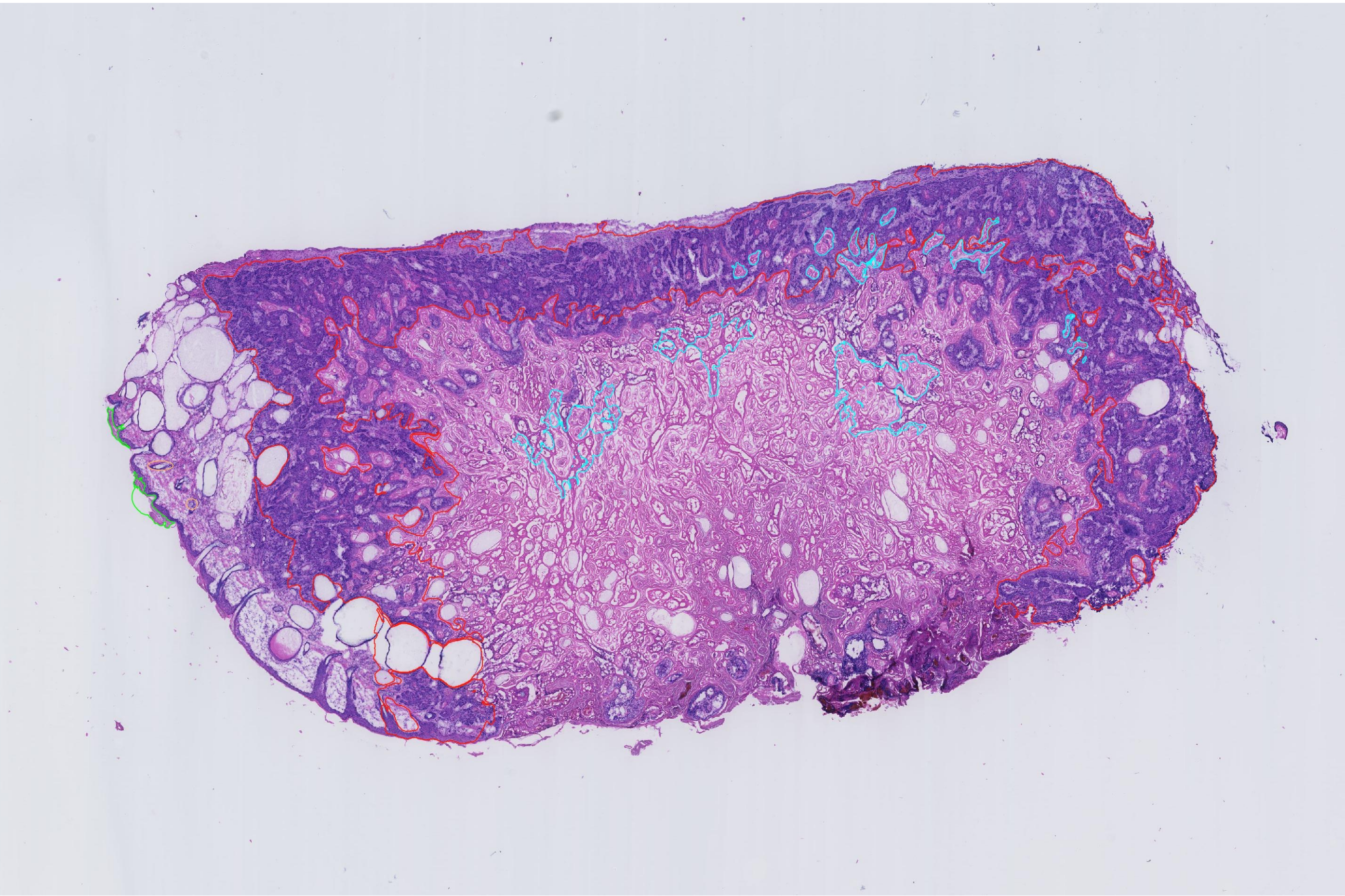

937

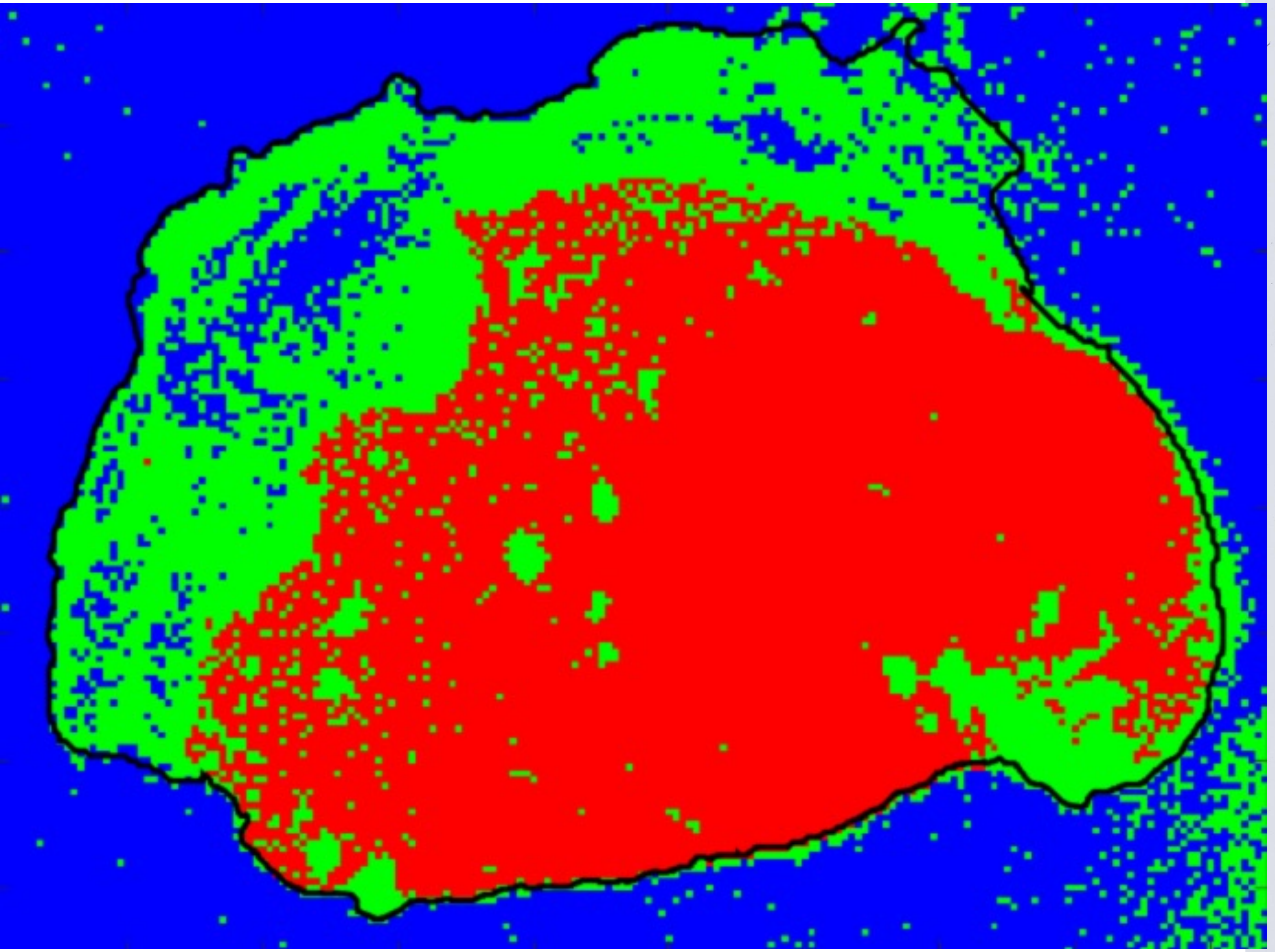

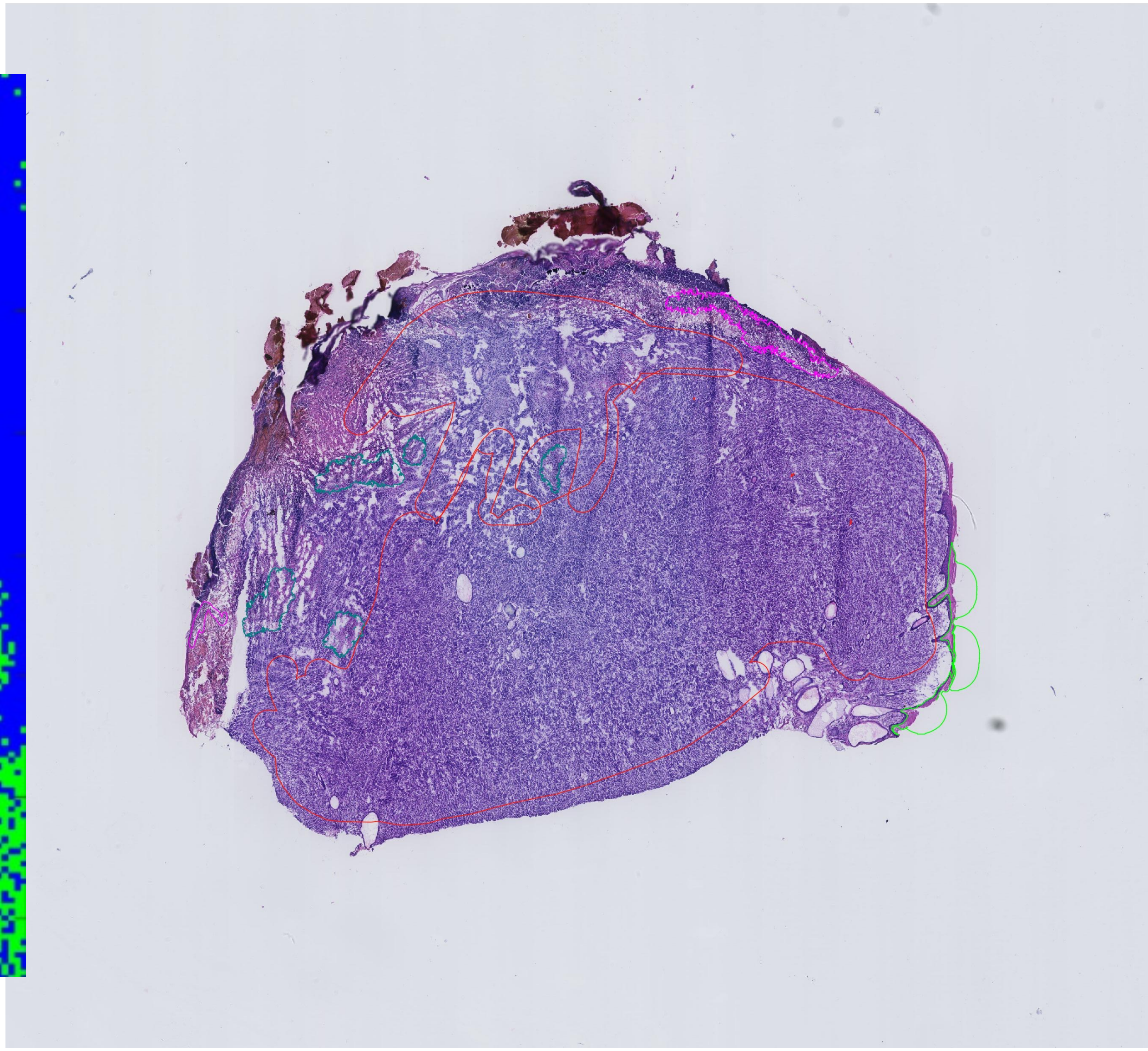

582

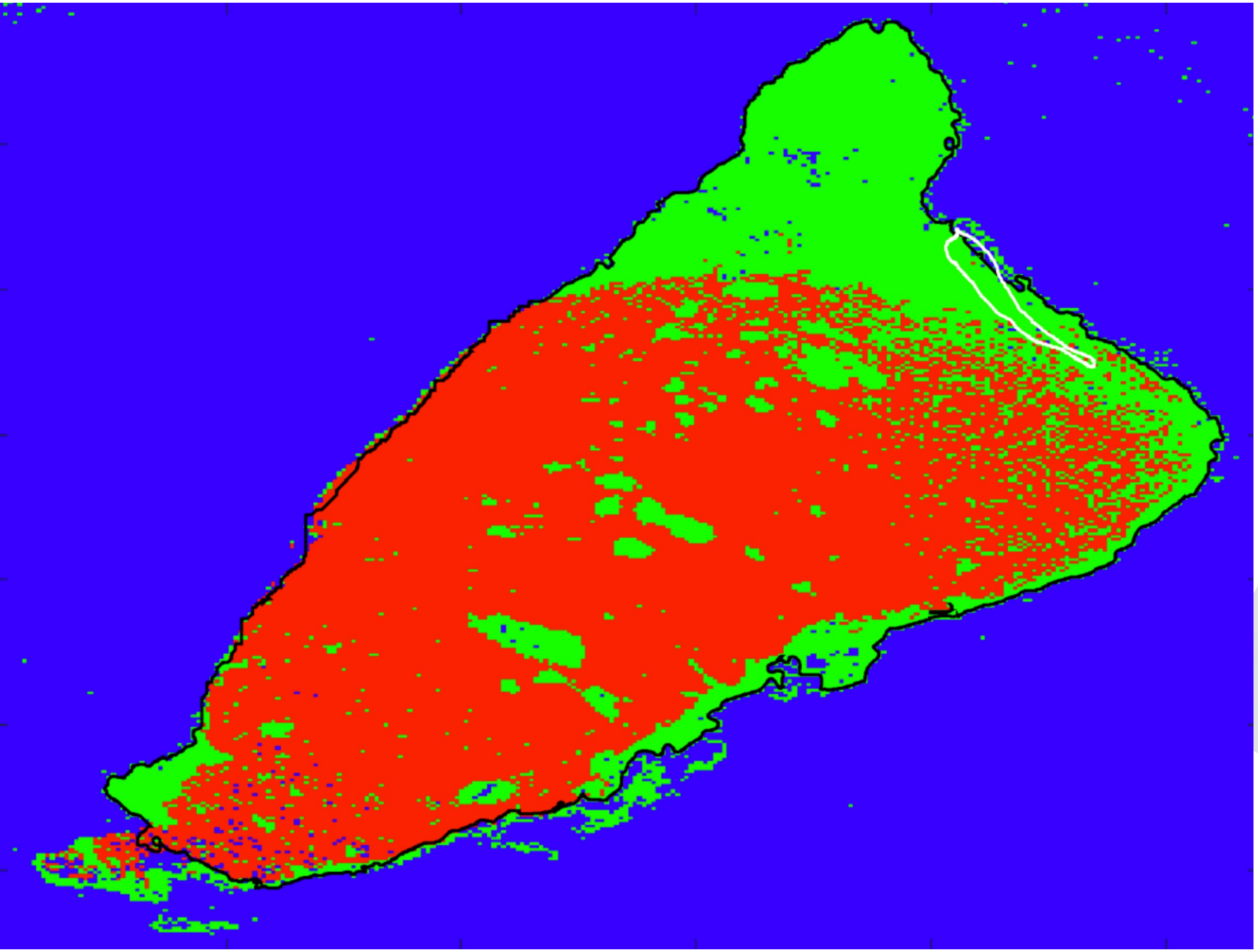

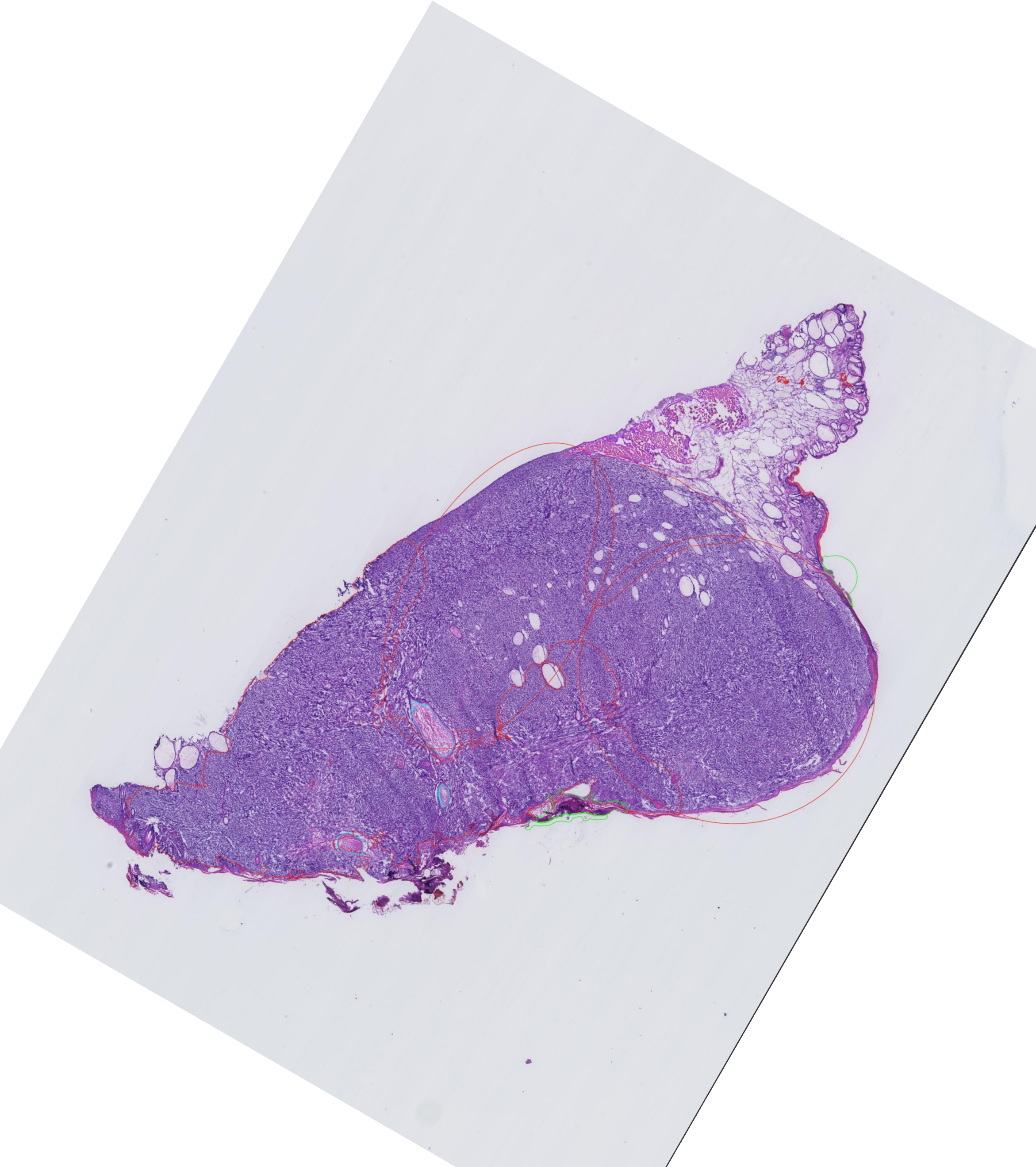

567t1

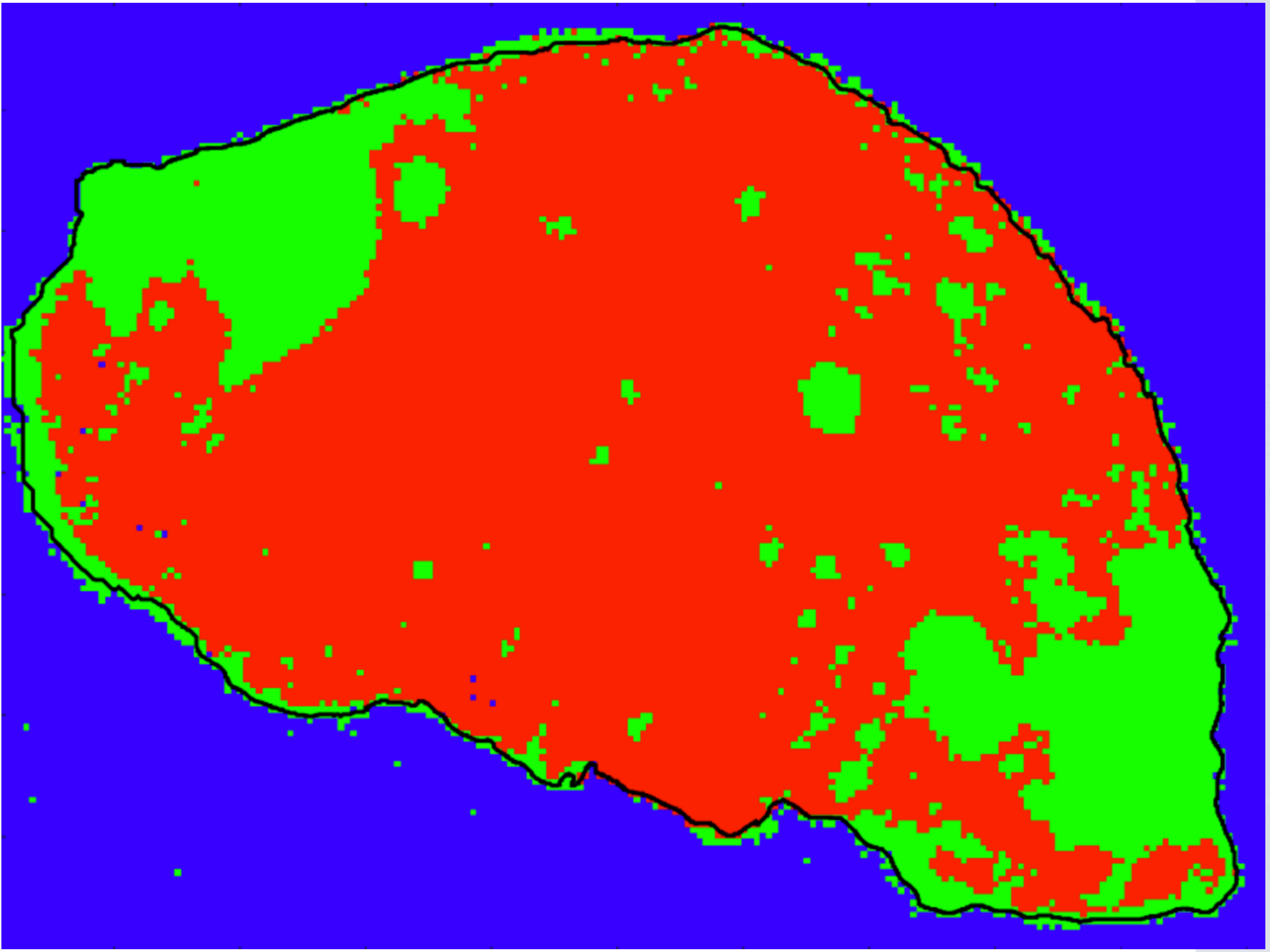

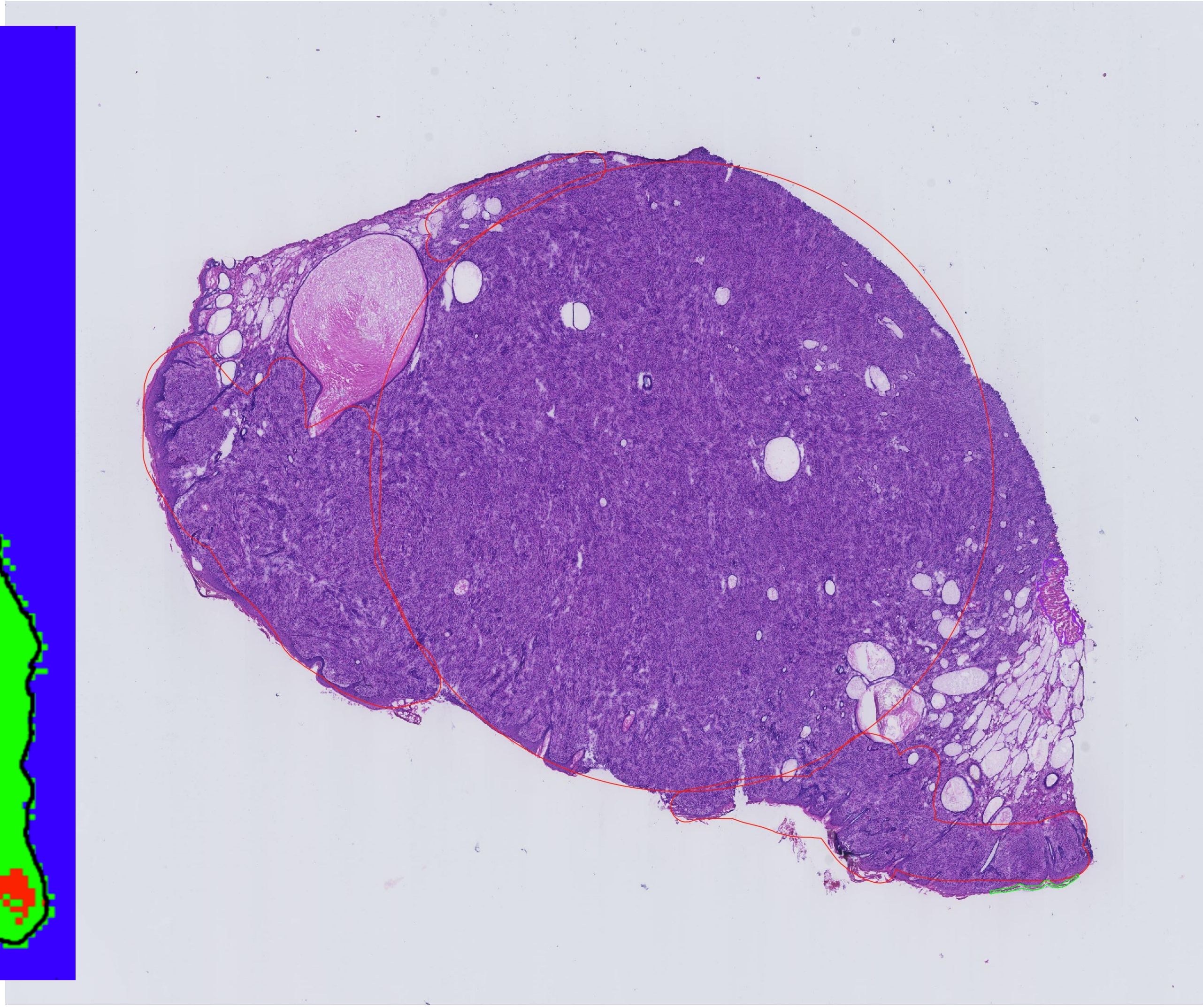

610

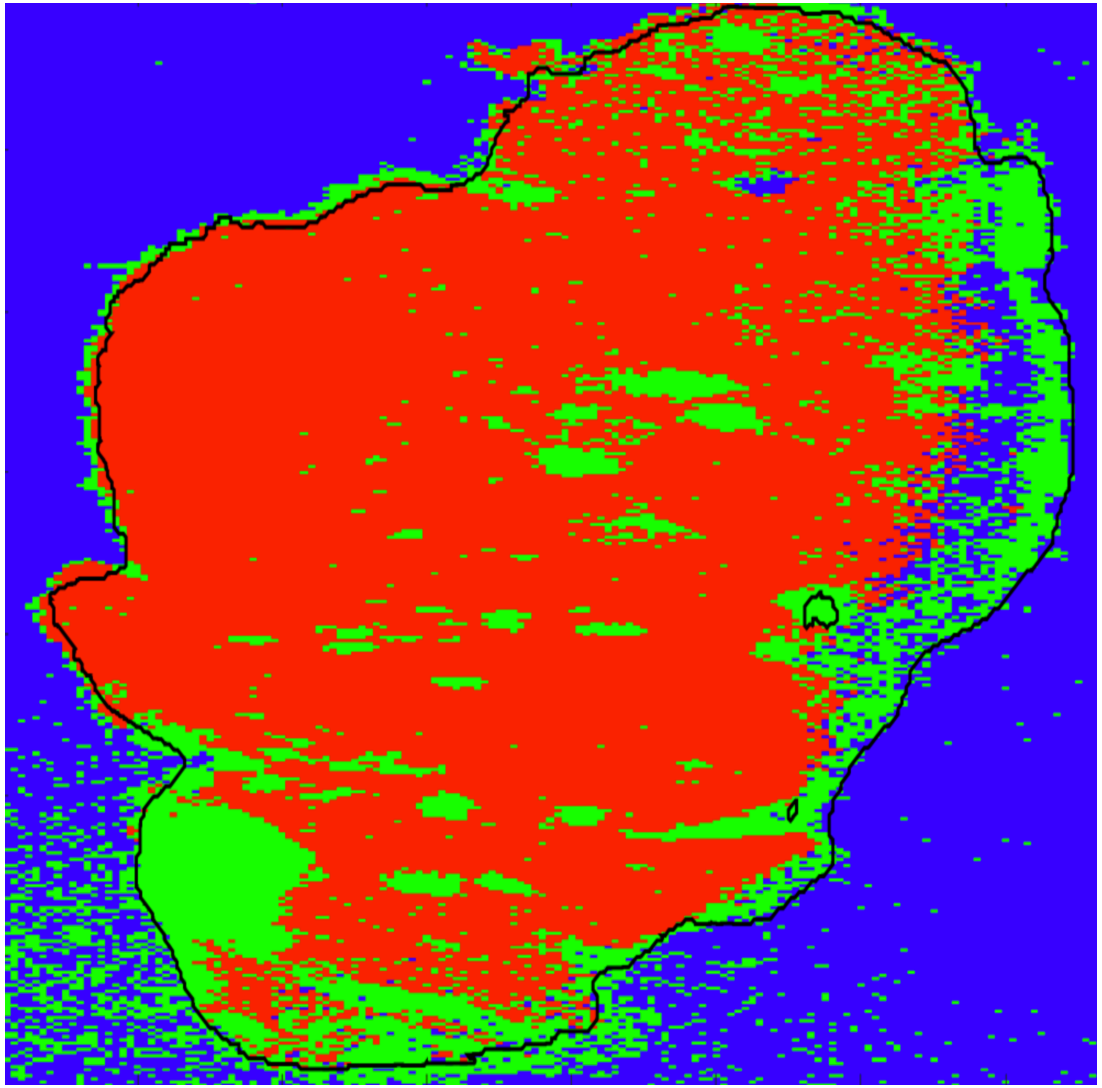

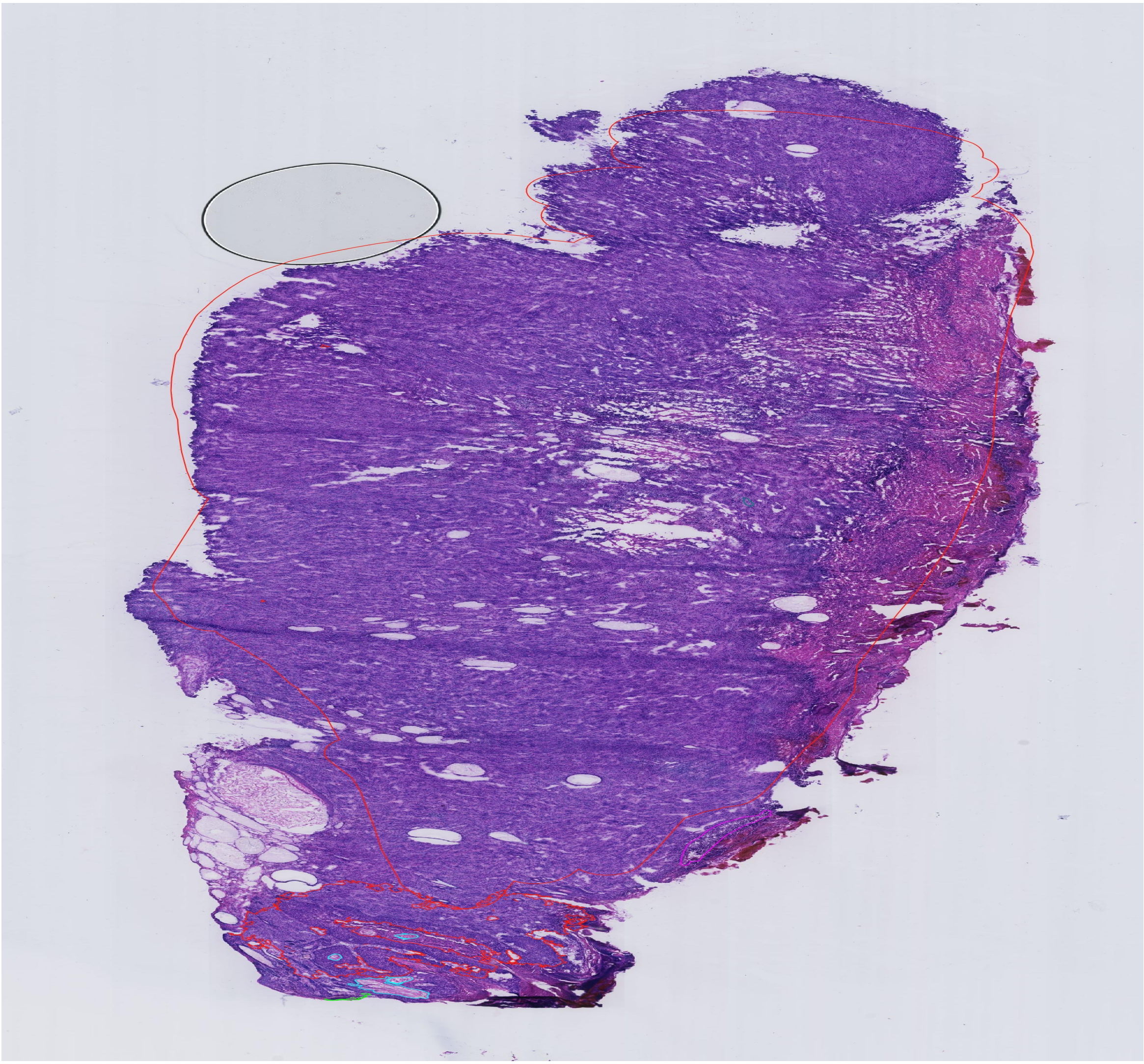

254t1

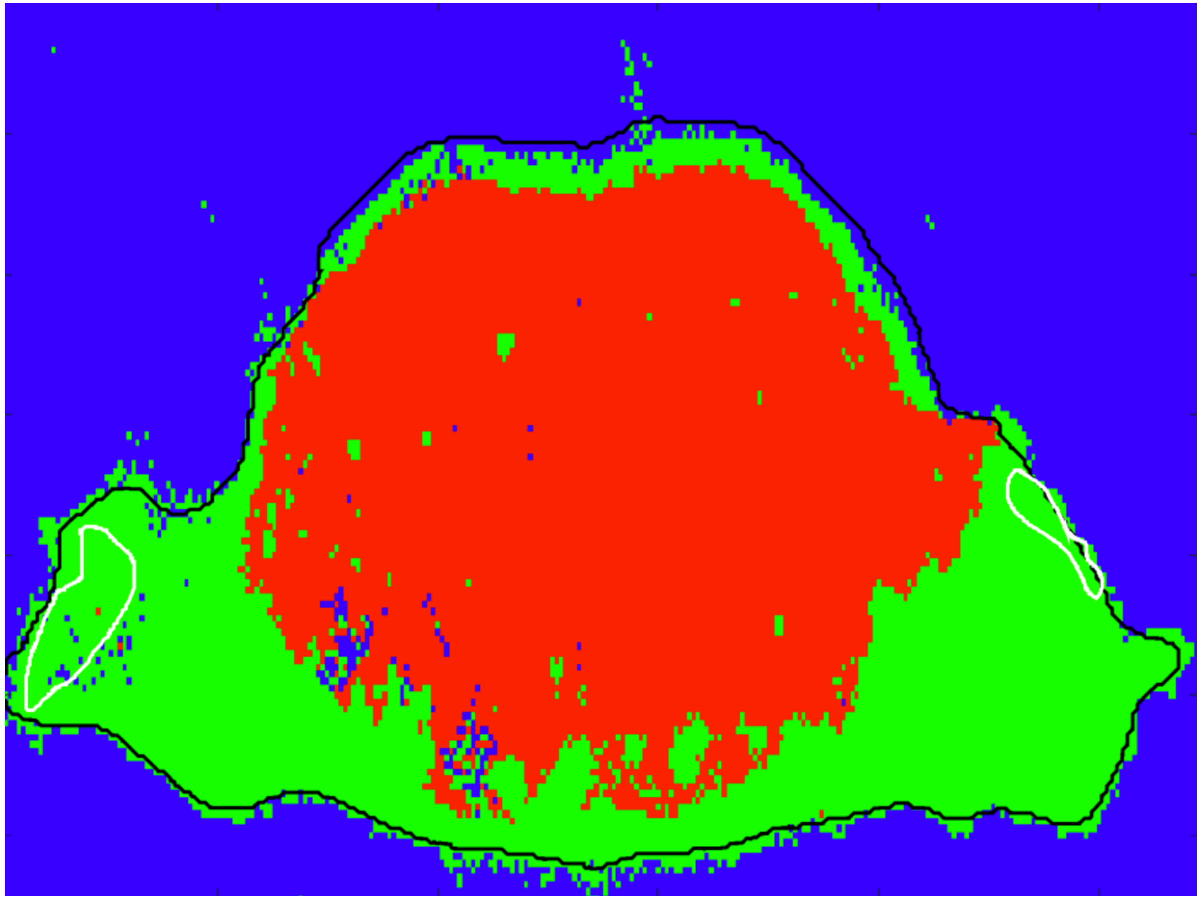

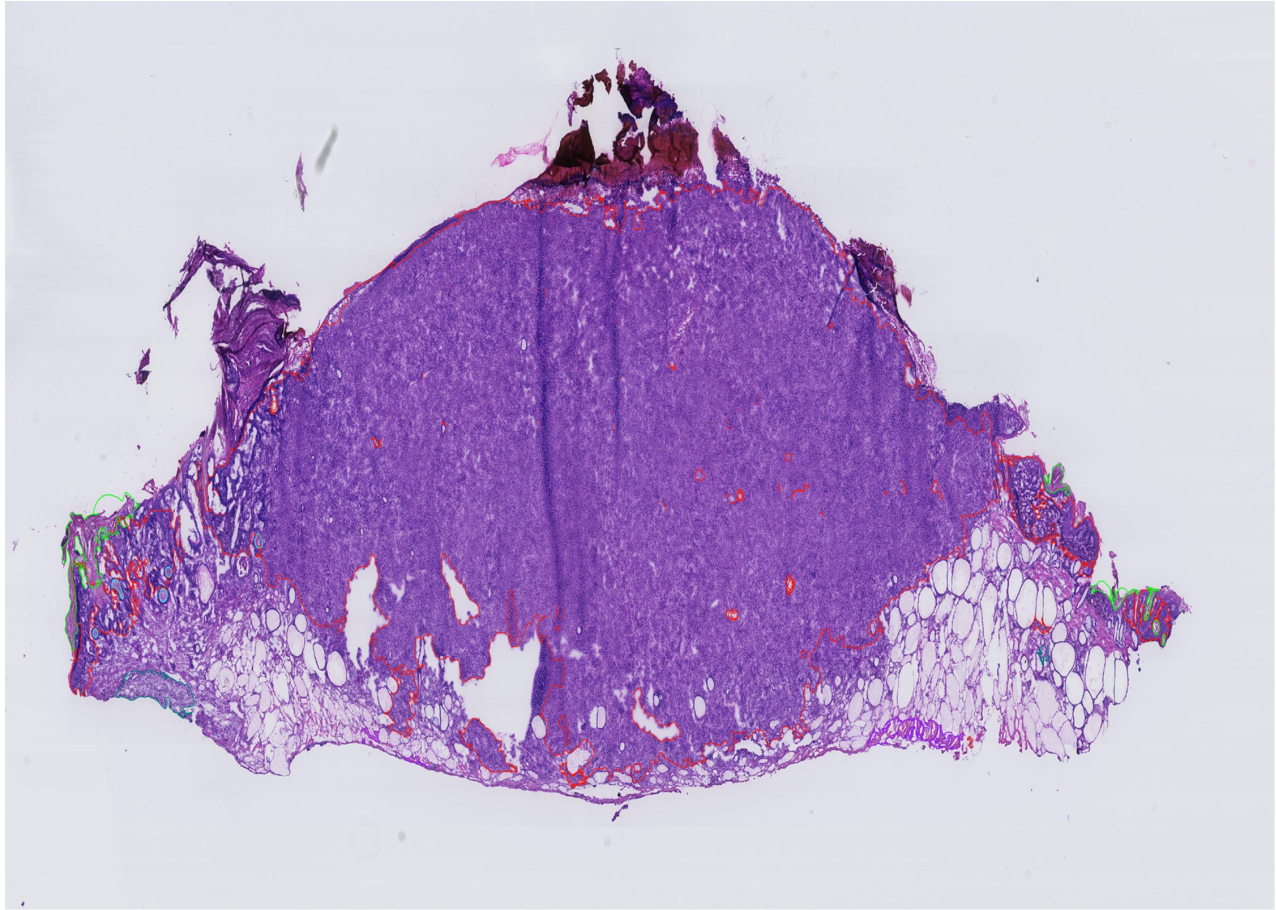

208

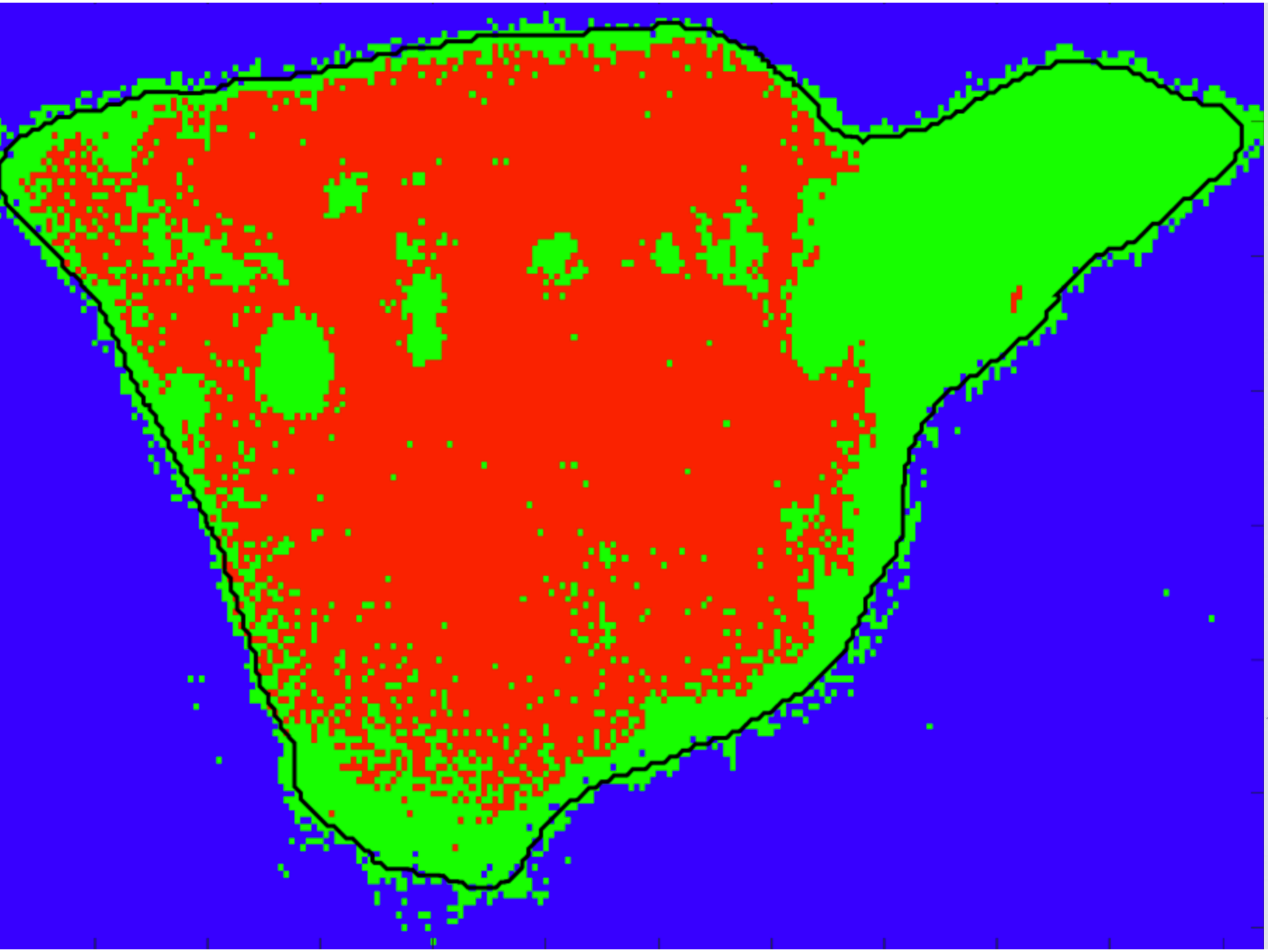

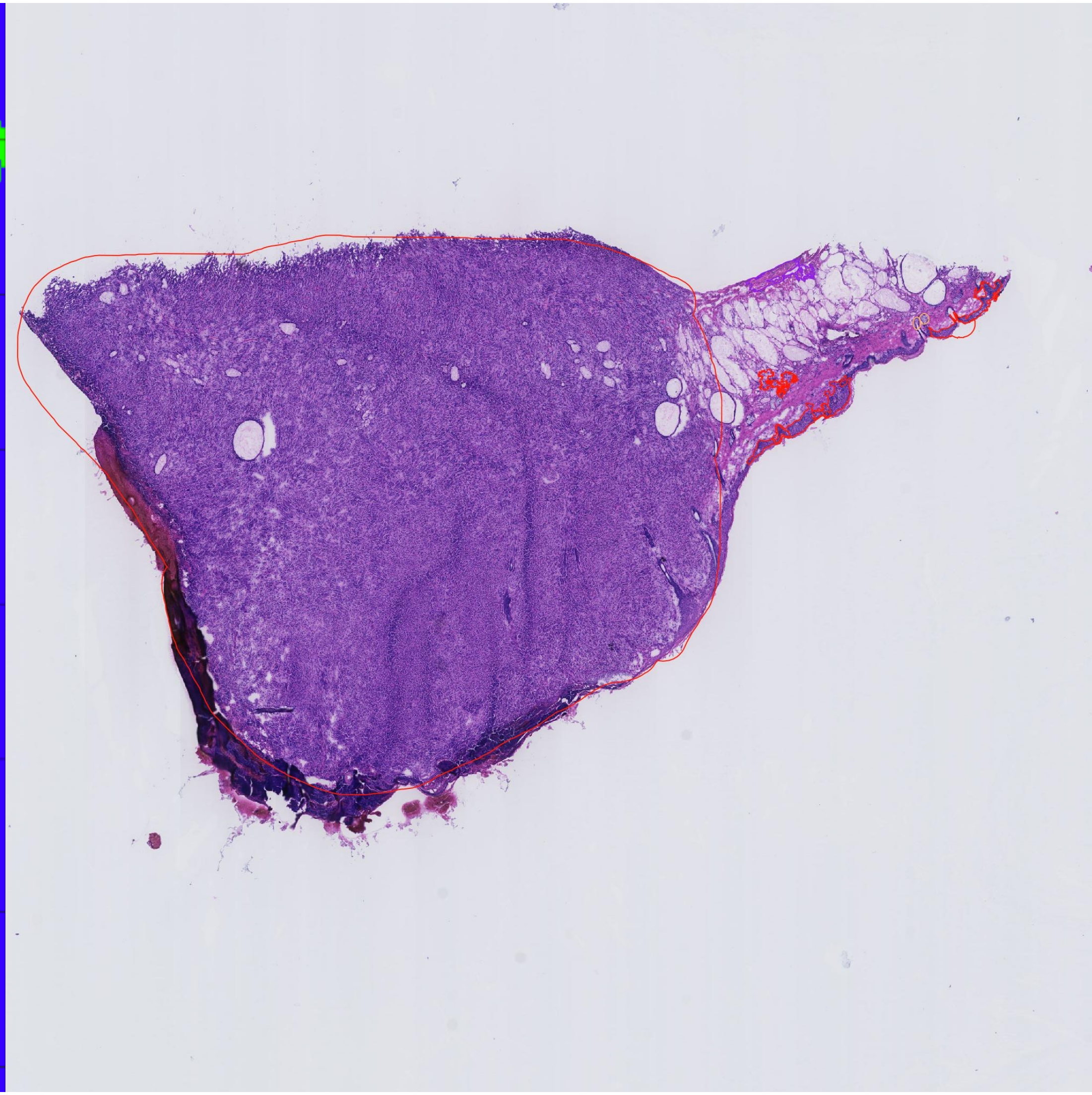

261t3

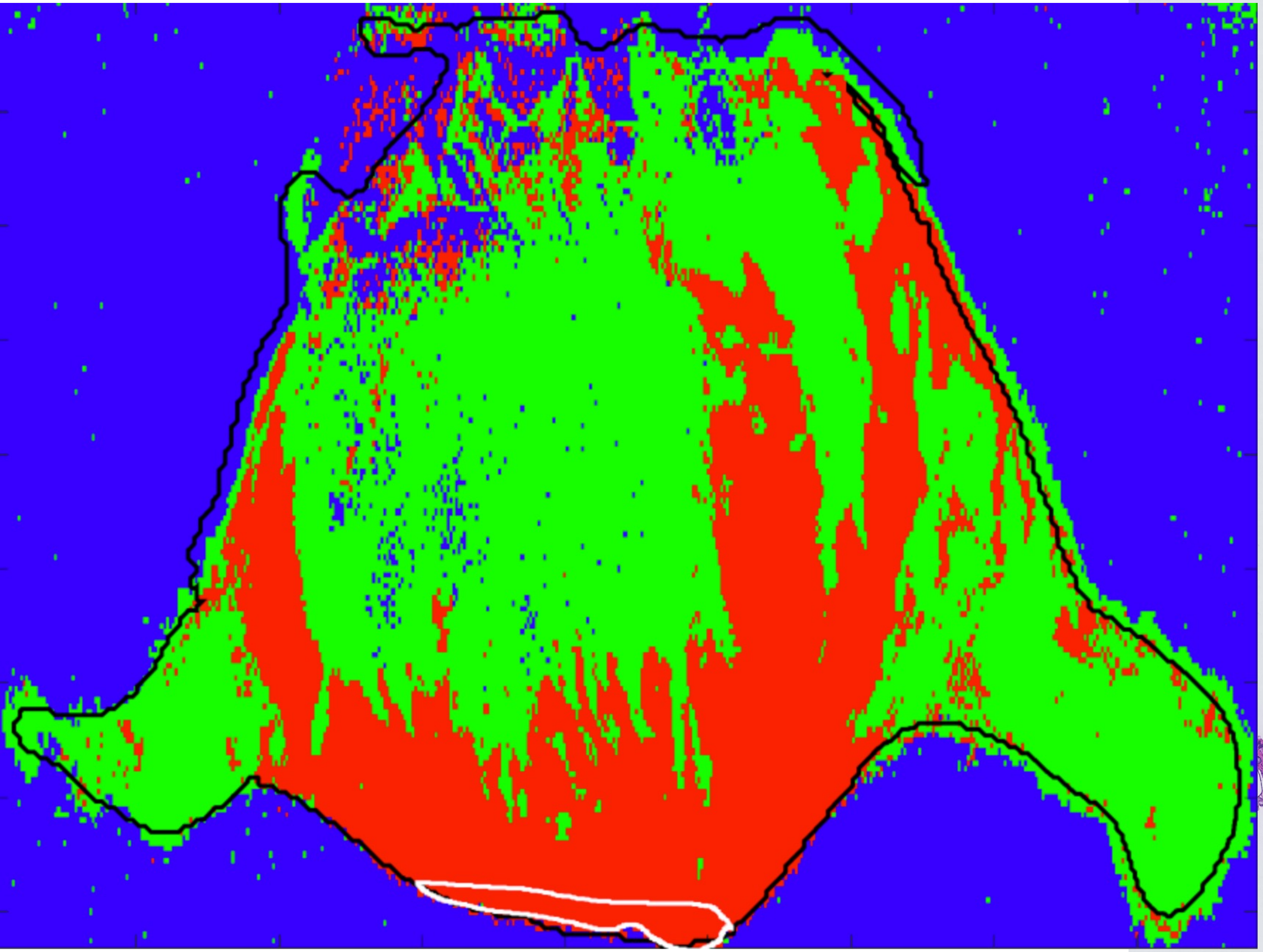

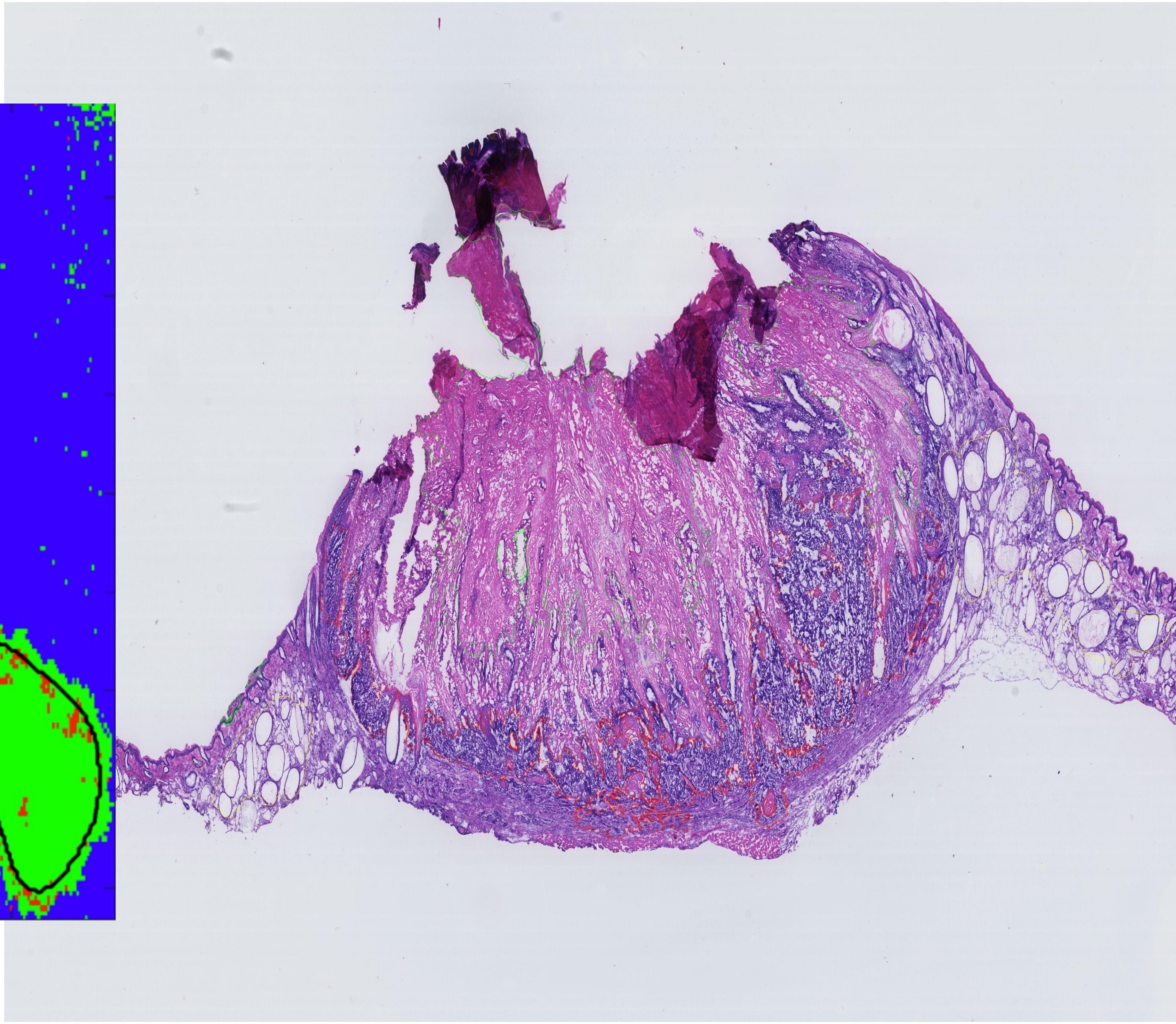

232

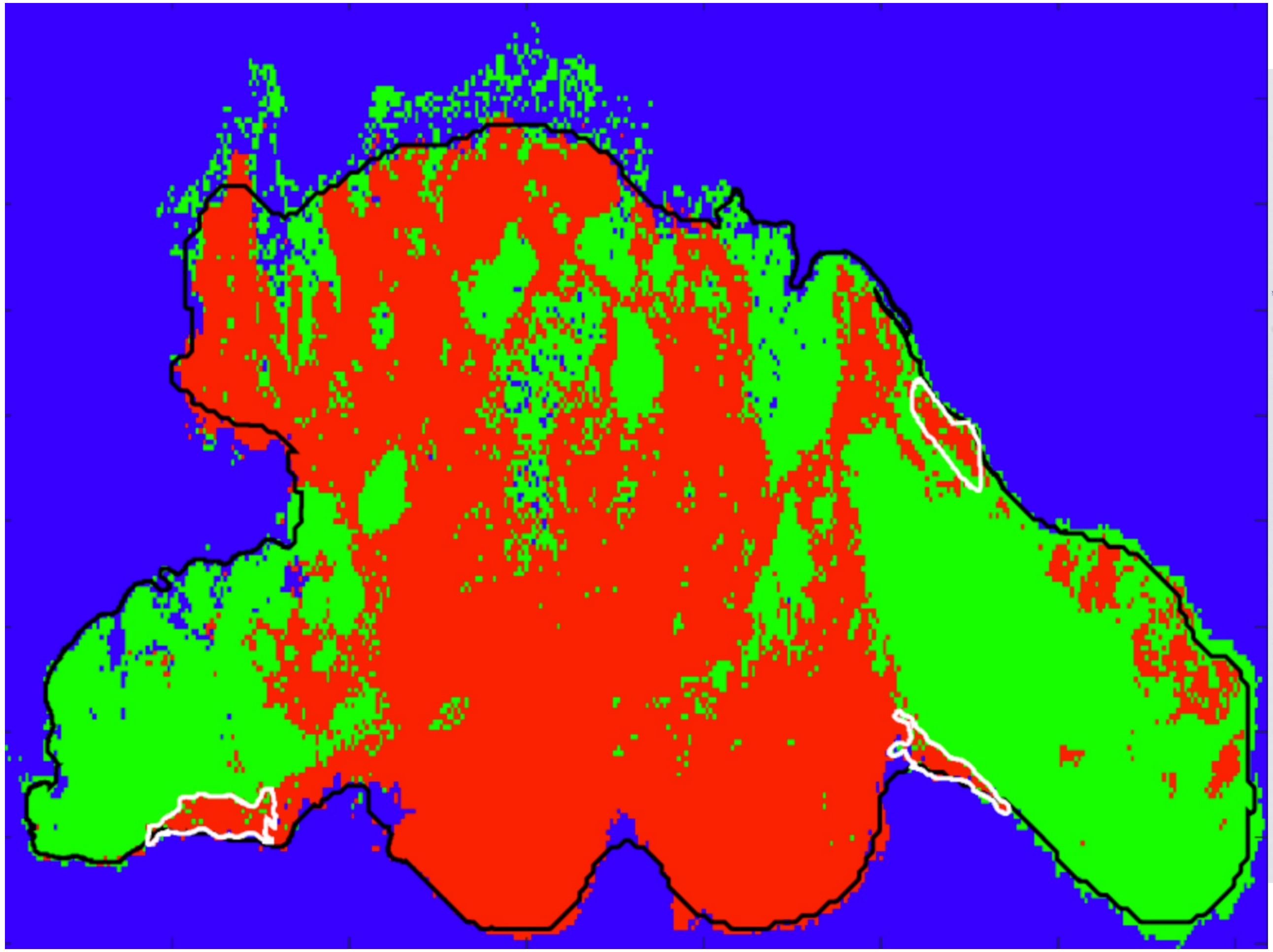

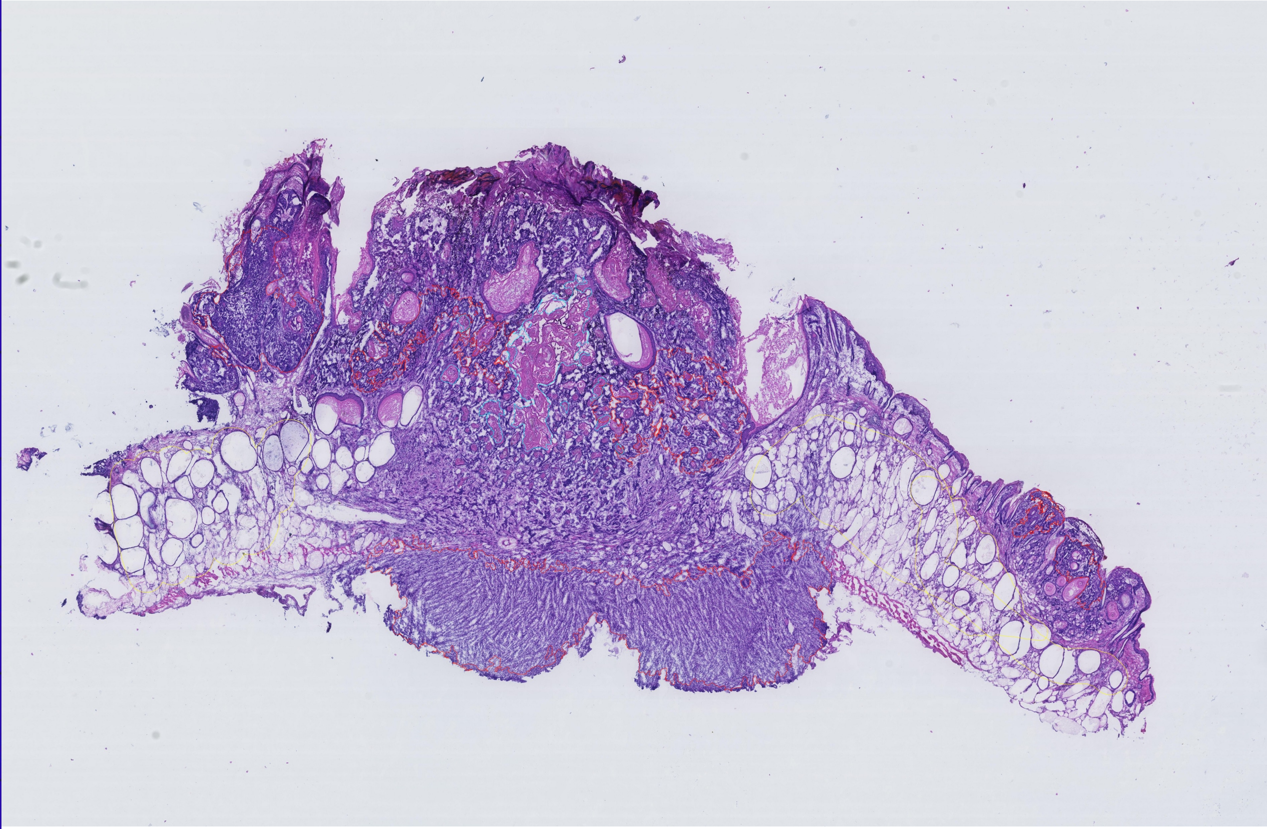

254t2

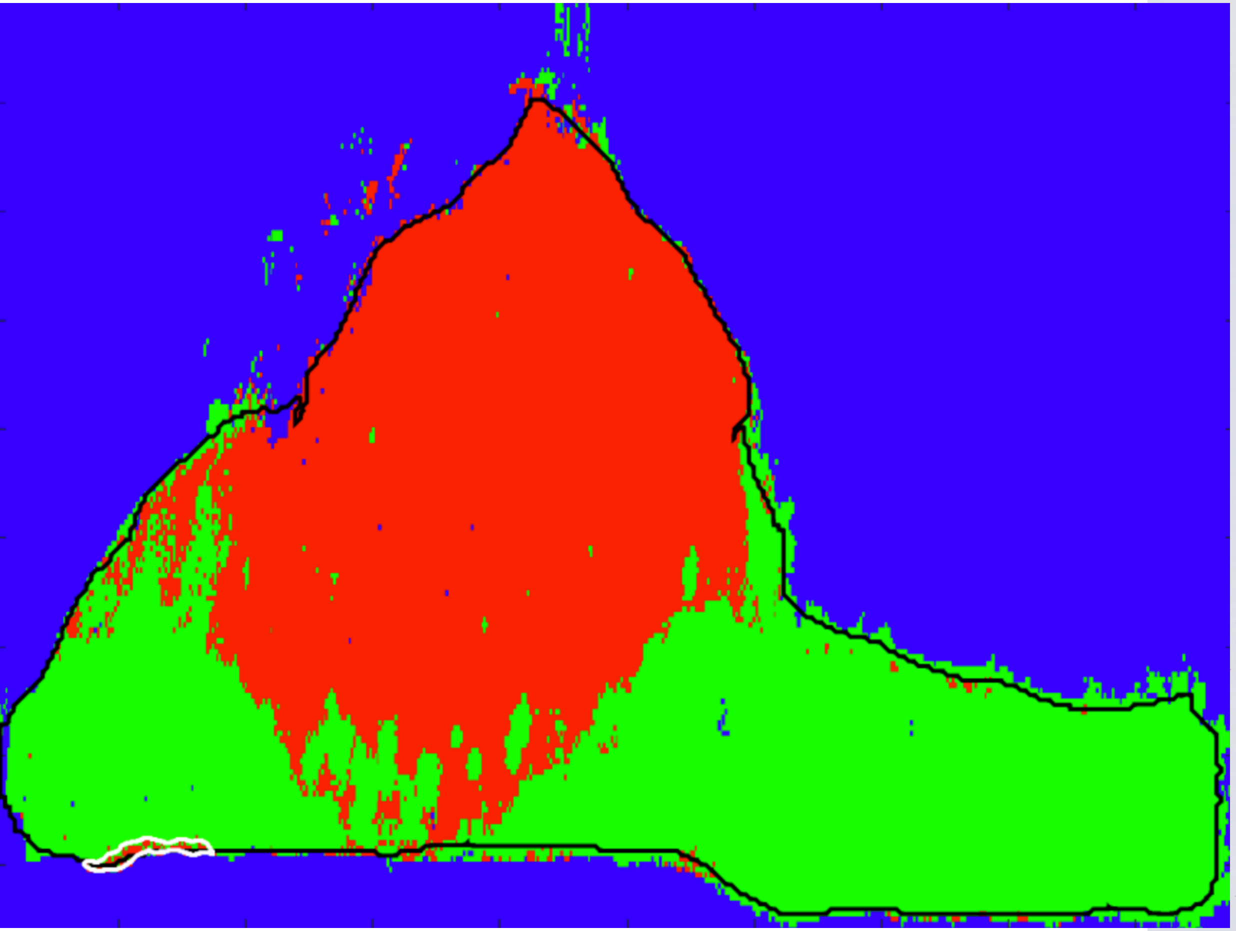

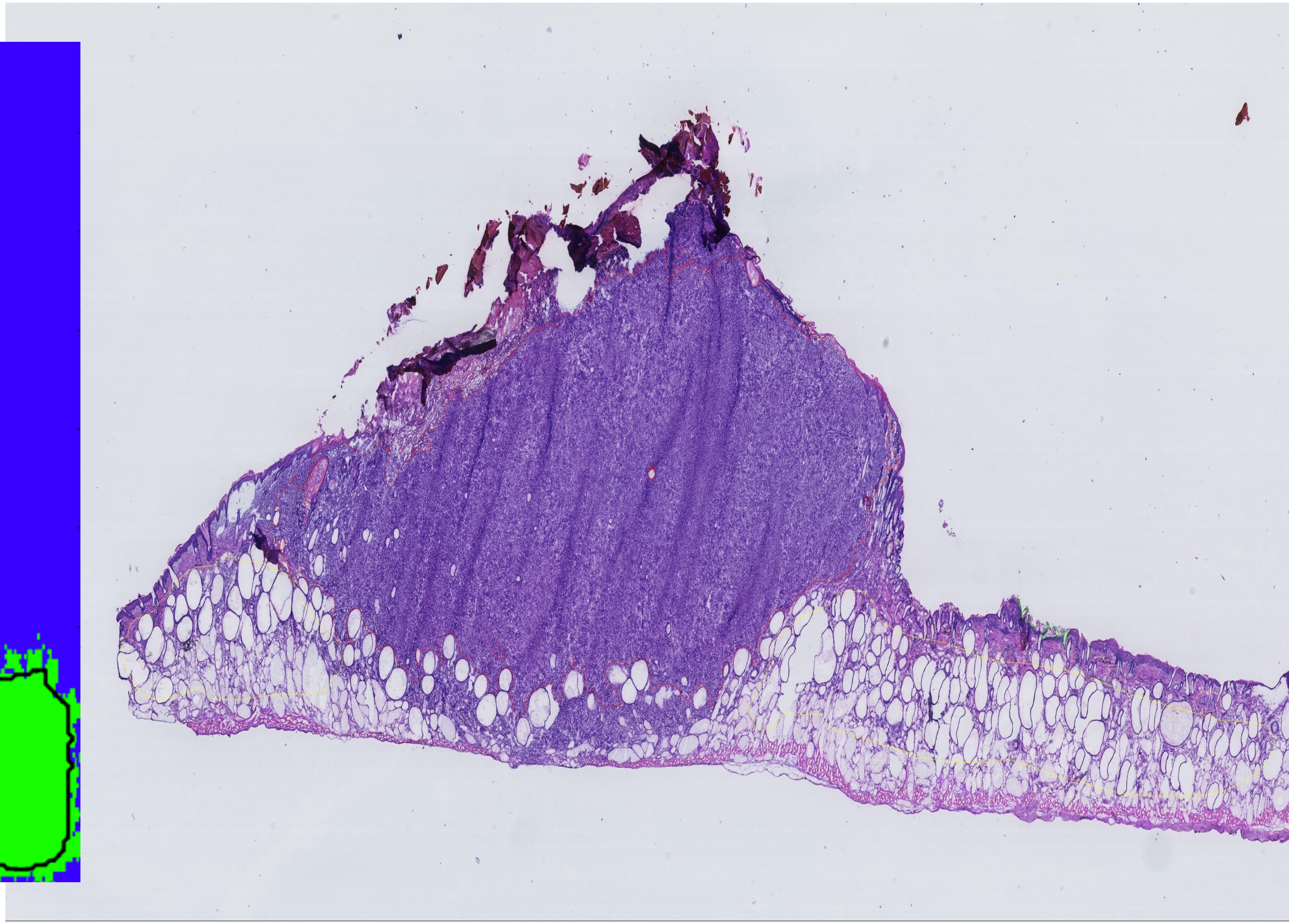

260

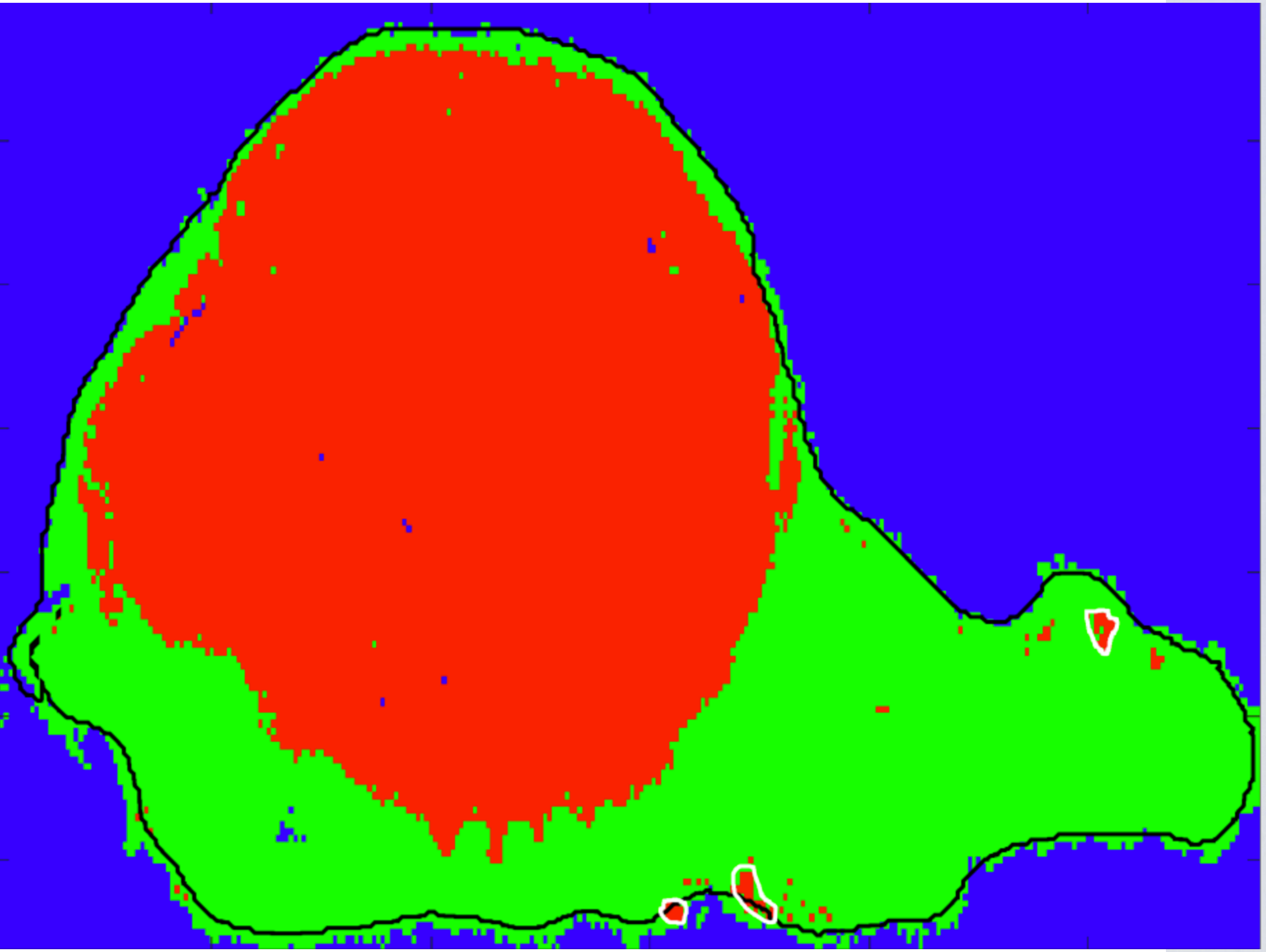

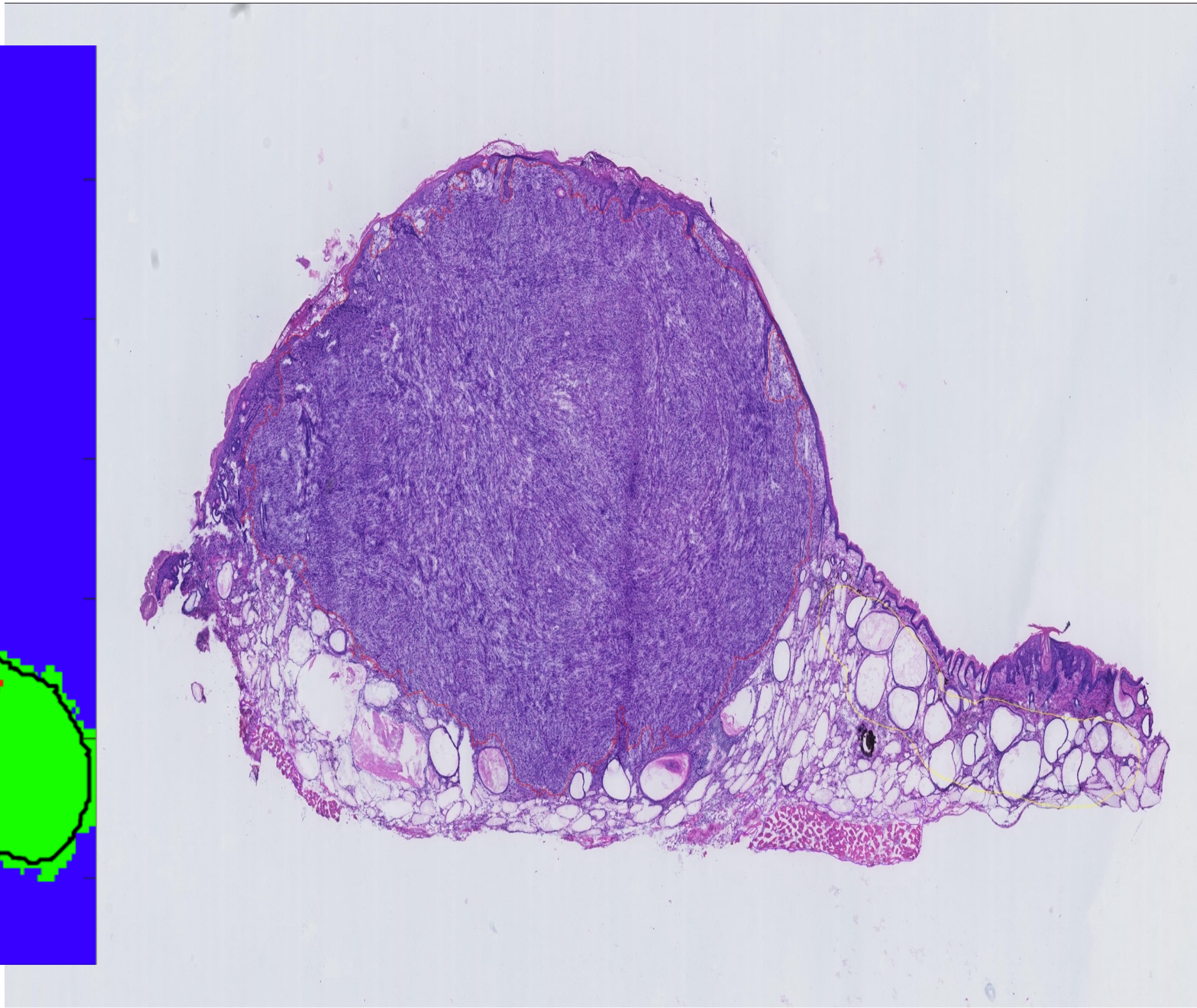

267

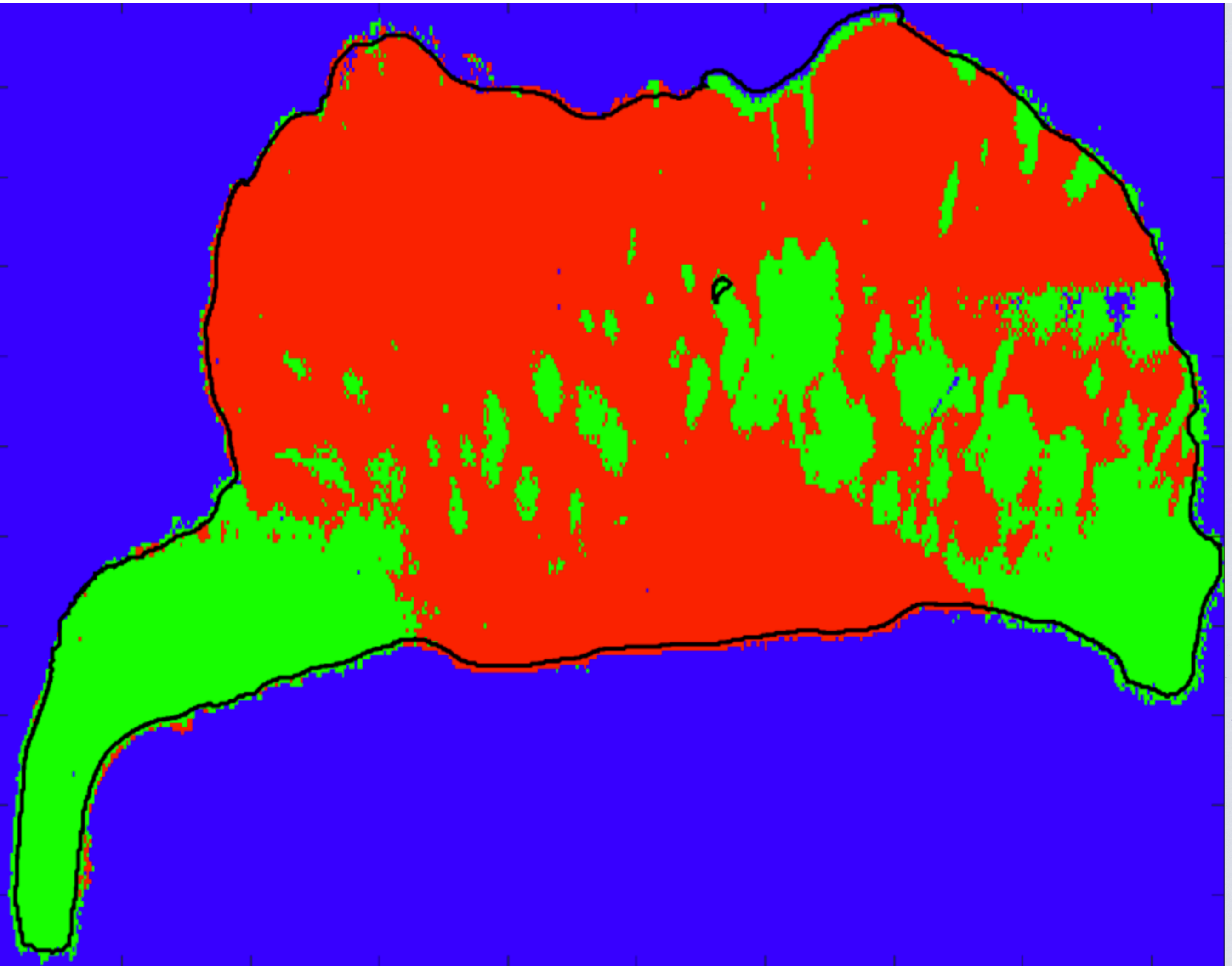

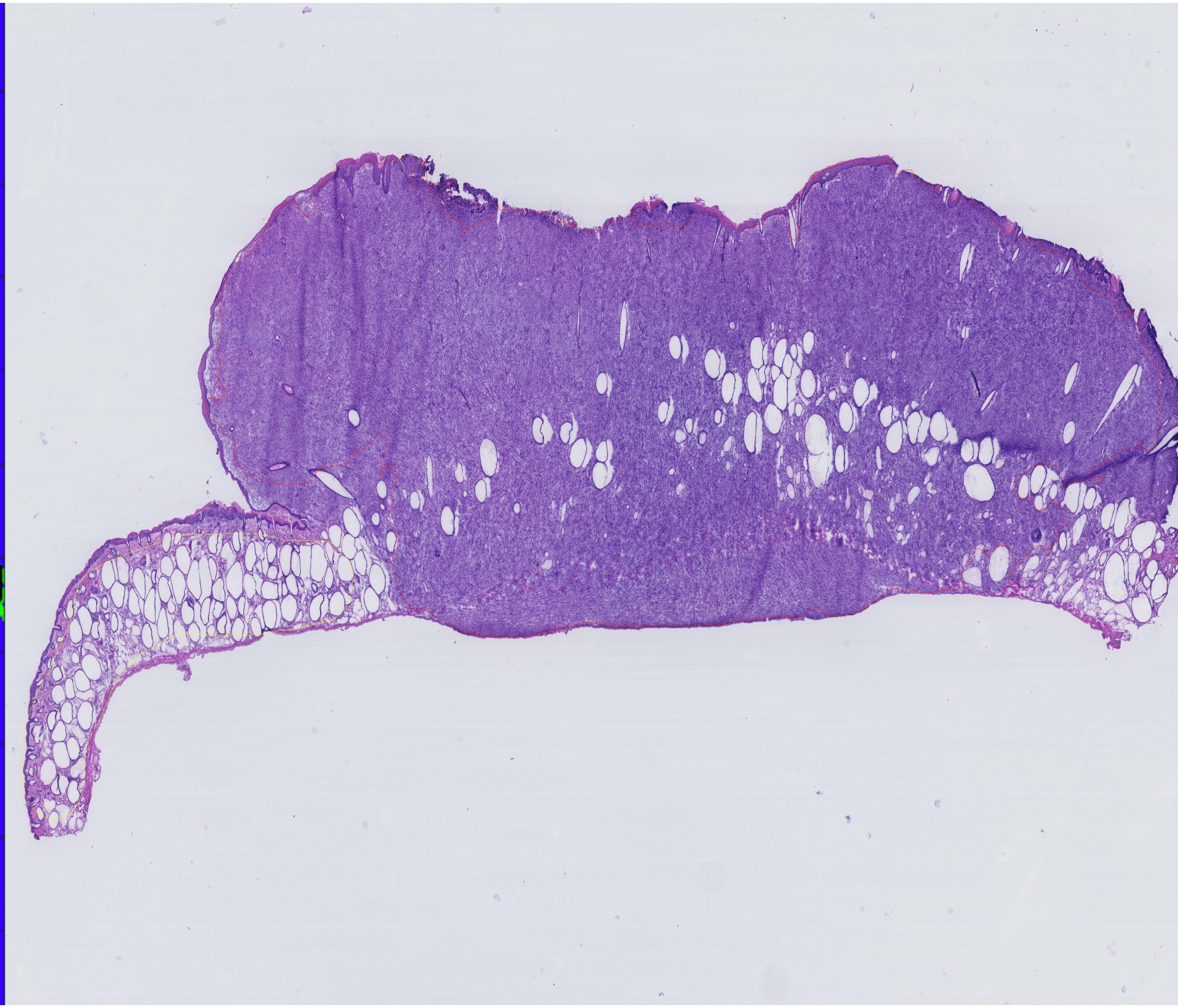

261t1

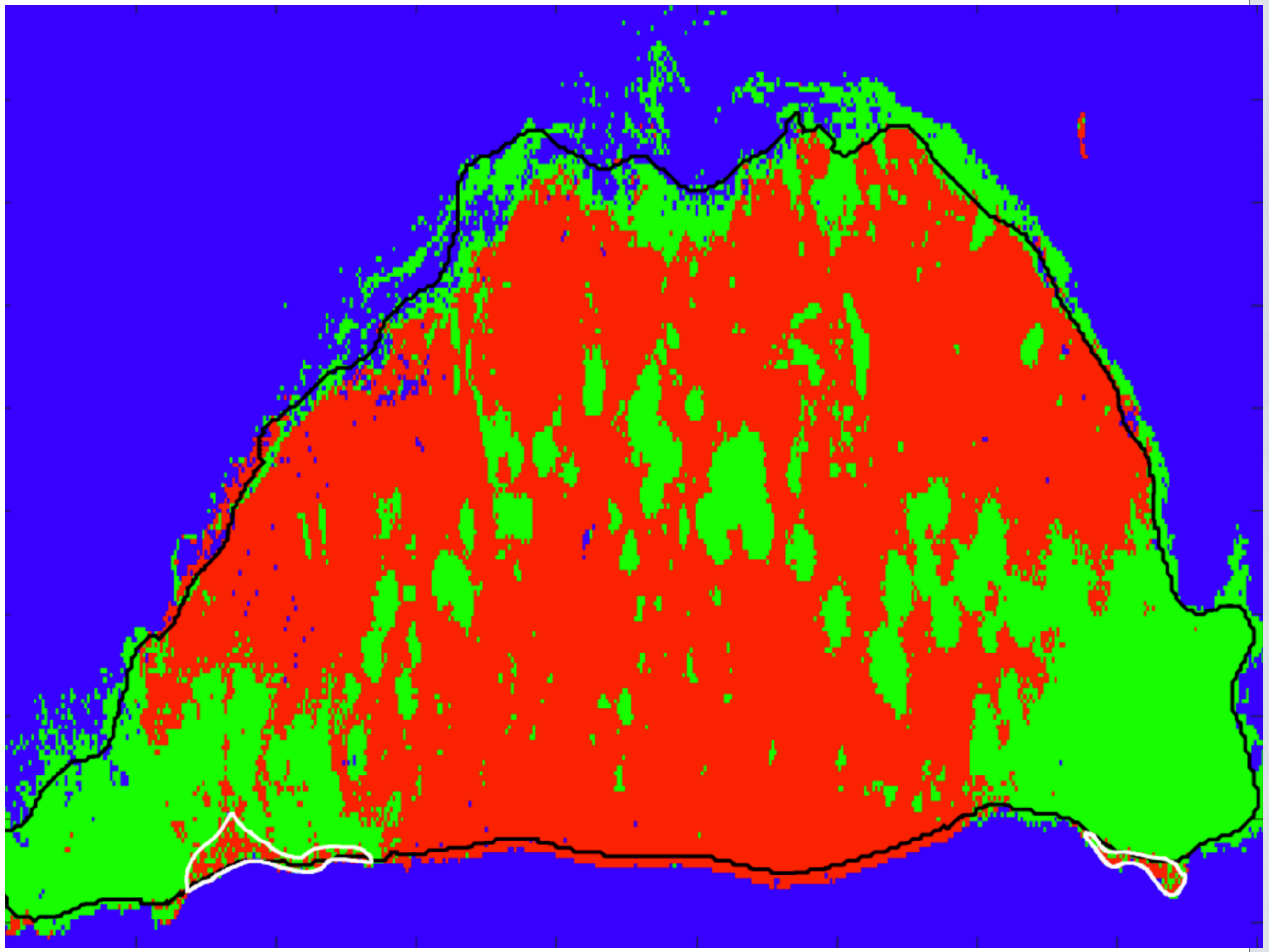

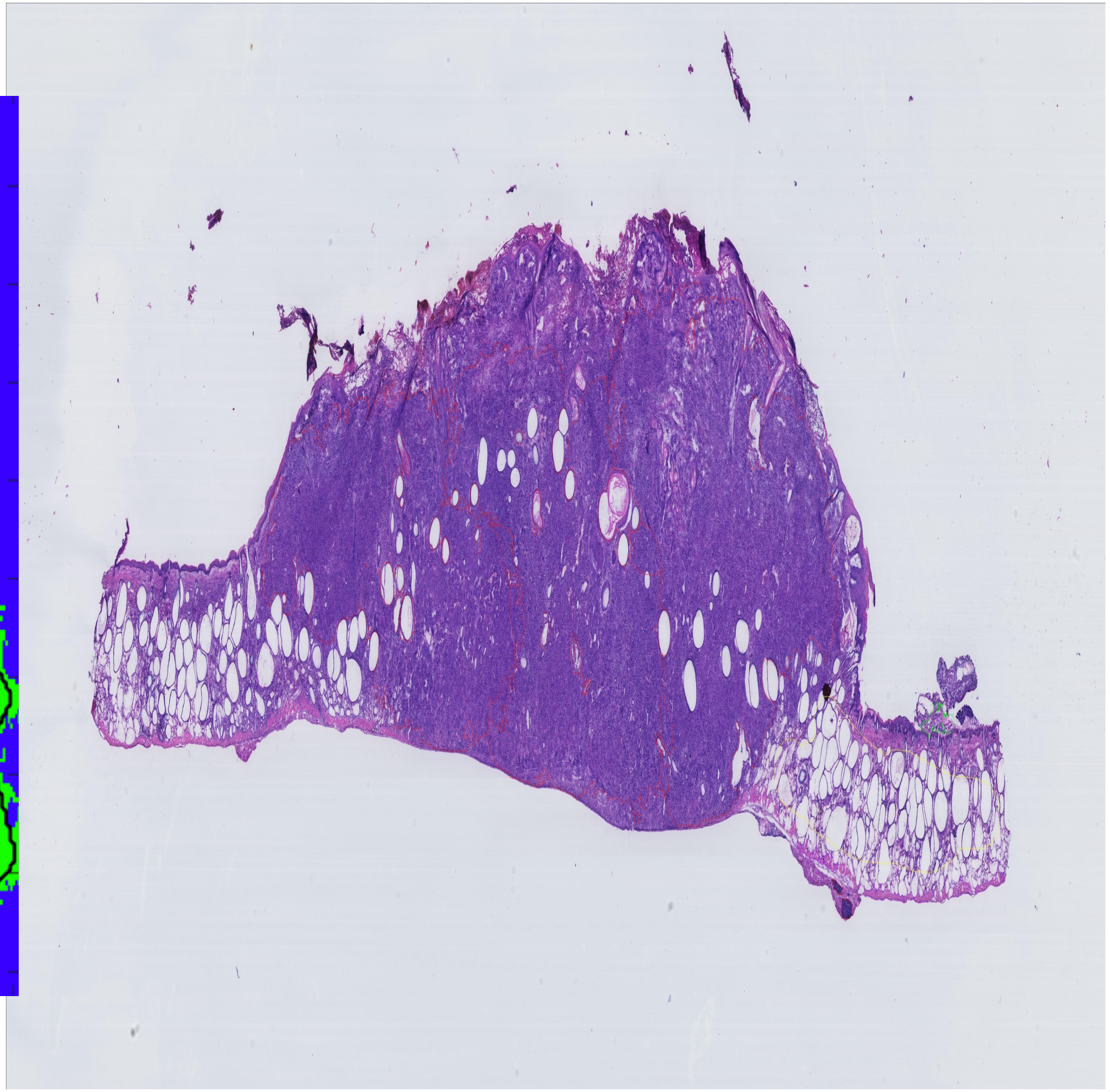

261t2

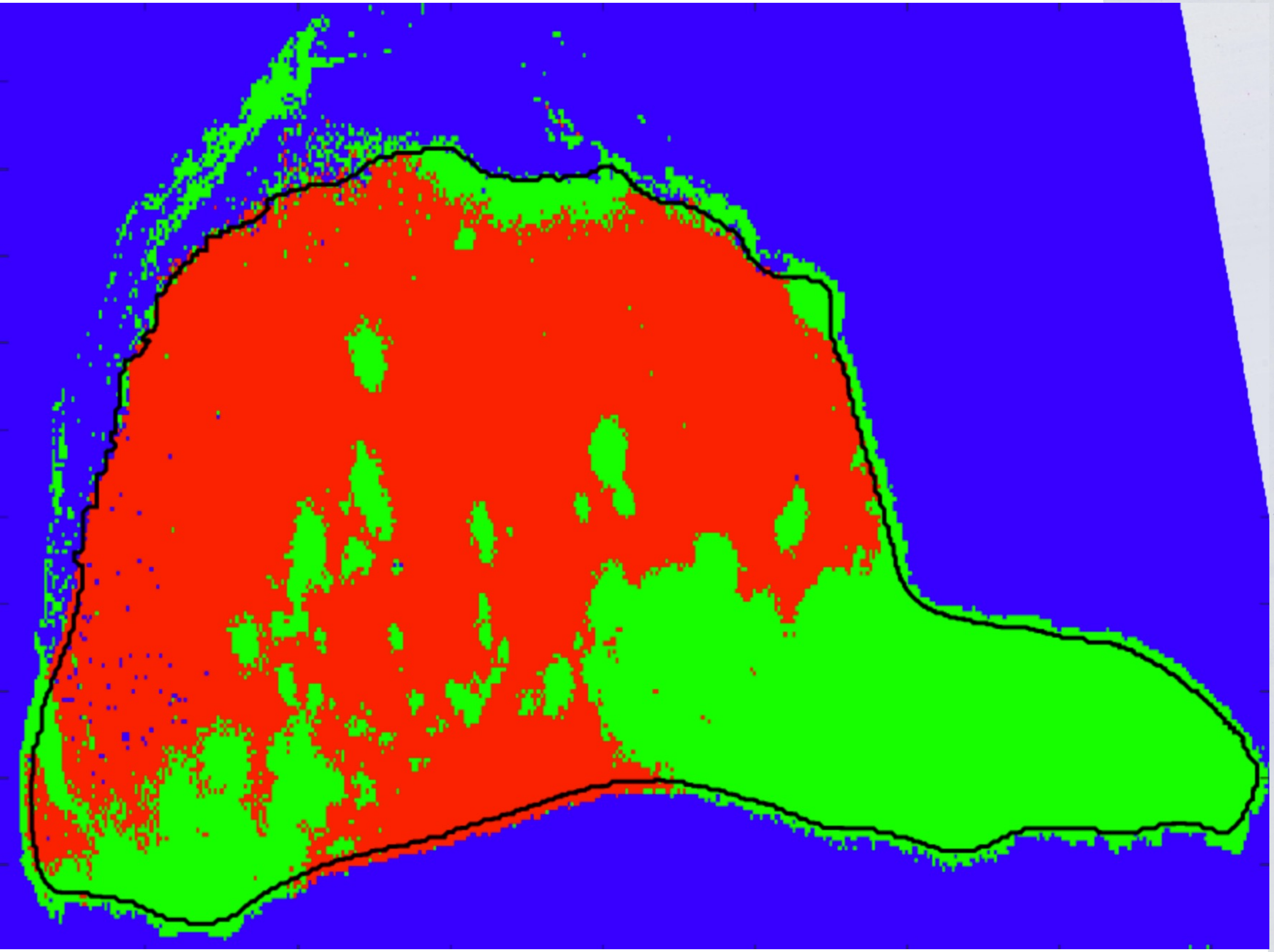

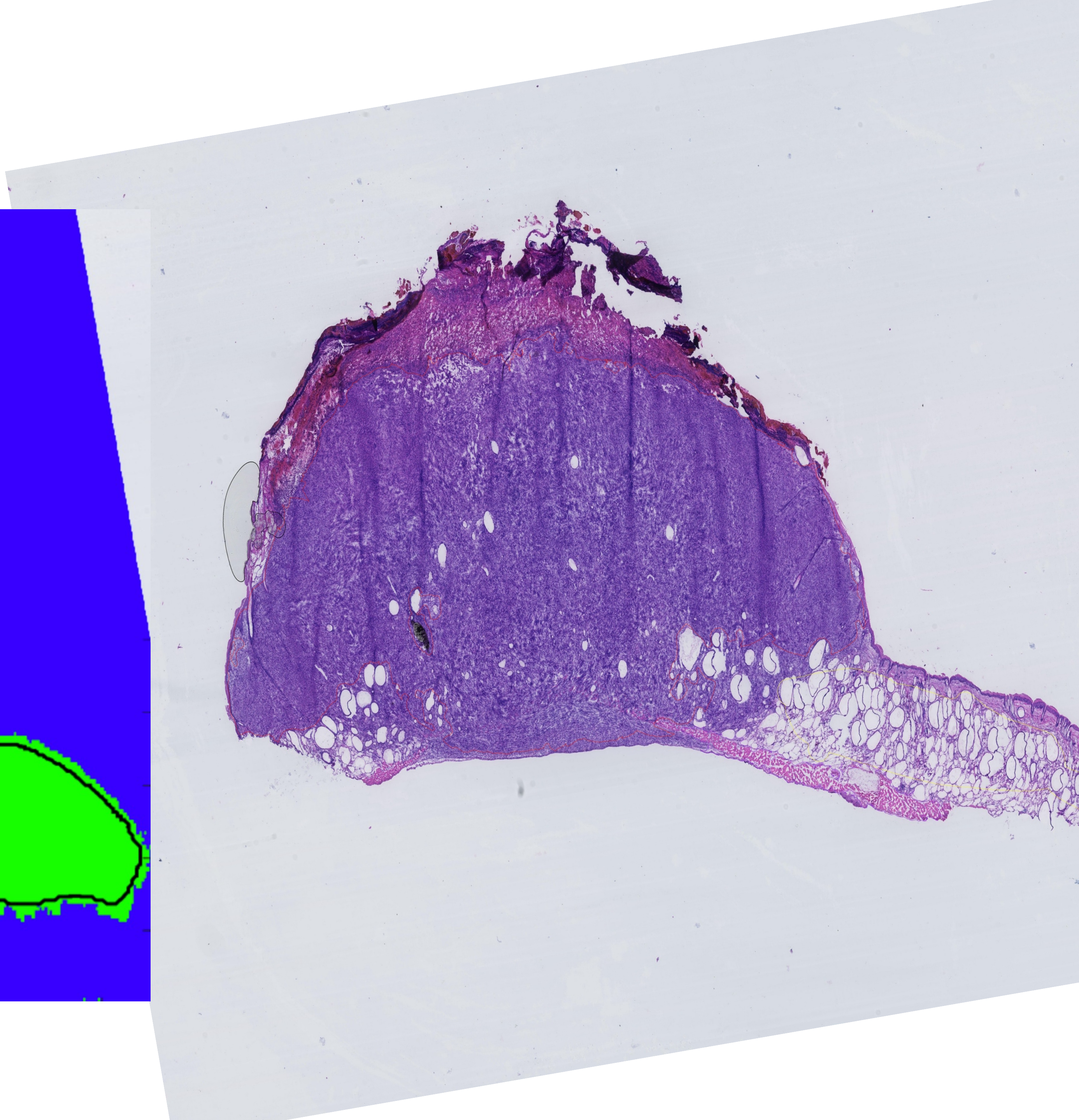

263

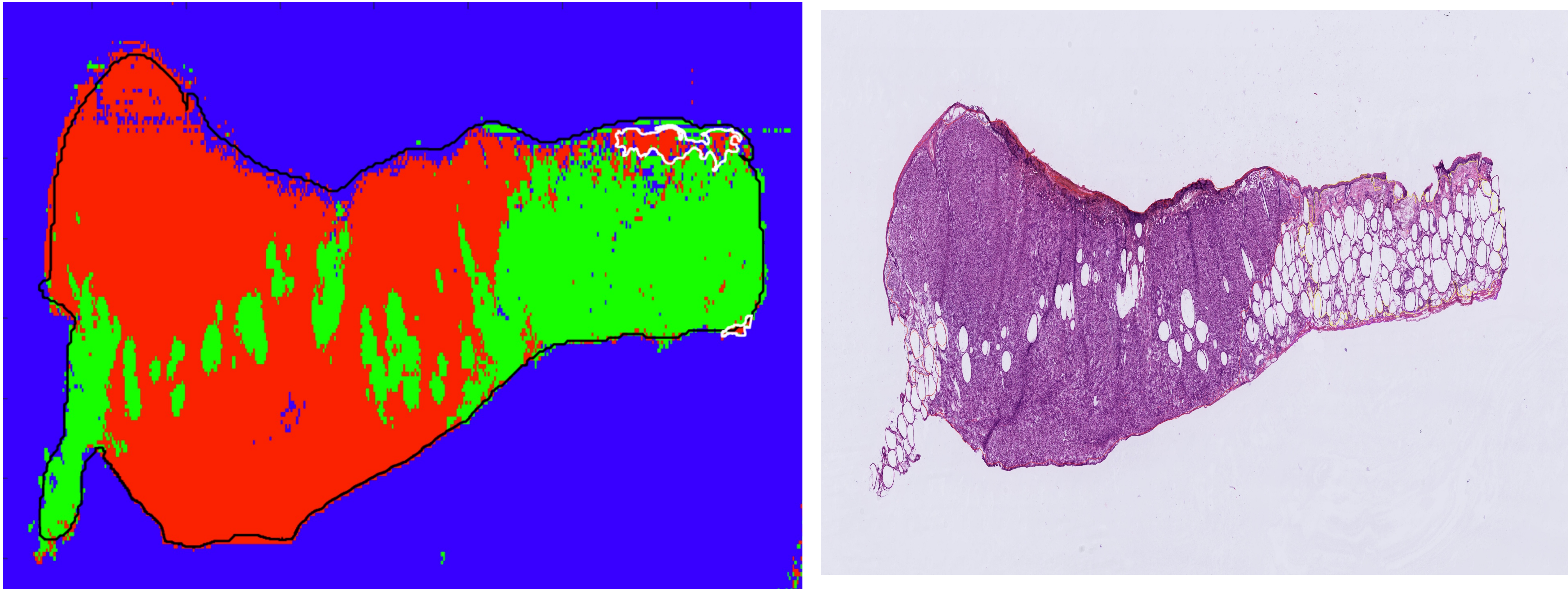

Gr4#6

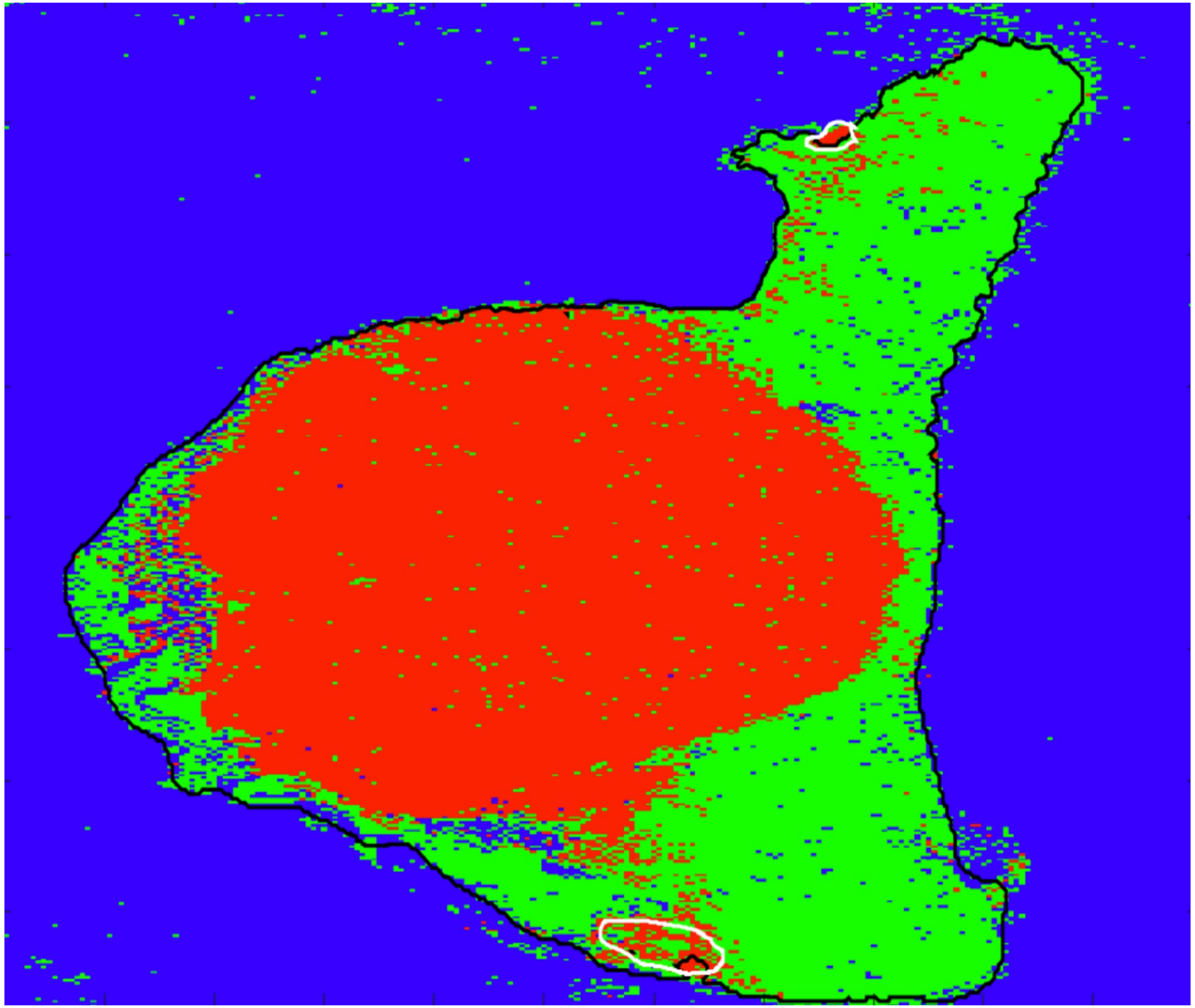

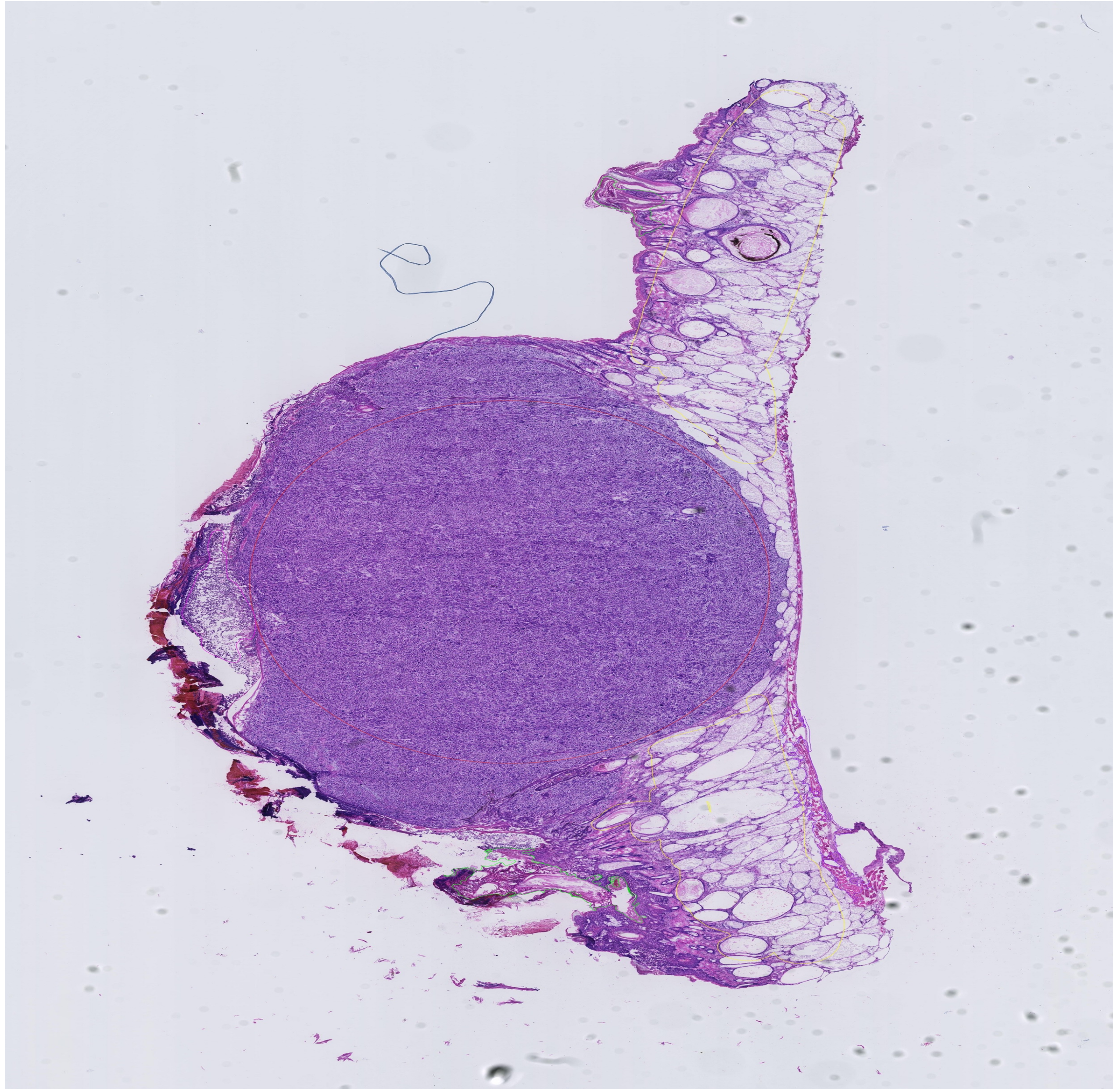

303t2

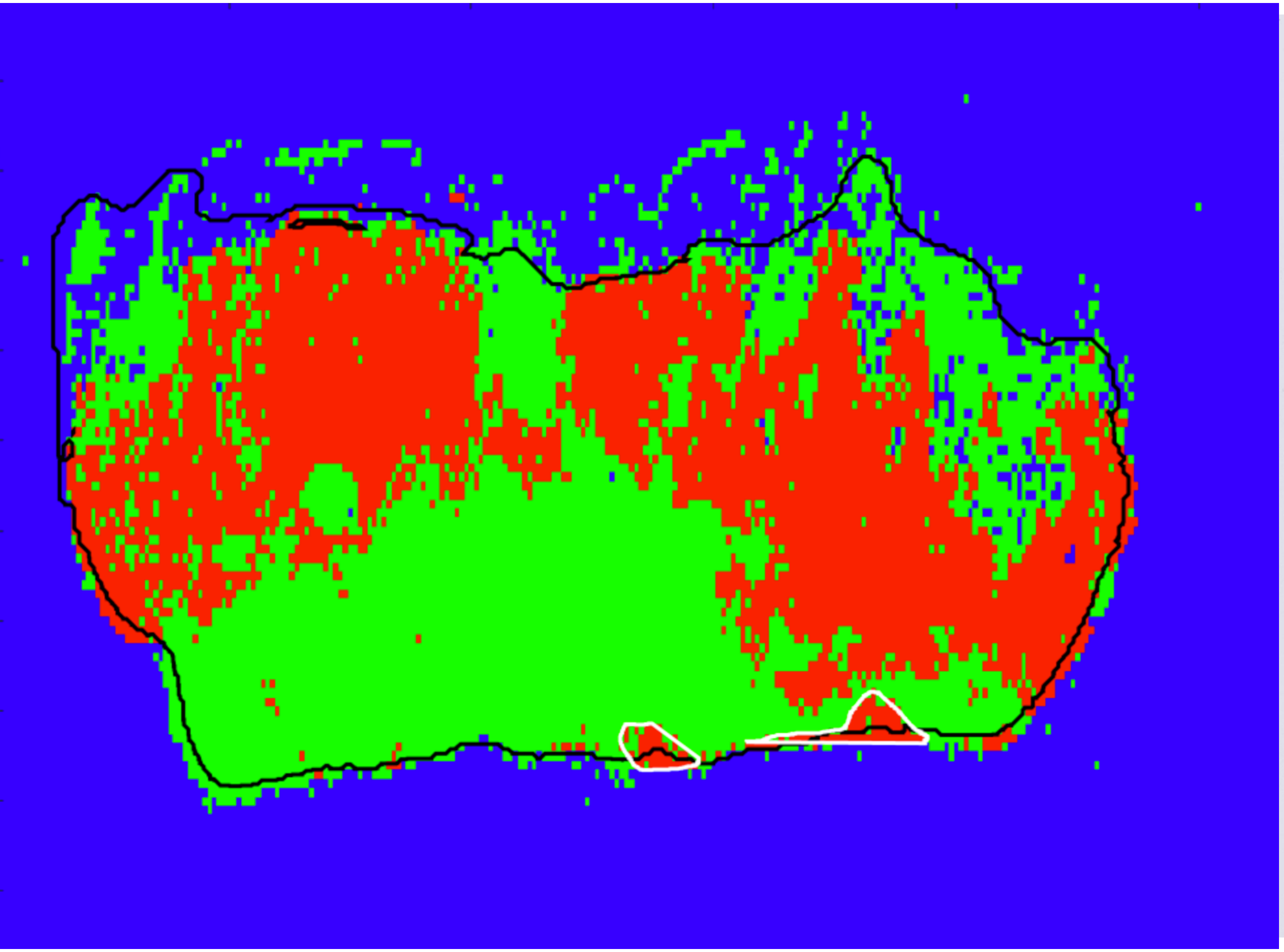

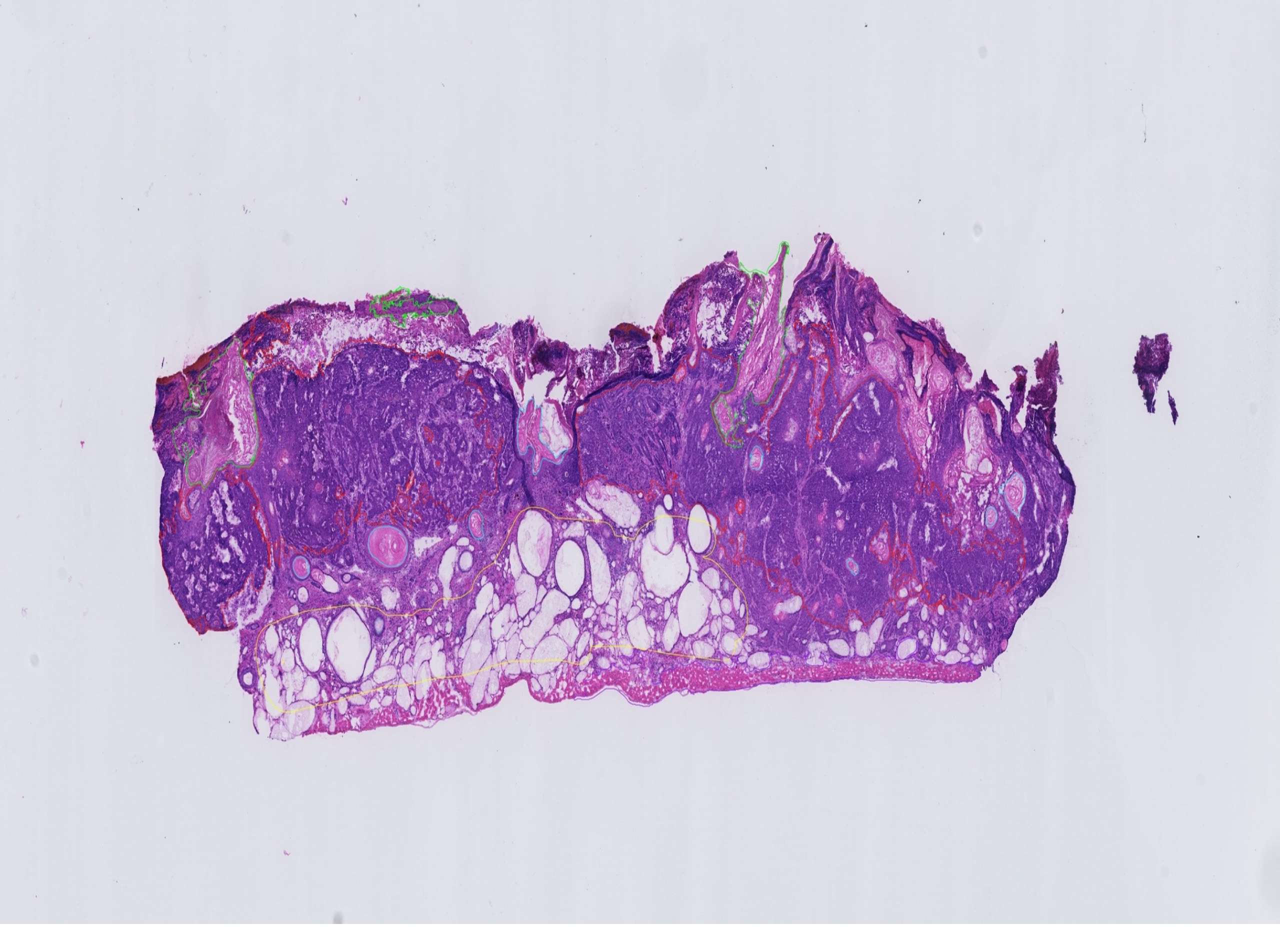

311t2

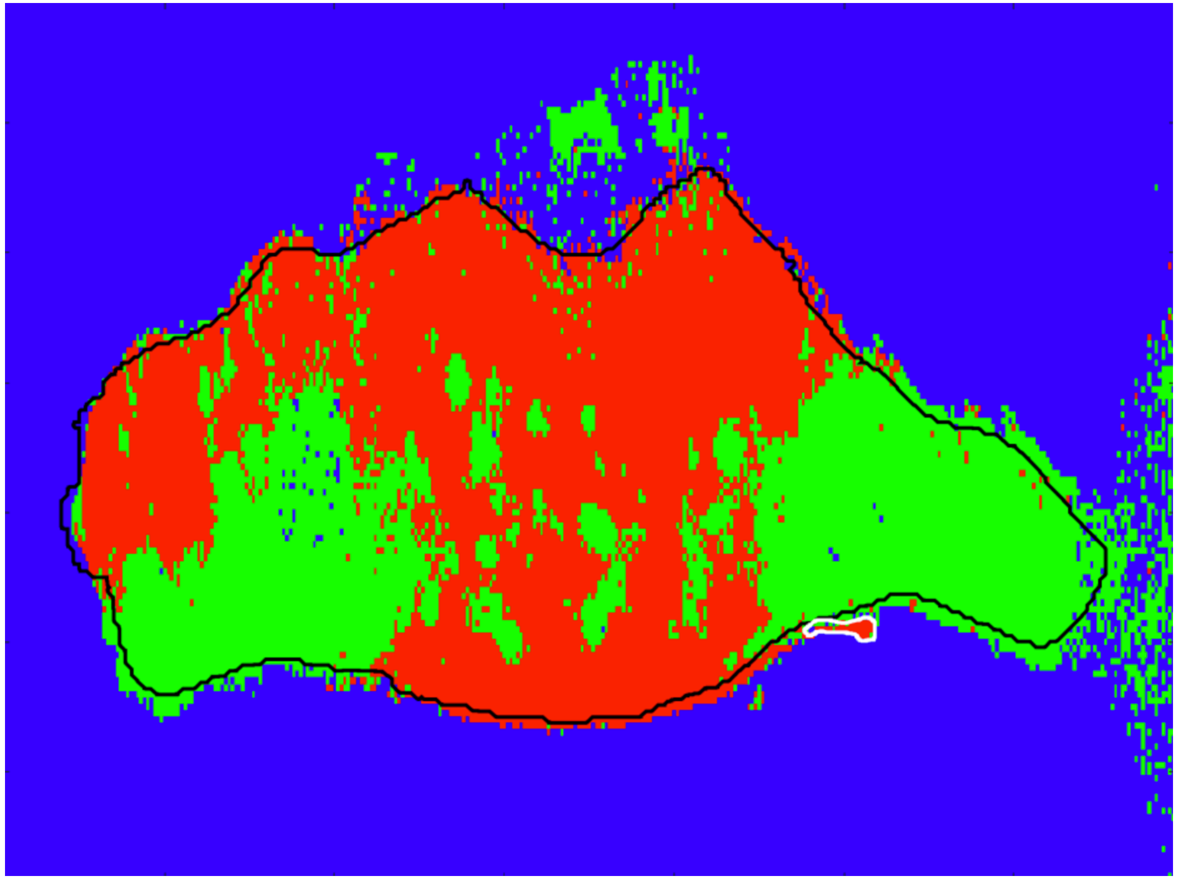

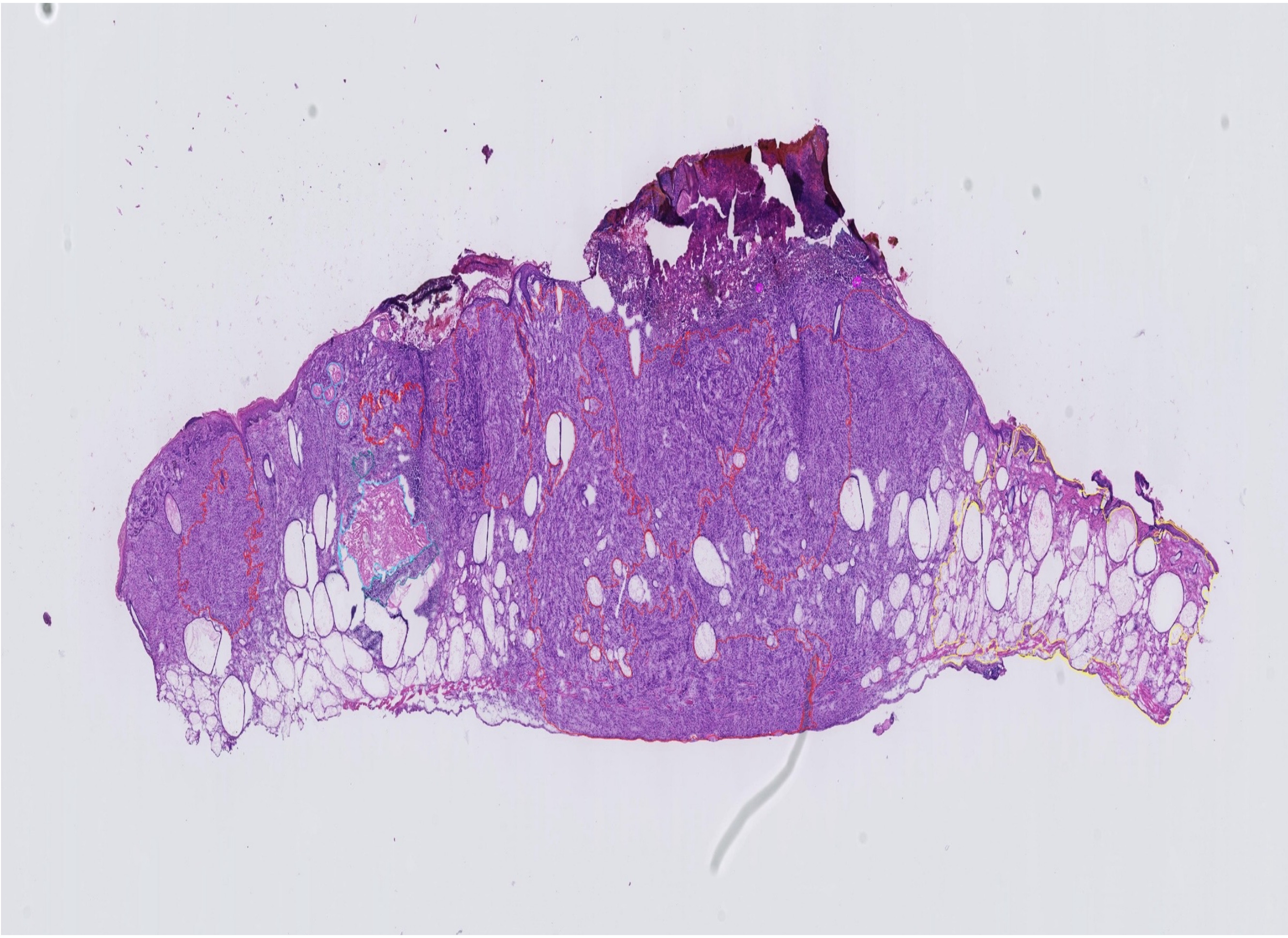

310t1

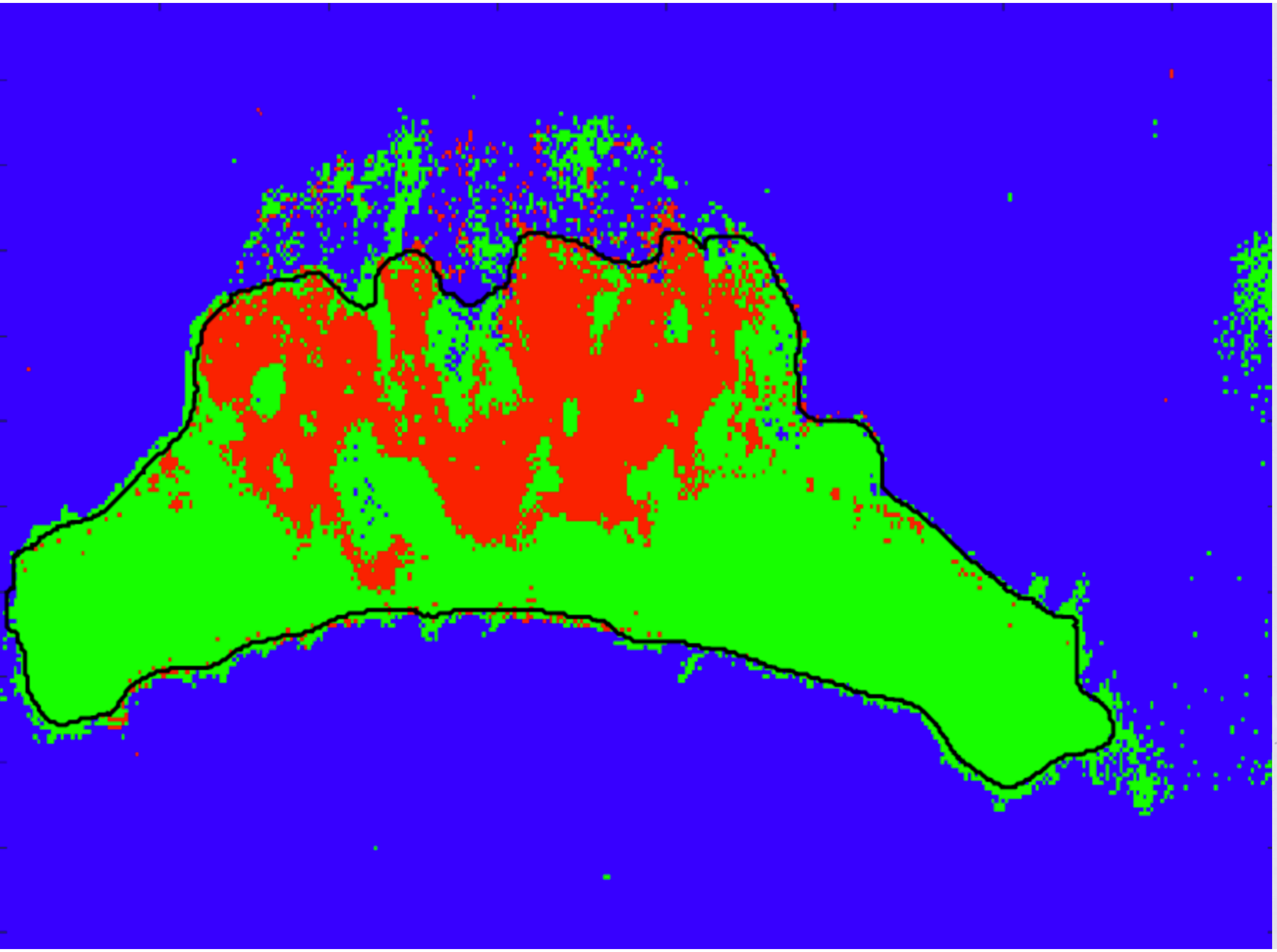

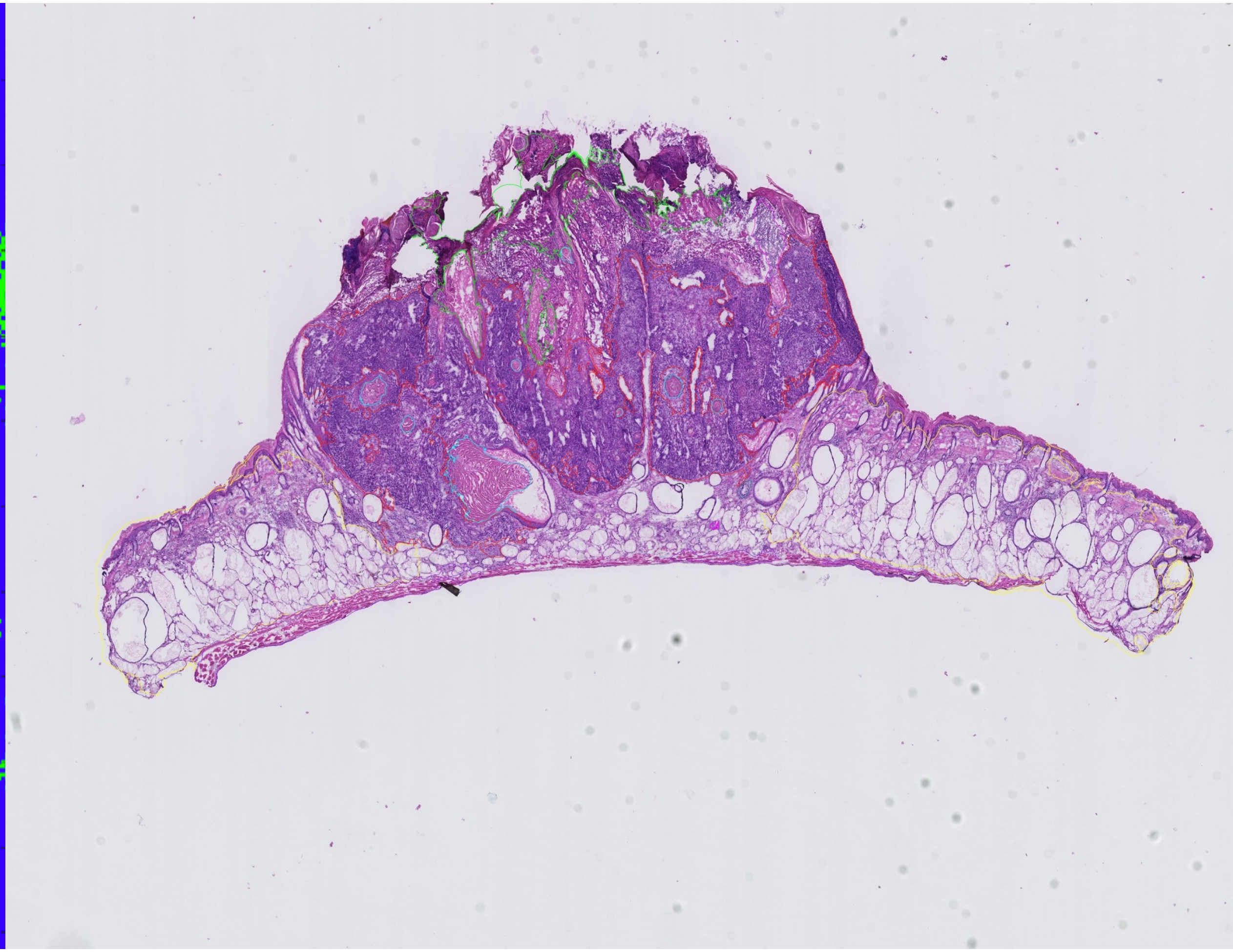

306t2

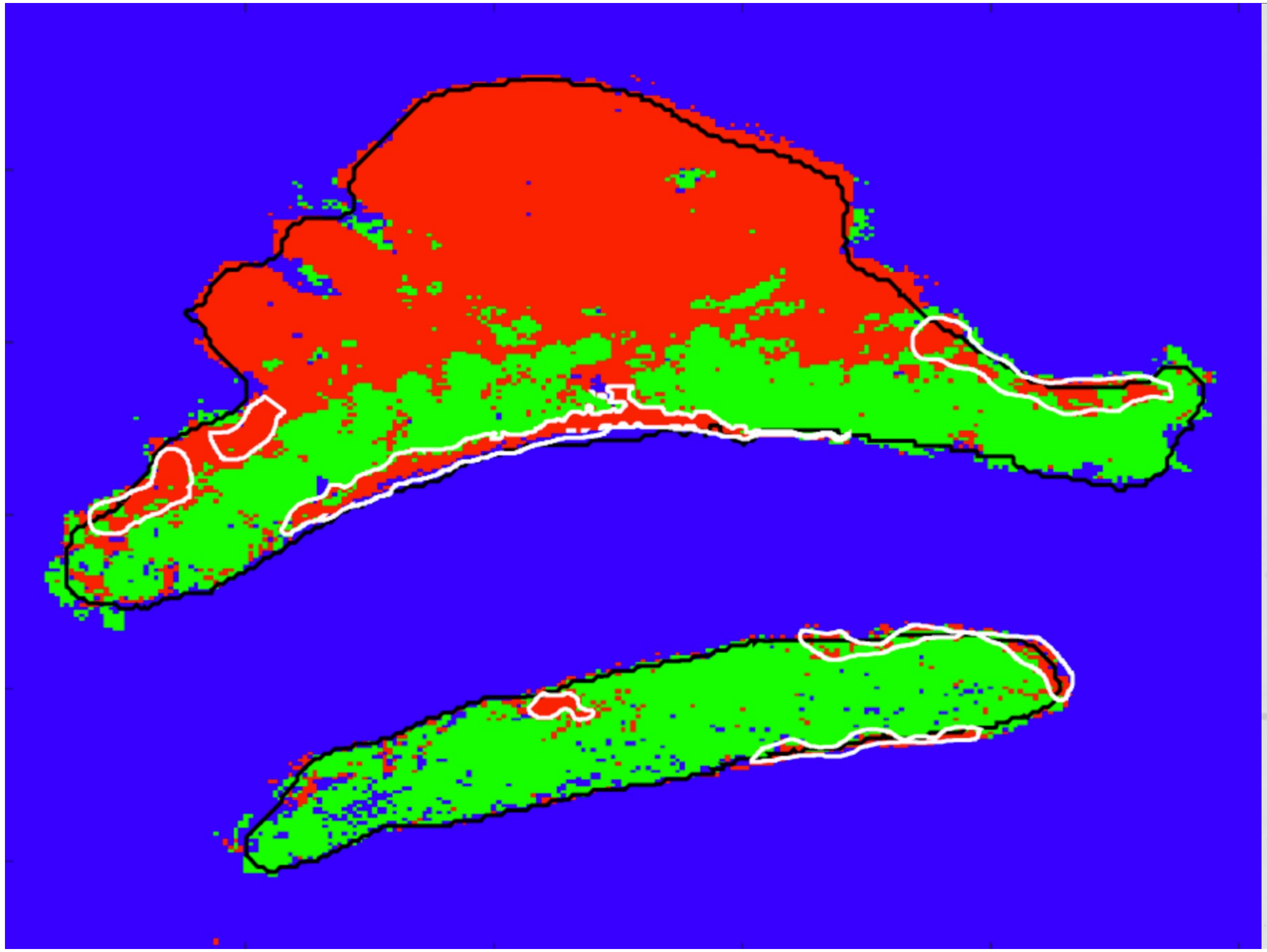

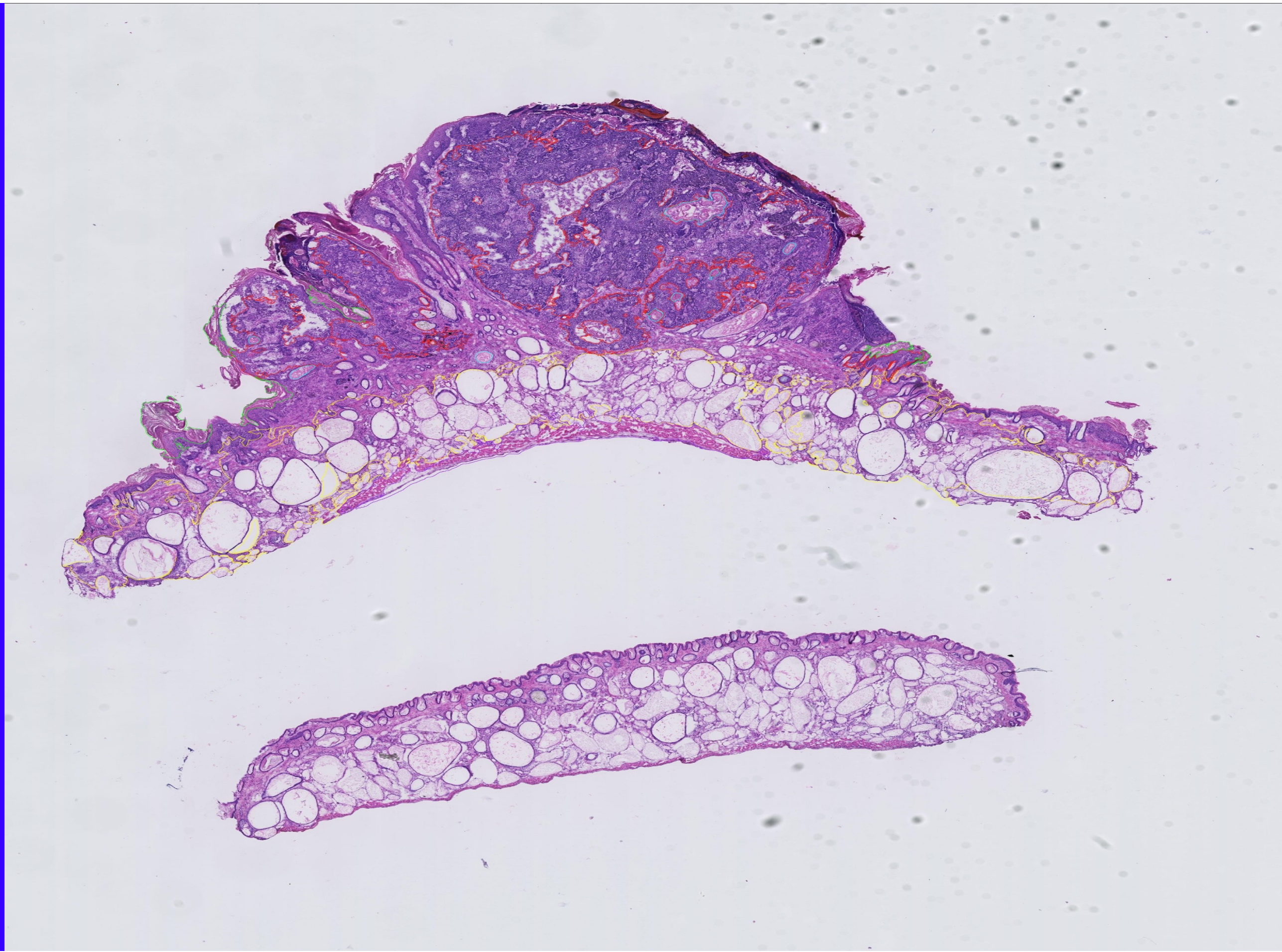

Gr4#23

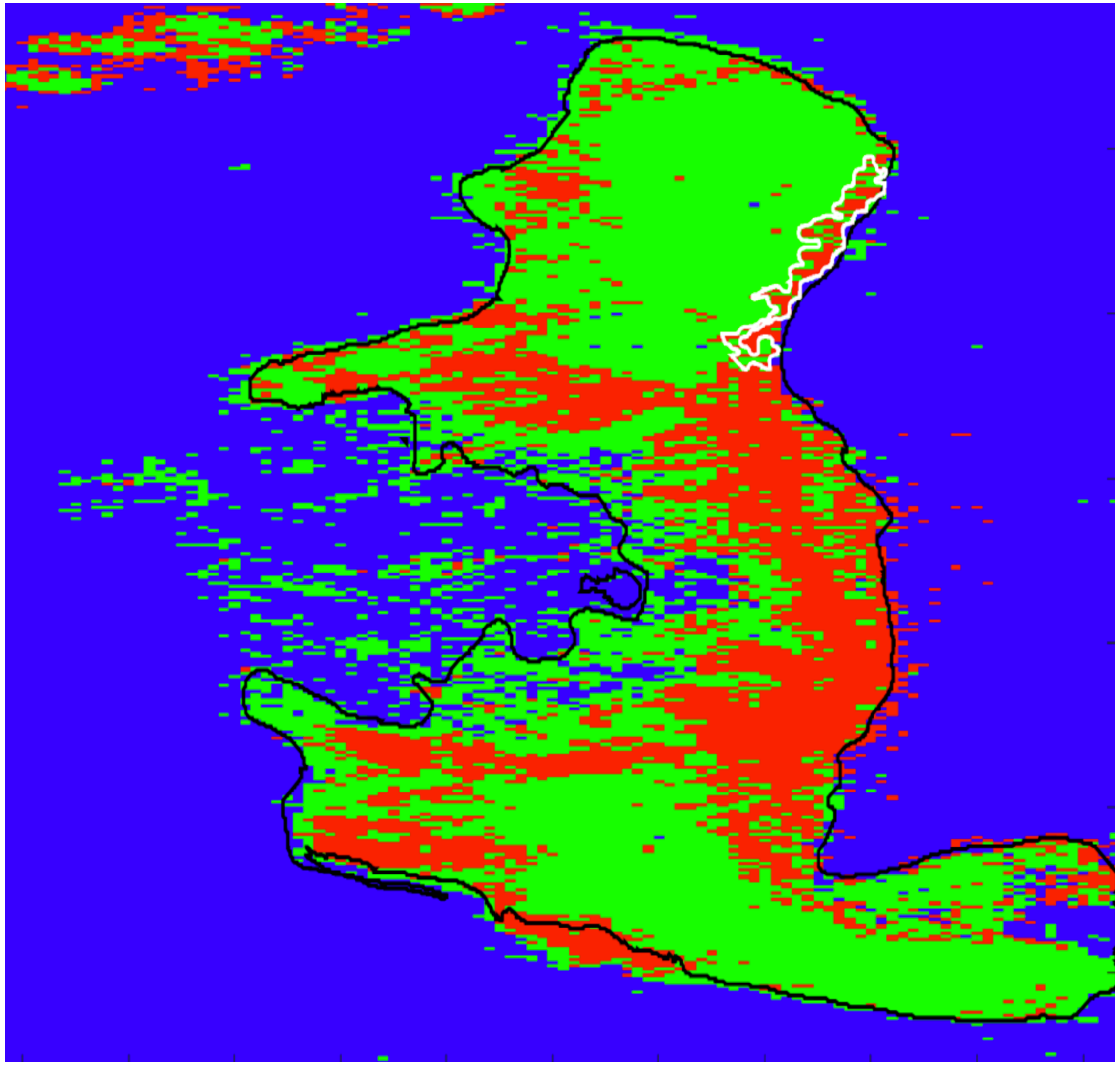

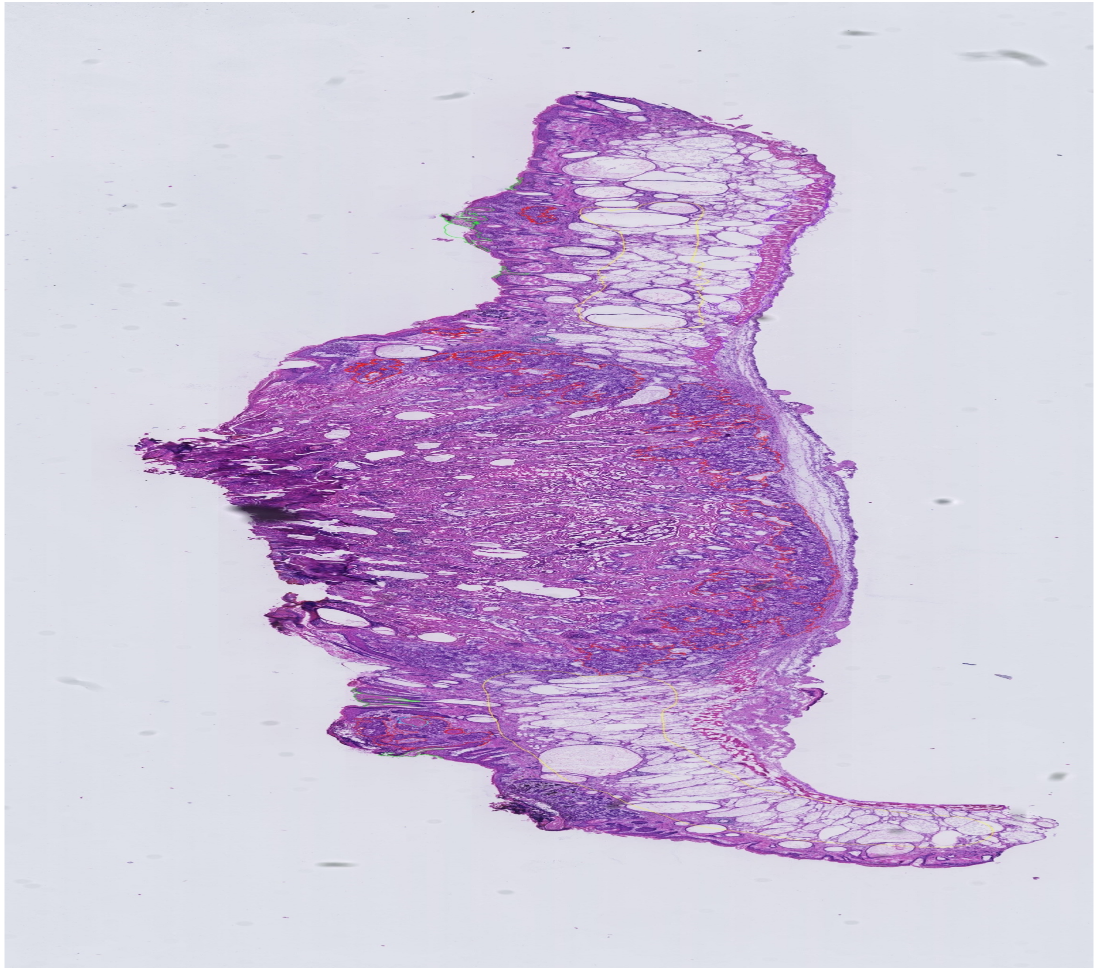

SCC 8

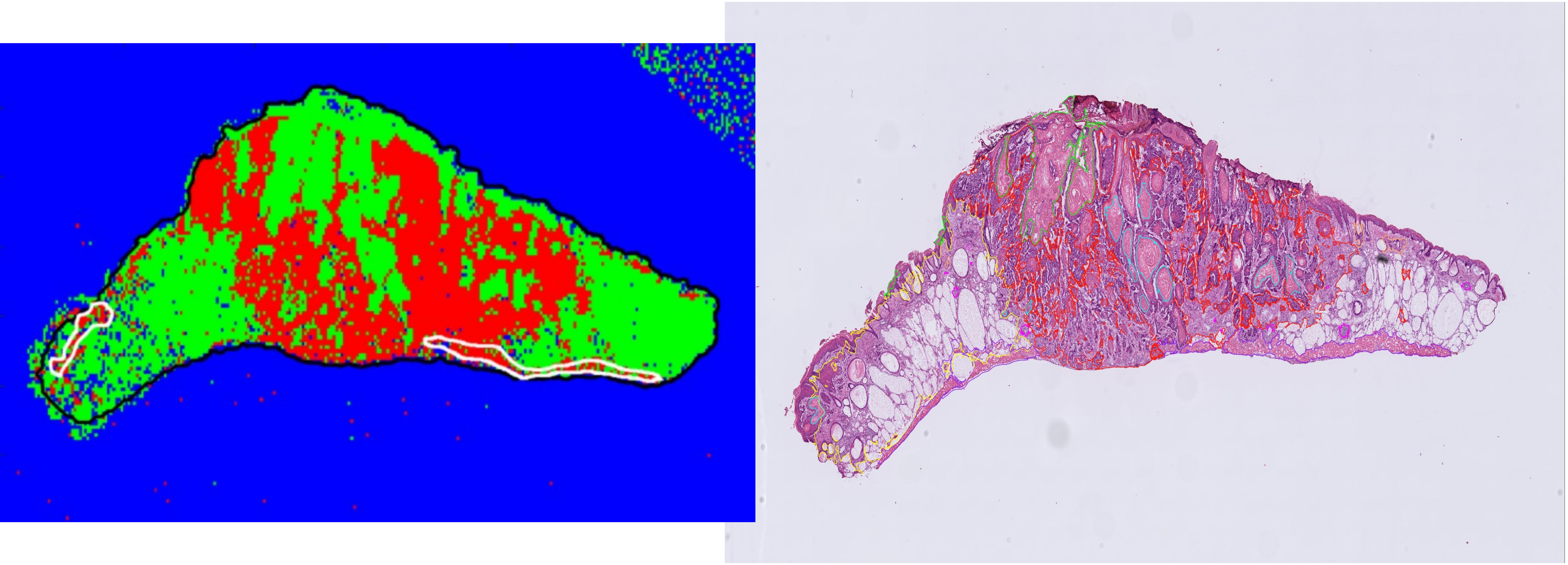

Supplement: Supplementary file 2 — Supplementary Information 2. [file 41598_2024_62023_MOESM2_ESM.pdf]
